# Oligomeric complexes formed by Redβ single strand annealing protein in its different DNA bound states

**DOI:** 10.1093/nar/gkab125

**Published:** 2021-03-08

**Authors:** Brian J Caldwell, Andrew Norris, Ekaterina Zakharova, Christopher E Smith, Carter T Wheat, Deepanshu Choudhary, Marcos Sotomayor, Vicki H Wysocki, Charles E Bell

**Affiliations:** Ohio State Biochemistry Program, The Ohio State University, Columbus, OH 43210, USA; Department of Biological Chemistry and Pharmacology, The Ohio State University, Columbus, OH 43210, USA; Department of Chemistry and Biochemistry, The Ohio State University, Columbus, OH 43210, USA; Department of Biological Chemistry and Pharmacology, The Ohio State University, Columbus, OH 43210, USA; Ohio State Biochemistry Program, The Ohio State University, Columbus, OH 43210, USA; Department of Biological Chemistry and Pharmacology, The Ohio State University, Columbus, OH 43210, USA; Ohio State Biochemistry Program, The Ohio State University, Columbus, OH 43210, USA; Department of Biological Chemistry and Pharmacology, The Ohio State University, Columbus, OH 43210, USA; Department of Chemistry and Biochemistry, The Ohio State University, Columbus, OH 43210, USA; Ohio State Biochemistry Program, The Ohio State University, Columbus, OH 43210, USA; Department of Chemistry and Biochemistry, The Ohio State University, Columbus, OH 43210, USA; Ohio State Biochemistry Program, The Ohio State University, Columbus, OH 43210, USA; Department of Chemistry and Biochemistry, The Ohio State University, Columbus, OH 43210, USA; Ohio State Biochemistry Program, The Ohio State University, Columbus, OH 43210, USA; Department of Biological Chemistry and Pharmacology, The Ohio State University, Columbus, OH 43210, USA; Department of Chemistry and Biochemistry, The Ohio State University, Columbus, OH 43210, USA

## Abstract

Redβ is a single strand annealing protein from bacteriophage λ that binds loosely to ssDNA, not at all to pre-formed dsDNA, but tightly to a duplex intermediate of annealing. As viewed by electron microscopy, Redβ forms oligomeric rings on ssDNA substrate, and helical filaments on the annealed duplex intermediate. However, it is not clear if these are the functional forms of the protein *in vivo*. We have used size-exclusion chromatography coupled with multi-angle light scattering, analytical ultracentrifugation and native mass spectrometry (nMS) to characterize the size of the oligomers formed by Redβ in its different DNA-bound states. The nMS data, which resolve species with the highest resolution, reveal that Redβ forms an oligomer of 12 subunits in the absence of DNA, complexes ranging from 4 to 14 subunits on 38-mer ssDNA, and a much more distinct and stable complex of 11 subunits on 38-mer annealed duplex. We also measure the concentration of Redβ in cells active for recombination and find it to range from 7 to 27 μM. Collectively, these data provide new insights into the dynamic nature of the complex on ssDNA, and the more stable and defined complex on annealed duplex.

## INTRODUCTION

Many bacteriophage encode a simple two protein recombination system for the repair of double-stranded DNA (dsDNA) breaks by a mechanism known as single strand annealing (SSA) (1,2). The most well-studied of these is the recombination deficient (Red) system from bacteriophage λ, which consists of a 5′-3′ exonuclease, λ Exo, to resect the 5′ strand of dsDNA ends (3,4), and a SSA protein, Redβ, which binds the resulting 3′ overhang to promote its annealing to a complementary strand from another DNA molecule (5,6). While the biological role of these recombination systems has been confusing, recent data suggest that they may have evolved as a mechanism of CRISPR evasion (7). Interest in these proteins also stems from their ability to promote SSA with complementary regions as short as 30–50 bases (8). Taking advantage of this property, the proteins have been exploited in powerful methods for bacterial genome engineering such as recombineering (9,10) and multiplex automated genome engineering (11). However, our understanding of the mechanism of Redβ in SSA is currently limited, primarily due to a lack of structural information.

Redβ is a 261 amino acid protein (monomer *M*_r_ of 29.7 kDa) that exhibits unusual and intriguing DNA binding behavior. It binds loosely to ssDNA, not at all to preformed dsDNA, but tightly to a duplex intermediate of annealing that is formed when two complementary oligonucleotides are added to the protein sequentially (12). Negative stain transmission electron microscopy (EM) of Redβ revealed that it forms structures appearing as oligomeric rings of 11–12 subunits without DNA, rings of 15–18 subunits with ssDNA and left-handed helical filaments when mixed with long (1.3 kb) heat denatured dsDNA (13). The latter complex presumably contains Redβ bound to some form of annealed duplex. The filaments often extend from rings, which led the authors to conclude that the annealing reaction may have started on a ring, and was extended by a filament.

**Figure 1.**
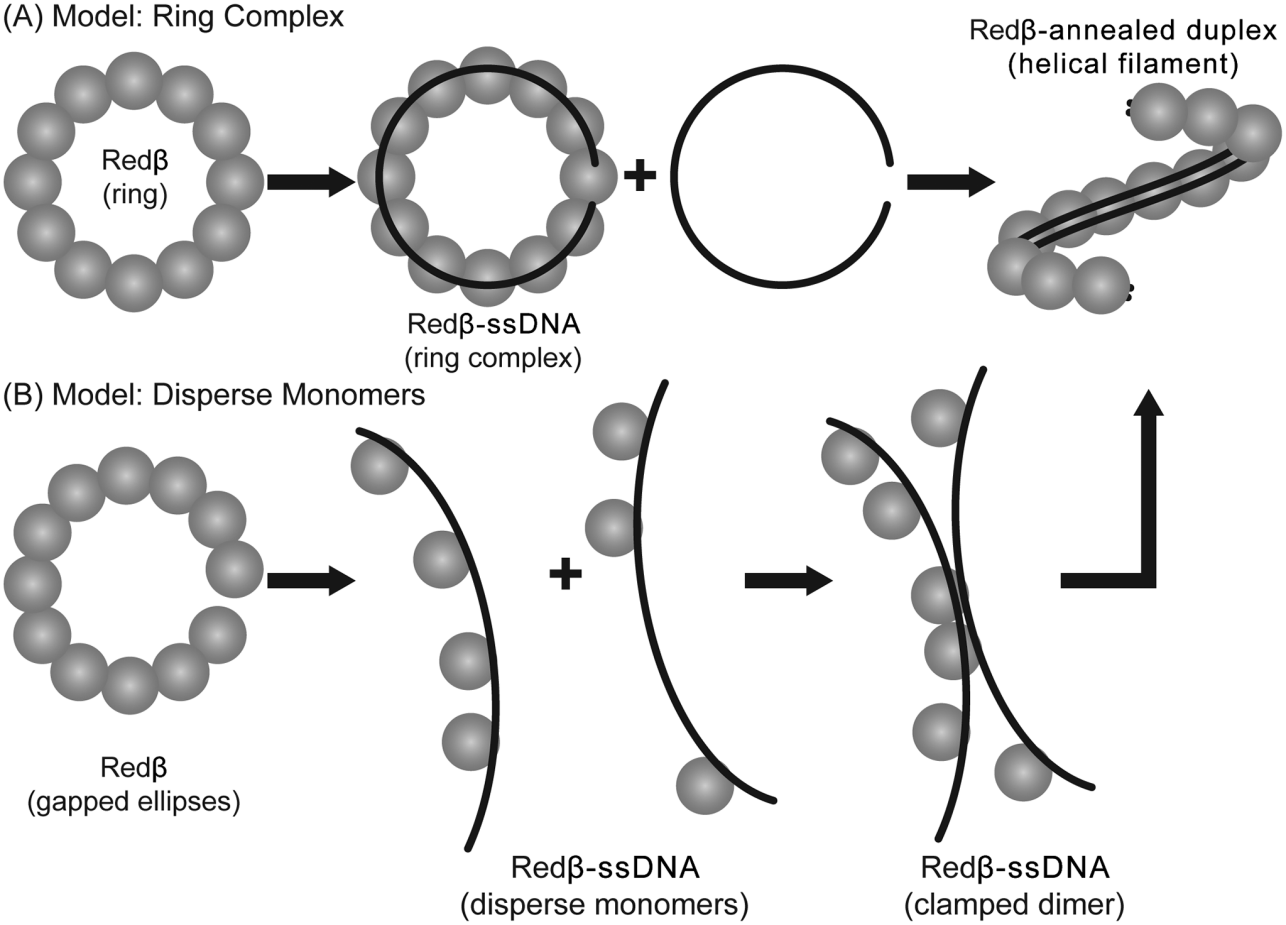
Models for Redβ’s mechanism of SSA. (**A**) Negative stain EM data indicate that Redβ forms oligomeric rings of 11–12 subunits in the absence of DNA, slightly larger rings when bound to ssDNA substrate, and left-handed helical filaments when bound to annealed duplex intermediate (13). The latter complex can be formed by sequential addition of two complementary oligonucleotides (12), or by mixing Redβ with long (1.2 kb) heat-denatured dsDNA (13). Following Passy *et al.* (13), the annealed duplex is shown as bound along the inner surface of the Redβ filament, based on the observation that it is protected from DNAse I cleavage (12). Overall, the ring model of annealing, which has also been proposed for Rad52 (19–20,48), posits that the protein exists as a stable oligomeric ring on which the initial ssDNA binding and annealing events take place. (**B**) Data from AFM on the other hand indicate that Redβ forms predominantly a split lock washer (or gapped ellipse) in the absence of DNA, disperse monomers on a 140-nt ssDNA, and left-handed helical filaments on different lengths of annealed duplex (16). Ander *et. al*. proposed that annealing is initially mediated by monomers of Redβ on the ssDNA that come together to form a stable clamped dimer when a 20 bp region of complementarity is encountered. Once formed, the clamped dimer nucleates formation of a helical filament on annealed duplex (25). In contrast to Passy *et al.* (13), Erler *et al.* modeled the annealed duplex as spiraling around the surface of the Redβ filament based on geometric considerations (as shown in Figure 6 of reference 16).

These data led to a model in which the multi-subunit ring form of Redβ binds to ssDNA and presents it in an extended conformation with the bases exposed for homology recognition (Figure 1A). To search for homology, this ring-ssDNA complex may interact with other such complexes, or with ssDNA bound by *Escherichia coli* single-stranded DNA-binding protein (SSB) at the lagging strand of a replication fork (14,15). Once an initial segment of complementary ssDNA has been located and aligned, the complex somehow transitions (or reassembles) into a helical filament to bind the annealed duplex as it is being formed (13). Tighter binding of Redβ to the annealed duplex intermediate than to the ssDNA substrate presumably drives the annealing reaction forward (12). In agreement with this model, the crystal structure of the N-terminal DNA binding domain of human Rad52, a SSA protein that is distantly homologous to Redβ (16–18), revealed an 11-mer ring with a narrow positively charged groove that could accommodate ssDNA but not dsDNA (19,20). Moreover, a wide variety of other SSA proteins form similar oligomeric rings, including phage P22 Erf (21), *E. coli* RecT (22), Sak protein of *lactoccocal* phage ul36 (23) and mitochondrial Mgm101 (24), suggesting that the ring may be important for annealing. The helical filaments seen for Redβ on the annealed duplex intermediate, however, have generally not been seen for these other proteins.

While this model for DNA binding and annealing is compelling, not all of the data are in agreement. When viewed by atomic force microscopy (AFM), Redβ forms primarily a split-lock washer (but also some closed rings) of 11–12 subunits without DNA, disperse monomers when bound to a 140-mer ssDNA, and helical filaments when bound to complementary ssDNAs ranging from 83- to 163-nt in length (16). While the structures of Redβ alone and with annealed duplex are similar to those seen by EM, the observation that Redβ binds to ssDNA as disperse monomers suggested that initial ssDNA binding and annealing might not occur on an oligomeric ring. Moreover, it has been questioned if the oligomers formed by Redβ *in vitro* are even present at physiological concentrations of the protein *in vivo*. Quantitative western blotting demonstrated that the concentration of Redβ in cells active for recombination can be <0.15 μM (25). This is far below the concentrations at which oligomer formation has been seen by gel-filtration (30 μM) and EM (0.8–3.6 μM) (13,26–28). Fluorescence correlation spectroscopy of labeled Redβ and ssDNA revealed that Redβ is predominantly monomeric at the *in vivo* concentrations identified by quantitative western blotting, and only forms oligomers at concentrations above 1 μM (25). Furthermore, cross-correlation of the signals from fluorescent probes on Redβ and ssDNA, combined with a quantitative model for Redβ oligomerization and DNA binding indicated that the ssDNA binding transition occurs at submicromolar concentrations of Redβ, where the protein is predominantly monomeric. Finally, single-molecule experiments with optical tweezers showed that the remarkably stable complex responsible for DNA annealing could be formed on an annealed duplex as short as 25 bp, which was purported to be bound by only two Redβ monomers (25).

Based on these new observations, particularly the observation that Redβ binds to ssDNA as disperse monomers, a new model for annealing was proposed in which a complex between a monomer of Redβ and a ssDNA could sample and weakly associate with about 10 nucleotides of complementary sequence from a second ssDNA (Figure 1B). Annealing of this initial seed region is further stabilized when a second monomer of Redβ enters the complex and binds to an adjacent sequence of complementary DNA to form a stably clamped dimer. A key feature of this model is that a structural change takes place when the relatively weak complex of Redβ on ssDNA (with an unbinding force of 12 pN) transitions to the much more stable dimer of Redβ on the annealed duplex intermediate (with an unbinding force of 200 pN) (25). According to this model, the oligomeric rings of Redβ observed at higher concentrations *in vitro* are not relevant at physiological concentrations of the protein *in vivo*. Rather, the inter-subunit interactions that form the rings seen *in vitro* may simply reflect the lateral interactions between two adjacent monomers in a clamped dimer.

While this new model is compelling, it remains unclear why such a conserved oligomeric ring of 8–14 subunits is formed by so many of the SSA proteins that have been studied (19–24). In addition, many properties of the oligomers formed by Redβ in its different DNA bound states have not been fully characterized, such as their precise numbers of subunits, number of nucleotides (or base pairs) bound per monomer and dependencies on physiological ionic strength and protein concentration. In an attempt to clarify these issues, we have used size-exclusion chromatography coupled with multi-angle light scattering (SEC-MALS), analytical ultracentrifugation (AUC) by sedimentation velocity (SV) and native mass spectrometry (nMS) to characterize the sizes of the oligomeric complexes formed by Redβ alone, with ssDNA substrate, and with annealed duplex intermediate. While dynamic light scattering (DLS) of Redβ has been reported previously (26), to our knowledge, mass analysis by AUC or nMS has not yet been reported. nMS in particular can provide masses that are accurate enough to define the precise number of subunits in a given complex, and to distinguish between two or more closely sized species such as 11- and 12-mers. Combined with these *in vitro* methods, we have also re-examined the concentration of Redβ expressed in cells by two systems commonly used for recombineering. Collectively, at the *in vivo* concentrations of 7–27 μM determined here, the three methods reveal that Redβ forms a relatively uniform oligomer of 12 subunits in the absence of DNA, a broad distribution of oligomers ranging from 4 to 14 subunits on 38-mer ssDNA, and a distinct and more stable complex of 11 subunits on 38-mer annealed duplex. Complexes on 83-mer oligonucleotides provide further insights that are discussed. Collectively, the data are consistent with a model in which the initial ssDNA is bound not by a distinct oligomeric species of the protein such as a closed 11-mer ring, but rather by a more dynamic and variable assembly of monomers on the ssDNA.

## MATERIALS AND METHODS

### Western blots to measure the concentration of Redβ expressed *in vivo*

The *in vivo* concentration of Redβ was measured by quantitative western blot for two different recombineering systems, one that expresses the λ red functions (Redβ, λ Exo, Gam) from a pSIM5 plasmid transformed into HME57 cells (29), and the second from a pSC101-based plasmid transformed into GB2005 cells containing a bacterial artificial chromosome with chloramphenicol resistance (30). For both systems, the cells were treated in the same manner as for their respective recombineering protocols, except in the case of pSC101 where a larger culture volume (50 ml) was used to get enough material to sonicate and isolate the soluble fraction (published protocols for pSC101 range from 1.4 to 30 ml of cell culture, 30–33). Plasmids were transformed into their respective cell lines by electroporation followed by recovery and plating on LB agar containing 30 μg/ml chloramphenicol (Cm^30^) for pSIM5, or 10 μg/ml chloramphenicol and 4 μg/ml tetracycline (Cm^10^Tet^4^) for pSC101. For pSIM5, a single colony was inoculated into a 5 ml overnight culture, diluted to a starting OD_600_ of 0.09 in 35 ml of LB media, grown to an OD_600_ of 0.4 at 32°C, and induced by shaking at 42°C for 15 min. pSC101 was treated in the same manner, but the overnight culture was diluted into 50 ml of LB media, grown at 30°C to an OD_600_ of 0.4, and induced with 0.2% arabinose followed by shaking at 37°C for 45 min. After the induction period, the cultures were placed on wet ice for 10 min and the OD_600_ of the cultures was measured. Cultures were harvested by centrifugation at 5000 × *g* for 10 min at 4°C, re-suspended in 2.0–2.5 ml of sonication buffer (50 mM NaH_2_PO_4_, 300 mM NaCl, 10 mM imidazole, pH 8.0), and frozen at −80°C.

**Figure 2.**
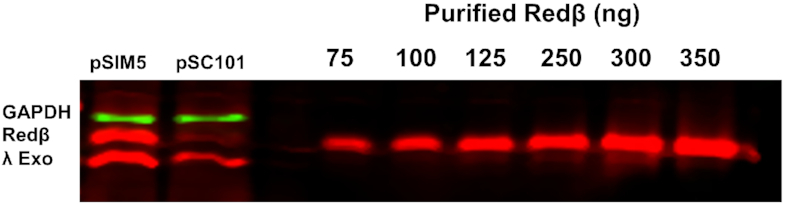
Western blot to determine the concentration of Redβ expressed in cells active for recombination. Lanes labeled pSIM5 and pSC101 contain 10 μl of the soluble cell lysates prepared from the respective cell cultures as described in ‘Materials and Methods’ section. Lanes to the right contain the indicated amounts of purified Redβ. Notice that while the green bands for GAPDH indicate similar expression of this control protein from pSIM5 and pSC101, the bands for Redβ indicate significantly higher expression from pSIM5. From quantitative comparisons to standard curves generated from purified Redβ, multiple experiments for pSIM5 and pSC101 resulted in average *in vivo* Redβ concentrations of 27 ± 12 and 7 ± 2 μM (0.81 and 0.20 mg/ml), respectively, as shown in Supplementary Figure S1.

After thawing, the 2.0–2.5 ml cell suspension was lysed at 4°C with a Branson sonicator using a microtip at 50% power and 30% duty for 3 × 1 min. The crude lysate from sonication was centrifuged for 30 min at 13 000 rpm in an Eppendorf microcentrifuge at 4°C, and the supernatant containing the soluble cell lysate was frozen in 500 μl aliquots at −80°C. After thawing, 10 μl of each lysate was mixed with 10 μl of 5× SDS-PAGE loading buffer, heated for 5 min at 95°C, and loaded at 10–16 μl onto a 12.5% SDS-PAGE gel. For quantification, purified Redβ protein (as described below) was loaded in amounts ranging from 25 to 350 ng. After electrophoresis, protein bands were transferred to nitrocellulose membranes (Bio-Rad) in Transfer Buffer (25 mM Tris pH 8.3, 192 mM glycine, 0.05% SDS, 20% methanol) for 70 min at 90 V (constant voltage) and 4°C. Membranes were blocked with Fluorescent Blocker (Millipore Sigma) for 1.5 h at 22°C, and incubated with anti-Redβ-anti-λ Exo rabbit primary antibody, gifted from Dr. Kenan Murphy (UMass Medical School), in Fluorescent Blocker overnight at 4°C. Membranes were then washed three times for 15 min in 1× PBS (137 mM NaCl, 2.7 mM KCl, 10 mM Na_2_HPO_4_, and 2 mM KH_2_PO_4_ pH 7.4), 0.1% Tween® 20. Membranes were then incubated with IRDye 680RD donkey anti-Rabbit secondary antibody (Li-COR) in Fluorescent Blocker for 45 min at 22 to 25°C, and washed again for 3 × 15 min in 1× PBS, 0.1% Tween® 20. Final membranes were scanned for infrared fluorescence at 700 nm using an Odyssey Blot Imager (LI-COR), and quantified using LI-COR Image Studio software. The gel in Figure 2 (and upper left of Supplementary Figure S1) was also incubated with antibodies to stain for GAPDH, which appears in green. These were mouse anti-GAPDH primary monoclonal antibody (Thermo Fisher Scientific) and IRDye 800 donkey anti-mouse secondary antibody (Li-COR). This gel was scanned at both 700 and 800 nm.

Signal intensity quantification used bands outlined by rectangles of equal area, with the signal adjusted by median background subtraction (standard method) based on the formula:

$$\begin{equation*}{\rm Signal} = {\rm Total\, Signal}-({\rm Background} \times {\rm Area}),\,{\rm where}\end{equation*}$$

Total signal is the sum of pixel intensities within the rectangle, area is the total number of pixels in the rectangle, and background is the median intensity of pixels within a 3-pixel border around the rectangle. The amount of protein (in ng) from each band, determined by linear regression against signal from lanes containing known amounts of purified Redβ protein, was used to determine the amount of protein in the full 2.0–2.5 ml lysate. From this value, the concentration of Redβ expressed in cells was determined from the number of cells in the culture (from the measured OD_600_ value assuming 8.0 × 10^8^ cells per ml per OD), the volume of an *E. coli* cell, which was taken as 3.8 × 10^−9^ μl (34), and the MW of native Redβ calculated from its amino acid sequence (29 689 g/mol), as detailed in Supplementary Figure S1 and Supplementary Table S1.

### Protein purification

Redβ protein was expressed and purified as described previously (27,28). Briefly, the gene was cloned between the *Nde*I and *Bam*HI sites of pET28b to express an N-terminally 6-His tagged protein with a site for thrombin cleavage. The resulting plasmid was transformed into BL21(AI) *E. coli* cells, which were grown at 37°C in 3 × 1 L cultures to an OD_600_ of 0.5. After induction with 0.2% arabinose and 0.2 mM isopropyl β-D-1-thiogalactopyranoside (IPTG), cultures were grown for an additional 4 h at 37°C and centrifuged at 10 000 × *g*. Cell pellets were re-suspended in 50 ml of sonication buffer and frozen at −80°C. After thawing, the cell suspension was incubated on ice for 1 h with 1 mg/ml lysozyme, 1 μg/ml leupeptin and pepstatin, 1 mM PMSF, and lysed by sonication on ice for 3 × 3-min at full power, 30% duty. After centrifuging three times for 30 min at 48 000 × *g*, the clarified supernatant was loaded at 4°C onto two connected 5 ml Ni-NTA HisTrap columns (GE Healthcare), and washed extensively with 200 ml of sonication buffer. After elution in sonication buffer with a linear gradient from 10 to 500 mM imidazole, fractions containing purified 6His-Redβ were pooled, mixed with 100 units of thrombin (GE Healthcare), and dialyzed overnight at 22°C into 4 L of Thrombin Cleavage Buffer (20 mM NaH_2_PO_4_, 200 mM NaCl, pH 7.4). The solution of cleaved Redβ was centrifuged at 10 000 × *g* for 10 min, and loaded back onto the Ni-NTA HisTrap column to remove any remaining uncleaved 6His-tagged Redβ. The untagged Redβ was eluted with 30 mM imidazole, and the resulting fractions were dialyzed into 20 mM Tris pH 8.0 and further purified by anion exchange chromatography (Q sepharose FF, GE Healthcare). Pooled fractions were dialyzed into 20 mM Tris pH 8, concentrated to 48 mg/ml (Vivaspin 20, MWCO 10 kDa), and stored in 100 μl aliquots at −80°C. The final purified protein contains an extra Gly-Ser-His sequence at its N-terminus after thrombin cleavage. This alteration does not affect the activity of the protein by several tests *in vitro* and *in vivo* (27). All experiments performed in this study used Redβ protein diluted from this 48 mg/ml stock. The purity of this protein by SDS-PAGE is shown in Supplementary Figure S2. All protein concentrations were determined from the OD at 280 nm using an extinction coefficient of 34 950 M^−1^ cm^−1^, which was calculated from the amino acid sequence.

### Oligonucleotides

All oligonucleotides used in this study were purchased HPLC-purified from Integrated DNA Technologies (Coralville IA). The 83-mer oligonucleotides, originally used by Karakousis *et al.* (12), were taken from M13 plus strand (position 265 to 183) or its complement. The sequences of the 50-mer oligonucleotides used for the gel-based annealing assay were taken from Subramaniam *et al.* (26). Sequences of all oligonucleotides used in this study are given in Supplementary Figure S3.

### DNA annealing assay

A gel-based annealing assay employing complementary Cy5- and Cy3-labeled 50-mer oligonucleotides was performed as originally described by Subramaniam *et al.* (26). Redβ-ssDNA complexes were prepared by incubating 10 μM Redβ with 50 μM (nucleotides) of one 50-mer oligonucleotide (Cy5 50mer) for 30 min at 37°C in one of three different buffers: PBS, Mg^2+^-containing buffer (20 mM Tris-HCl, pH 7.5, 10 mM NaCl, 10 mM MgCl_2_), or 50 mM ammonium acetate pH 7.0. If indicated, 50 μM of the complementary oligo (Cy3 50mer) was then added to the reaction and incubated for an additional 30 min to form the complex with annealed duplex. For some experiments (as indicated) the Cy3 50mer oligo was added first. Samples were mixed with 10× Orange G loading dye (65% (w/v) sucrose, 10 mM Tris-HCl pH 7.5, 10 mM EDTA, 0.3% (w/v) Orange G), loaded onto a 1.0% agarose gel in 2× TBE buffer, and electrophoresed at 90 V for 40 min. Gels were imaged on an Azure Biosystems Sapphire with channels selected to capture fluorescent signals from the Cy3 and Cy5 probes. Additional experiments with Cy3 and Cy5 labeled 16, 20, 24 and 28mer oligonucleotides were performed in the same manner in PBS.

### SEC-MALS

SEC-MALS experiments were performed with a Superose 6 increase 3.2/300 column (GE Healthcare) connected to an inline Wyatt miniDAWN TREOS system with PBS as the running buffer and a flow rate of 0.04 ml/min. The column, which has a separation range of 5–5000 kDa, was calibrated with five high molecular weight protein standards (GE Healthcare; Supplementary Figure S4). To establish the accuracy of this setup, the MALS masses of four protein standards were measured, and found to be within 5–16% of their calculated molecular weights (Supplementary Figure S4A). All SEC-MALS measurements of Redβ were performed in triplicate, and the values for the resulting parameters including elution volume (*V*_e_), MALS mass and number of Redβ subunits (*n*) are reported In Table 1.

**Table 1.** SEC-MALS data

		SEC			SEC-MALS		
Sample [μM loaded]	*V*_e_ (ml)	*M* (kDa)	(*n*)	*C* (μM)	*M* (kDa)	(*n*)	nt or bp /mon
Redβ [34]	1.47	640 ± 30	21.6	6.0 ± 0.4	228 ± 3	7.7	
Redβ [67]	1.45	740 ± 60	24.8	13.5 ± 0.7	256 ± 1	8.6	
Redβ [101]	1.45	750 ± 30	25.3	18.5 ± 0.4	270 ± 4	9.1	
Redβ + dT38 [34]	1.37	1170 ± 80	39.0	10.5 ± 8.9	427 ± 10	14.0	2.7
Redβ + 83- [34]	1.33	1580 ± 120	52.2	10.5 ± 5.6	653 ± 23	21.1	3.9
Redβ + dT38:dA38 [34]	1.35	1310 ± 110	38.5	5.1 ± 0.9	358 ± 8	11.3	3.4
Redβ + 83-:83+ [34]	1.29	2120 ± 80	69.8	3.2 ± 0.2	591 ± 13	18.2	4.6

Values for the mass (*M*) determined from the SEC elution volume (*V*_e_) or MALS signal, and the concentration (*C*) determined from the A280 value at elution, report the mean and standard deviation from three independent experiments. (*n*) is the number of subunits of Redβ determined from each mean *M* value, after subtracting out the mass of a single copy of each ssDNA or annealed duplex. The column labeled ‘nt or bp/mon’ gives the observed stoichiometry of each complex in nucleotides or base pairs per monomer of Redβ. The extremely large masses determined from the *V_e_* values presumably reflect the nonspherical nature of the complexes. The void volume of the column, as measured with blue dextran, was 0.96 ml. The calibration curve for the MW standards, used to determine *M* from *V*_e_, is shown in Supplementary Figure S4B.

Samples were loaded in volumes of 50 or 100 μl at 1.0–3.0 mg/ml for Redβ alone, and 1 mg/ml for all DNA complexes. Redβ–ssDNA complexes were prepared by adding an oligonucleotide, either dT38 or 83-, at 1.5-fold excess over Redβ (six nucleotides/monomer), and incubating at 22°C for 30 min. The complex with annealed duplex was prepared by first forming the Redβ–ssDNA complex with a stoichiometric amount (4 nt per monomer) of the first oligonucleotide (dT38 or 83-), and then adding the complementary oligonucleotide (dA38 or 83+), and incubating for an additional 30 min (at 22°C). Protein elution was monitored by absorbance at 280 nm (A280) and MALS signal, which was converted into molecular weight with ASTRA software using a Debye model. The concentration of each protein or protein–DNA complex used for this calculation was determined from the A280 value at each elution point, and extinction coefficients (in units of A280 per 1 mg/ml) calculated from the protein and DNA sequences. For the complexes, extinction coefficients were determined assuming additivity (35), using the Redβ monomer to DNA ratio observed for each complex (14:1 for the complex with dT38, 11:1 for the complex with dT38:dA38, 21:1 for the complex with 83-, and 18:1 for the complex with 83-:83+). The extinction coefficients for each individual species were 1.18 for Redβ, 18.7 for dT38, 11.8 for dT38:dA38, 19.5 for 83- and 15.7 for 83-:83+. For the oligonucleotide sequences, these values were obtained by converting the extinction coefficient calculated from the sequence at 260–280 nm using measured 260/280 ratios of 1.43 for dT38, 2.00 for dT38:dA38, 1.64 for 83- and 1.65 for 83-:83+. The resulting extinction coefficients used for the complexes were 1.72 for Redβ with dT38, 1.80 with dT38:dA38, 1.90 with 83-, and 2.31 with 83-:83+. Experiments for each sample were performed in triplicate to obtain a mean and standard deviation for each mass measurement, as reported in Table 1.

### AUC

Sedimentation velocity (SV) experiments were performed using a Beckman Coulter ProeomeLab XL-I analytical ultracentrifuge with an 8-position An50Ti rotor (Beckman Instruments, Inc., Fullerton, CA). Redβ protein alone was prepared from frozen stock by dialysis into PBS at 4°C and diluting with dialysate to experimental concentrations. Redβ complexes with ssDNA substrate (dT38) and annealed duplex product (dT38:dA38) were prepared in 500 μl volumes with 20 mg/mL Redβ (diluted from 48 mg/ml frozen stock), and the same method and relative amounts of each oligonucleotide described above for SEC-MALS. To separate out excess unbound DNA, each DNA complex was purified using a HiPrep 16/60 Sephacryl S-300 column (GE Healthcare) with PBS as running buffer at 0.5 ml/min collecting 2 ml fractions. The fraction centered on the peak for each complex was analyzed by A260/A280 to determine the relative concentrations of DNA and protein, and diluted in column running buffer (PBS) to experimental concentrations. Samples for SV experiments were loaded at 425 μl volumes into double-sector charcoal filled epon centerpieces, at concentrations of 0.25, 0.50 and 1.0 mg/ml, using PBS column buffer or dialysate as the reference buffer. After an initial equilibration for >1 h at 20°C to prevent convection, samples were centrifuged at 20 000 rpm at 20°C for 7 h. Scans for A280 and fringe interference (FI) were collected every 7 min for 7 h (60 scans total). Sedimentation coefficients and fitted masses for selected individual peaks were determined using the continuous distribution *c*(*s*) model in SEDFIT version 16.1c (36), and plotted in GUSSI (37). Apparent sedimentation coefficient distributions (*g*(s*)) and fitted masses were calculated using DCDT+ version 2.5.1 (38,39). Solvent properties of PBS at 20°C (viscosity = 0.01012 g/cm^3^, density = 1.00566 g/ml) and the partial specific volumes (PSV) of Redβ (0.728 ml/g), dT38 (0.55 ml/g), and dA38 (0.55 ml/g) were determined using ULTRASCAN III (40). The PSVs of the Redβ-dT38 (0.7214 ml/g) and Redβ-dT38:dA38 (0.7151 ml/g) complexes were determined assuming additivity (35) and 10 moles of Redβ (MW = 29 970.1 g/mol) per mole of dT38 ssDNA (MW = 11 497.4 g/mol) or dT38:dA38 annealed duplex (MW = 23 337.3 g/mol).

### nMS

Native MS experiments were performed on a Q Exactive Ultra-High Mass Range (UHMR) mass spectrometer from Thermo Fisher (41,42) that was modified to allow for surface-induce dissociation (SID, not used in this work) similar to a previously described modification (43). Redβ protein was prepared by buffer exchange into 50 mM ammonium acetate pH 7 (unadjusted) using Micro Bio-Spin P6 spin columns (Bio-Rad Laboratories, Hercules, CA, USA). All ssDNAs were dialyzed into 50 mM ammonium acetate with a Pierce 96-well microdialysis plate, 3.5K MWCO (Thermo Fischer). Quality of oligonucleotides was also checked by nMS using a rapid online buffer exchange protocol described previously (44). For preparation of Redβ-DNA complexes, Redβ was diluted to the experimental concentration indicated, and then the first ssDNA was added at a concentration ratio of 4 nt per monomer of Redβ and incubated at room temperature for at least 15 min. For complexes with annealed duplex, the second ssDNA was then added and incubated for an additional 15 min. Each sample was then injected into an in-house pulled borosilicate filament capillary (OD 1.0 mm, ID 0.78 mm) and subsequently ionized by nano-electrospray ionization. The following instrument tuning settings were kept constant for all samples: capillary temperature 250°C, Source DC Offset 21 V, S-lens RF level 200, detector *m/z* optimization low *m/z*, noise threshold 4.64, ion transfer target high *m/z*, Injection flatapole DC 5 V, inter flatapole lens 4 V, bent flatapole DC 2 V, Transfer multipole DC 0 V, C-trap entrance lens inject 1.8 V, HCD field gradient 200 V, HCD cell pressure 5 (UHV Sensor ∼3 - 4E-10 mbar), and resolution 6250 as defined at 400 *m/z*. Spray voltage was adjusted between 0.6 and 0.8 kV and then held constant for the duration of the acquisition. Ion activation was necessary for improved transmission and de-adducting of ions to resolve species at higher *m/z*. For this, either activation via in-source trapping (IST) of -60 V or higher energy collisional dissociation (HCD) of 60 V was used.

All data were deconvolved using UniDec V4.1.0 – 4.2.1 (45). A range of deconvolution settings were tested initially and then optimized in an effort to best include all species present. However, due to the lower apparent resolution at higher *m/z*, the peak FWHM had to be balanced between over fitting the high *m/z* data and under fitting at low *m/z*. This balance did result most notably in a poorer fit at *m/z* less than ∼4000. The deconvolution settings used for Redβ alone include the following: *m/z* range 1000–12000, charge range 1–70, mass range 5–500 kDa, sample mass every 5 or 10 Da, split Gaussian/Lorentzian peak FWHM 5 or 10 Th, charge smooth width 1.0, manual mode was applied for the +10 monomer and the +15 dimer. Settings for Redβ plus 38-mer DNA were adjusted to a charge range of 1–60, sample mass every 5 Da, split Gaussian/Lorentzian peak FWHM 5 Th, and manual mode was set to include the +10 Redβ monomer, +5 dA38, and the +8 dT38:dA38. Settings for Redβ plus 83:87-mer DNA were adjusted to 2000–15000 *m/z*, charge range of 1–100, mass range of 10–1000 kDa, and manual mode was set to include the +10 Redβ monomer, the +7, +8, and +9 monomer DNA, and the +12 and +13 dimer DNA. The resulting deconvolutions were plotted as relative signal intensities. In an effort to maintain consistency across the spectra analyzed, these settings were used throughout although occasionally small changes were made that included adjusting the *m/z* range or adjusting the manually assigned species.

## RESULTS

### Measuring the concentration of Redβ expressed in cells active for recombination

The *in vivo* concentration of Redβ expressed in two systems commonly used for recombineering was measured by quantitative western blot. The first used a pSIM5 plasmid to express the λ red functions (Redβ, λ exo, and gam) from their native P_L_ operon under control of a temperature sensitive λ repressor, induced by shifting from 32 to 42°C (29). pSIM5 was transformed into HME57 cells deficient in mismatch repair, which have been reported to yield 1.8 × 10^7^ recombinants per 10^8^ viable cells in single stranded oligo repair (ssOR) experiments (29). The second system used a pSC101-based plasmid (hereafter referred to as pSC101) to express the λ red functions from an arabinose-inducible P_BAD_ promoter (30). The pSC101 plasmid was transformed into GB2005 cells containing a bacterial artificial chromosome encoding a defective neomycin gene. ssOR experiments using this system have reported 8.6 × 10^6^ recombinants per 2.1 × 10^8^ viable cells (30), lower than for pSIM5, but with some advantages including tightly regulated expression and the ability to cure the cells of the plasmid *via* temperature-sensitive replication.

To determine the *in vivo* concentration of Redβ expressed from pSIM5 and pSC101 in the cells used for their respective ssOR experiments, quantitative western blots were performed to measure the amount of Redβ present in 10–16 μl of soluble cell lysate, by comparing to lanes containing known amounts of purified Redβ protein (Figure 2; Supplementary Figure S1 and Supplementary Table S1). From this value, the amount of Redβ per cell was determined by dividing by the number of cells used to prepare the loaded volume of lysate, as determined from the OD_600_ of the original culture just prior to harvesting. This calculation assumed that an OD_600_ of 1.0 equates to 8.0 × 10^8^ cells/ml (25). Finally, the concentration of Redβ in cells was obtained by dividing by the volume of a single *E. coli* cell, which was taken to be 3.8 × 10^−9^ μl (25,34). Based on at least three independent experiments for each plasmid, the *in vivo* concentrations of Redβ expressed from pSIM5 and pSC101 were determined to be 27 ± 12 and 7 ± 2 μM, respectively, which equates to 0.81 and 0.21 mg/ml.

### DNA annealing assay and buffer considerations

A gel-based DNA annealing assay was performed to confirm the activity of the purified Redβ protein used in this study (Supplementary Figure S5). This assay, originally described by Subramaniam *et al.* (26), monitors the binding of Redβ to two complementary 50-mer oligonucleotides labeled at the 5′-end with either Cy5 or Cy3. For reasons explained below, this annealing assay was performed in three different buffers: PBS, a Mg^2+^-containing buffer (detailed in ‘Materials and Methods’ section), and 50 mM ammonium acetate. Very similar annealing activity is seen with all three buffers. When Redβ is incubated with either oligonucleotide individually, weak if any complex formation is observed (Supplementary Figure S5, lanes labeled SS). If the two complementary oligonucleotides are added to Redβ sequentially however, a tight complex is formed, as seen by the prominent shifted band of yellow color, indicating that both strands are present (lanes labeled AD for annealed duplex). This was further confirmed by the single-channel exposures of Supplementary Figures S5B and S5C, which show signal for only one oligonucleotide or the other. As originally described by Karakousis *et al.* (12), this complex presumably contains Redβ bound tightly to a duplex intermediate of annealing. The same amount of complex is formed regardless of which oligonucleotide is added to Redβ first (lanes labeled AD Cy3 and AD Cy5). If the two complementary strands are pre-annealed before adding Redβ, no complex is formed (lanes labeled DS), indicating that the tight binding of Redβ is inherent to the annealing reaction itself. Finally, as originally demonstrated by Karakousis *et al.* (12), formation of this complex is fully dependent on the two sequentially added oligonucleotides being complementary to one another. If the second oligonucleotide is noncomplementary to the first, then the complex that is formed contains only the first of the two strands that was added (lanes labeled NC Cy3 and NC Cy5). As each oligonucleotide was added in slight excess over Redβ in this experiment (5 nt/monomer), this latter result suggests that under these conditions (10 μM Redβ and 15 min at 37°C) Redβ remains bound to the first oligonucleotide added and does not exchange onto the second. Curiously, Redβ has unusually high affinity for the ‘Cy3 NC 50mer’ oligonucleotide (Supplementary Figure S5, lane NC Cy3), but still does not transfer onto it if the noncomplementary oligonucleotide (‘Cy5 50mer’) is added first (lane NC Cy5).

Subramaniam *et al.* performed a very similar gel-based annealing assay in a low ionic strength buffer containing 10 mM Mg^2+^ (26). Early *in vitro* studies of Redβ indicated that Mg^2+^ is required for annealing activity (5,6) and oligomer formation (13). However, Karakousis *et al.* (12) and more recently our group (27,28) have demonstrated Redβ annealing activity in the absence of Mg^2+^. As PBS is widely considered to be a good mimic of physiological ionic strength, we chose PBS for the biophysical studies presented below (SEC-MALS and AUC), and this choice is validated by the robust activity observed in PBS in the annealing assay described above. The nMS method however requires a buffer with volatile components, and ammonium acetate is a common choice. Importantly, the annealing assay of Supplementary Figure S5 also demonstrates that Redβ is fully active in the 50 mM ammonium acetate we used for nMS. Finally, using a similar Mg^2+^-containing buffer as Subramaniam *et al.* (26), Erler *et al.* (16) previously demonstrated that the minimum oligonucleotide length required for robust annealing *in vitro* is 20 nucleotides. Using PBS and sets of complementary oligonucleotides ranging from 16 to 50 nucleotides in length, the annealing assay of Supplementary Figure S6 gives a strikingly similar result: robust annealing for oligonucleotides of length 20 or greater, and only faint complex formation with 16-mers. This result further establishes the validity of using PBS for our biophysical measurements, and of comparisons with previous studies performed in Mg^2+^-containing buffers (16,26). Supplementary Figure S6 also shows weak binding to 50-mer ssDNA, but no binding to ssDNA that is 28 nt or shorter, consistent with prior observations that Redβ can bind to 36-mer ssDNA but not to 27-mer (46).

### SEC-MALS

**Figure 3.**
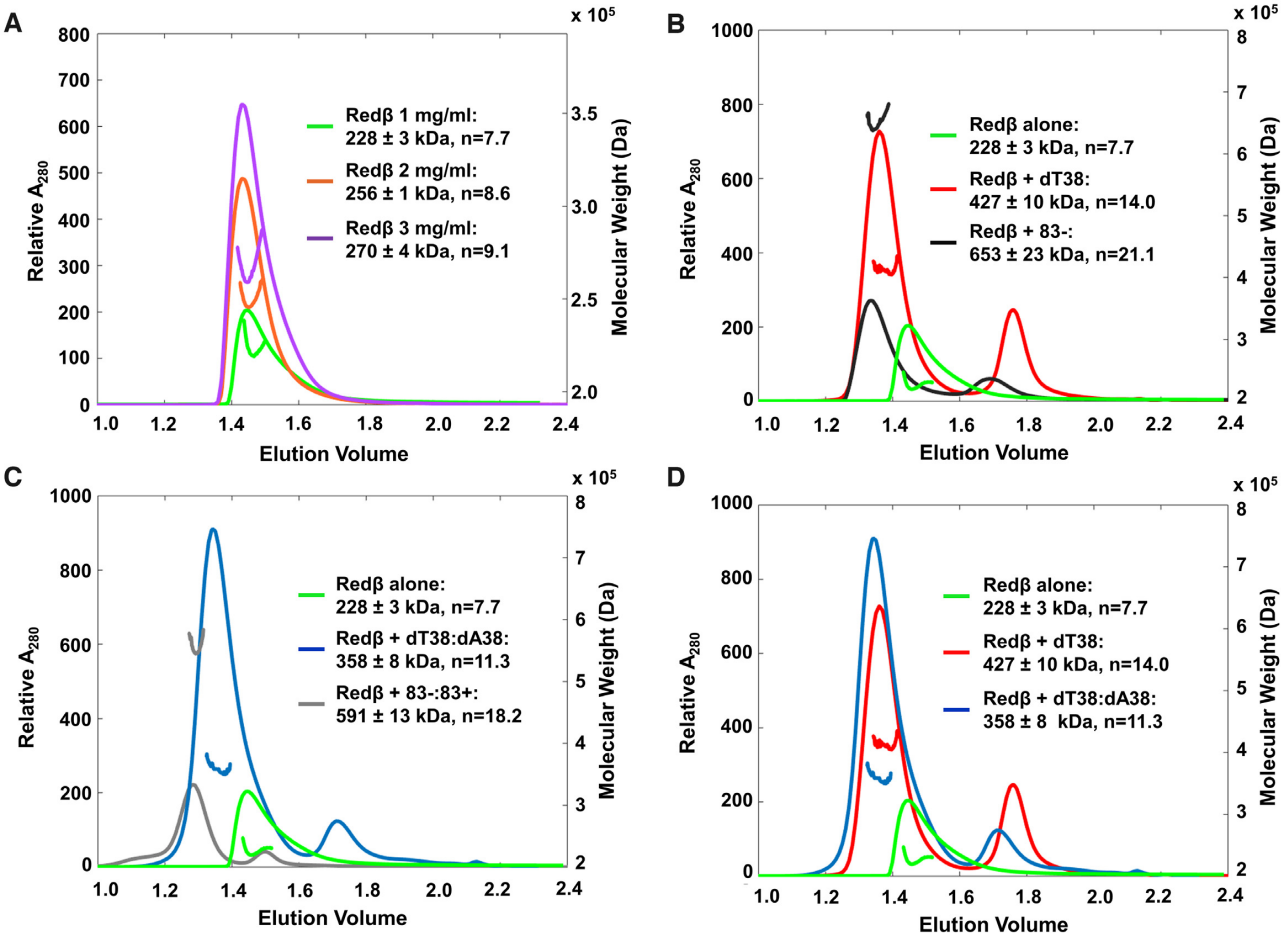
SEC-MALS data. (**A**) Redβ alone loaded at 1, 2 and 3 mg/ml reveals a small but reproducible increase in oligomer mass at higher concentration. Subsequent experiments with DNA (panels B–D) were performed at 1 mg/ml. The axis to the right of each plot gives the mass calculated from the MALS signal, as indicated by the horizontal line under or over the major peak for each trace. (**B**) Comparison of Redβ alone and in complex with dT38 or 83- ssDNA. The small downstream peaks contain excess unbound ssDNA. For the complex with 83-, only 50 μl was loaded (instead of 100 μl), as the 83- was in limited supply. (**C**) Comparison of Redβ alone and in complex with annealed duplexes formed by dT38:dA38 or 83-:83+. The small downstream peaks contain excess unbound DNA. For the complex with 83-:83+, only 50 μl was loaded (instead of 100 μl), as the 83- and 83+ oligos were limited. (**D**) Overlay comparing Redβ alone and its complexes with dT38 ssDNA and dT38:dA38 annealed duplex. The green trace for Redβ alone at 1 mg/ml is the same in each plot, for reference.

We first measured the mass of the oligomer formed by Redβ in the absence of DNA (Figure 3A). Redβ loaded onto the column at 1.0 mg/ml gave a detectable MALS signal, but lower concentrations including 0.1 and 0.01 mg/ml did not (data not shown). Therefore, 1 mg/ml (34 μM) was chosen as the lowest concentration for analysis. Note that the protein is diluted as it runs down the column, and Redβ loaded at 34 μM eluted at a concentration of 6.0 ± 0.4 μM as determined by A280 (Table 1). At this concentration, Redβ elutes as a single peak with a MALS mass of 228 ± 3 kDa, which corresponds to an oligomer of 7.7 subunits. The peak has a tail extending rightward toward smaller species, which is likely due to transient dissociation of the oligomer as it runs down the column, but could also arise from weak interaction of the protein with the column matrix. To assess the effect of protein concentration, samples of Redβ were also loaded at 2 and 3 mg/ml (67 and 101 μM), which eluted at concentrations of 13.5 ± 0.7 and 18.5 ± 0.4 μM, respectively. The size of the oligomer increased slightly but reproducibly over this range, to 256 ± 1 kDa at 13.5 μM (8.6 subunits), and 270 ± 4 kDa at 18.5 μM (9.1 subunits). This increase in mass could be due to formation of a larger oligomer (or distribution of oligomers), or to less dissociation of monomers from a distinct but larger oligomer such as a 12-mer. Support for the latter possibility comes from prior analysis of Redβ by dynamic light scattering (DLS), which detected a mixture of 32% oligomer (11- or 12-mer) and 68% monomer present in a peak for oligomer purified by gel-filtration (26). Our measurement was by static light scattering, which is not able to resolve species in rapid equilibrium, and instead gives a weight-average of all species present (47). Collectively, the data are consistent with Redβ forming a larger oligomer such as a 12-mer that is in rapid equilibrium with monomers.

We next measured the mass of the oligomers formed by Redβ in the presence of ssDNA substrate (Figure 3B). Based on the detectable concentration of Redβ established above, 1 mg/ml (34 μM) was chosen as the loading concentration for analysis of DNA complexes. This resulted in concentrations of 3–11 μM at elution (Table 1), which overlaps with our measured *in vivo* concentrations of 7–27 μM (Figure 2). A dT38 oligonucleotide was chosen as the first ssDNA substrate to examine, as this is the approximate length of ssDNA that could fit around an 11-mer ring with a stoichiometry of 4 nt/monomer, by analogy with Rad52 (20,48). It is also slightly larger than the minimal length of ssDNA required for Redβ binding *in vitro* (Supplementary Figure S6 and reference 46), and for the regions of complementarity required for successful recombination *in vivo* (9,10). The presumed 4 nt/monomer stoichiometry is close to the 4–5 nt/monomer measured by titration with M13 ssDNA *in vitro* (46). To prepare the Redβ-dT38 complex (and to ensure full complex formation), a 1.5-fold excess of dT38 (6 nt/monomer) was added to Redβ in PBS and incubated at 22°C for 30 min. As expected, a downstream peak for excess unbound dT38 was observed (Figure 3B, red trace), indicating that the peak for the oligomer contained a fully saturated Redβ-dT38 complex. This peak eluted with a MALS mass of 427 ± 10 kDa, which, assuming a single copy of the dT38 oligonucleotide, would correspond to an oligomer with 14.0 subunits of Redβ. This is almost twice as large as the oligomer formed by Redβ alone at this concentration (7.7 subunits). Such an increase in size could arise if the ssDNA stabilizes the oligomer to prevent dissociation of subunits, if the oligomer on ssDNA is larger than that for the free protein, or if two Redβ oligomers bind to a single dT38 ssDNA. Although the SEC-MALS data do not distinguish among these possibilities, data from EM (13) and from nMS presented below provide support for the first two possibilities.

To assess the effect of ssDNA length, a complex of Redβ with a significantly longer oligonucleotide, an 83-mer with a naturally occurring sequence that has been used in previous studies (12) was examined. This is similar to the length of oligonucleotides commonly used for ssOR experiments *in vivo* (29,30). This complex, which was also prepared with a 1.5-fold excess of 83-mer over Redβ, elutes with a MALS mass of 653 ± 23 kDa, which, assuming a single copy of the 83-mer ssDNA, would correspond to an oligomer with 21.1 subunits of Redβ. Although this complex is significantly larger than that with dT38, it is not twice as large, as one might expect from the 83-mer being just over twice as long as the 38-mer. Consequently, the apparent stoichiometries of the two complexes are somewhat different: 2.7 nt/monomer for dT38, and 3.9 nt/monomer for the 83-mer. Whether or not the full-length of the ssDNA is coated by monomers of Redβ in each complex cannot be determined from the data. However, as downstream peaks for excess ssDNA were present for both samples (red and black curves in Figure 3B), both complexes should have been fully saturated.

Next, we measured the mass of the complex of Redβ bound to the annealed duplex formed when two complementary oligonucleotides are added to the protein sequentially (Figure 3C). To prepare this complex with 38-mer oligonucleotides, Redβ was first incubated with a stoichiometric amount (4 nt/monomer) of dT38 at 22°C for 30 min, and then an equivalent amount of dA38 was added and incubated for an additional 30 min. Again, a downstream peak for excess unbound DNA was observed, indicating that the peak for the complex contained a saturating amount of the annealed duplex. The complex elutes with a MALS mass of 358 ± 8 kDa, which, assuming a single copy of dT38:dA38, would correspond to an oligomer with 11.3 subunits of Redβ. This is about three subunits fewer than the complex with dT38 alone, suggesting that as the complementary dA38 is added, the complex may actually lose about three subunits of Redβ.

To assess the effect of DNA length, the complex with annealed duplex was also prepared with two complementary 83-mer oligonucleotides. This complex elutes with a MALS mass of 591 ± 13 kDa, which, assuming one copy of the 83-mer annealed duplex, would correspond to an oligomer with 18.2 subunits of Redβ. Again, this is significantly larger than the complex with two 38-mers, but not twice as large, such that the apparent stoichiometry changes from 3.4 bp/monomer for complex with 38-mers, to 4.6 bp/monomer for the complex with 83-mers. Moreover, the 18.2 subunits for the complex with two 83-mers is three subunits fewer than the complex with just one 83-mer (21.1 subunits), again suggesting that as the second strand of complementary ssDNA enters the complex, about three subunits of Redβ are displaced.

In summary, the SEC-MALS data indicate that in the absence of DNA, Redβ forms an oligomer ranging from 7.7 to 9.1 subunits, depending on the concentration, but this is likely an underestimate due to rapid dissociation of a larger oligomer into monomers. Addition of the dT38 or 83-mer ssDNA increased the size of the oligomers to 14.1 and 21.1 subunits of Redβ, respectively, with stoichiometries of 2.7 and 3.9 nt/monomer. Whether the increased size on the longer ssDNA is due to the binding of additional copies of a ‘unit’ oligomer (such as a 12-mer), or to the expansion of a single, continuous oligomer on the ssDNA, cannot be discerned from the data. Addition of a second ssDNA that is complementary to the first, dA38 or 83+, to form the complex with annealed duplex, reduced the apparent size of the oligomer to 11.3 and 18.2 subunits, respectively, with stoichiometries of 3.4 and 4.6 bp/monomer. For both types of complexes, with ssDNA substrate and with the annealed duplex intermediate, the size of the oligomer increased with DNA length, but not linearly: a DNA that is twice as long did not harbor twice as many subunits.

### Sedimentation velocity (SV)

To assess the sizes of the Redβ oligomers formed by a biophysical method that is complementary to SEC-MALS, we turned to AUC by sedimentation velocity (SV). DNA complexes with dT38, or dT38 and dA38 added sequentially were prepared in PBS as described above for SEC-MALS, except at higher concentrations (20 mg/ml Redβ, diluted from 48 mg/ml stock) for purification by gel-filtration, to remove excess DNA and ensure that the complexes were fully saturated. Peak fractions from the column, which contained Redβ at 2–3 mg/ml, were further diluted to experimental concentrations in PBS column running buffer, which was used as reference. All three states were analyzed at concentrations of approximately 8, 17 and 34 μM Redβ (0.25, 0.5 and 1.0 mg/ml) to give nine samples total. Each sample was analyzed using both the A280 and FI optics, to give 17 data sets total (the FI data for Redβ 0.5 mg/ml were not interpretable).

**Figure 4.**
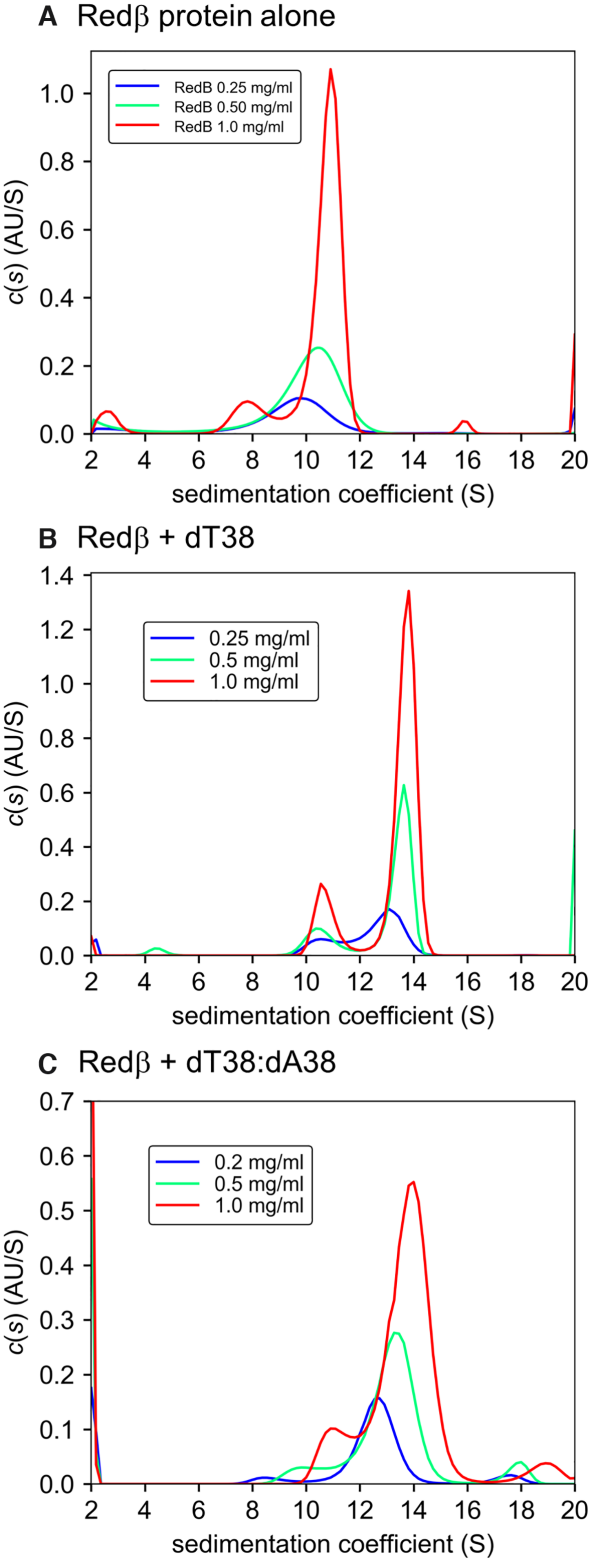
*c(s)* distributions from sedimentation velocity data. A280 data from runs at three different concentrations are overlaid for (**A**) Redβ protein alone, (**B**) Redβ + dT38, and (**C**) Redβ + dT38:dA38 annealed duplex. Complexes with DNA were purified by gel filtration to remove excess DNA and to ensure that they were saturated. The distributions were calculated with SEDFIT (36) and plotted with GUSSI (37). Comparisons of the A280 and FI data for each sample are presented in Supplementary Figure S10.

Each data set was fit to the continuous distribution *c*(*s*) model in SEDFIT (36), and the raw data for the fits are shown in Supplementary Figures S7 and S8 for the A280 and FI data, respectively. The *c*(*s*) distributions from the A280 data are shown in Figure 4, and the resulting parameters for the predominant species of each sample are summarized in Table 2, for both the A280 and the FI data. Additional overlays of the *c*(*s*) distributions are shown in Supplementary Figure S9, which compares the three states at each concentration, and in Supplementary Figure S10, which compares the distributions from the A280 and FI data for each sample. The number of subunits (*n*) for each DNA complex was calculated from the fitted mass, assuming one copy of the dT38 oligonucleotide for the ssDNA complex, or one copy of dT38:dA38 for the complex with annealed duplex.

**Table 2.** Sedimentation velocity *c*(*s*) data

	A280						FI					
Sample [μM]	*Sw,20*	*M/*min	(*n*)	*f/f0*	*SR*	rmsd	*Sw,20*	*M/*min	(*n*)	*f/f0*	*SR*	rmsd
Redβ [8.3]	9.9	279/141	9.2	1.6	6.8	0.0038	10.1	322/146	10.7	1.7	7.7	0.0031
Redβ [16.6]	10.3	285/151	9.5	1.5	6.6	0.0070	—	—		—	—	—
Redβ [34]	11.1	322/169	10.7	1.5	7.0	0.0070	11.0	390/166	13.0	1.8	8.5	0.0056
Redβ + dT38 [8.3]	13.3	376/212	12.5	1.5	7.0	0.0036	12.3	372/189	12.4	1.6	7.4	0.0020
Redβ + dT38 [16.6]	13.9	360/228	12.0	1.4	6.4	0.0048	13.0	425/206	14.2	1.6	8.0	0.0041
Redβ + dT38 [34]	14.1	392/232	13.1	1.4	6.8	0.0063	13.9	466/226	15.5	1.6	8.3	0.0047
Redβ + dT38:dA38 [8.3]	12.9	346/196	11.5	1.5	6.7	0.0028	12.6	386/188	12.9	1.6	7.7	0.0022
Redβ + dT38:dA38 [16.6]	13.5	370/210	12.3	1.5	6.9	0.0035	13.0	422/198	14.1	1.7	8.2	0.0027
Redβ + dT38:dA38 [34]	14.2	362/225	12.1	1.4	6.4	0.0061	13.7	447/215	14.9	1.6	8.2	0.0034

The entries for each sample show the values from consensus fits to the *c(s)* model in SEDFIT (36) using either the A280 (left) or the FI data (right). The *M/*min column gives the mass (*M*) in kDa from the fit of each peak in the distribution, with corresponding frictional ratio (*f/fo*), and the minimum mass (*min*) that would correspond to a spherical particle with *f/fo* of 1. This latter value (min), which is routinely output by SEDFIT, is given here to provide comparisons independent of any uncertainties in the fitted frictional ratio. (*n*) is the number of subunits of Redβ (29.97 kDa for the purified protein with N-terminal GSH) that would be present in an oligomer of the observed mass, assuming one copy of dT38 ssDNA or dT38:dA38 annealed duplex. *SR* is the Stokes radius from the fit, in nanometers.

Overall, the results from the *c*(*s*) analysis indicate that Redβ forms an oligomer (or range of oligomers) of 9–13 subunits in the absence of DNA, 12–16 subunits in the complex with dT38, and 12–15 subunits in the complex with the dT38:dA38 annealed duplex. Thus, as suggested by the SEC-MALS data, the complex appears to pick up additional subunits of Redβ when the first ssDNA is added. However, the complex with annealed duplex appeared more similar to that with ssDNA: the apparent loss of ∼3 subunits upon addition of the complementary ssDNA observed by SEC-MALS was not as evident from the *c(s)* analysis.

In all three states, a slight but consistent dependence on sample concentration is apparent, with a trend toward larger Redβ oligomers at higher concentrations (Table 2). Because the *c*(*s*) model assumes a non-interacting system with all species having the same frictional ratio (*f/fo*), we also analyzed the data with the *g*(s*) model (38,39), which makes fewer assumptions, albeit with lower resolution (Supplementary Figure S11 and Supplementary Table S2). In general, the results and conclusions from the *g*(s*) analysis are similar to those from *c*(*s*): the Redβ oligomer has 8–10 subunits in the absence of DNA, 10–11 subunits with dT38 and 8–9 subunits with dT38:dA38. The *g*(s*) analysis tends to give slightly smaller masses, and correspondingly lower values for (*n*) in all three states. A slight concentration dependence is observed, particularly for the oligomer in the absence of DNA (Supplementary Figure S11A). In addition, the apparent loss of subunits upon addition of the complementary ssDNA that was seen by SEC-MALS, but not by the *c(s)* analysis, is somewhat evident from the g(s*) analysis.

In the *c*(*s*) model, the A280 and FI data give very similar *S* values, but the fits to the FI data result in somewhat higher frictional ratios (1.6–1.8 for FI compared to 1.4–1.6 for A280), and correspondingly higher masses. One difference between the two detection methods is that the contribution from the DNA is considerably higher for the A280 data than for the FI data. Along these lines, the samples containing DNA at the highest concentration (1 mg/ml) may have been slightly above the linear range of the A280, as shown in Supplementary Figure S7 (AU signal approaching 1.4). However, the same discrepancy of frictional ratio was also seen at the two lower concentrations. We are unclear on exactly how these factors could result in larger frictional ratio for the FI data. In any case, while the masses from the FI data are slightly higher, the corresponding number of subunits changes only slightly, and the overall conclusions from the data are similar.

Finally, some of the distributions can show additional minor species, depending on the degree of regularization, although the regularization parameter was always fixed (at *P* = 0.683). A prominent secondary species is a peak at *S(w,20)* of around 10–11 that is most prominent for the complex with dT38 (Figure 4B). This could conceivably be due to a small portion of un-complexed Redβ oligomer, or to the presence of a proteolytically cleaved fragment of Redβ such as the N-terminal DNA binding domain. Although we did not observe any degradation products of Redβ by SDS-PAGE (Supplementary Figure S2) or nMS (Supplementary Figure S13), proteolysis could have conceivably occurred during the long duration of the AUC experiment at 20°C. Support for this second explanation is evident from the distribution of Redβ protein alone at the highest concentration (1.0 mg/ml), which shows a similar peak, but at lower *S(w,20)* of around 7–8, as there is no DNA (Figure 4A). Moreover, the relative height of this peak is similar in the A280 and FI data (Supplementary Figure S10F) suggesting that it contains DNA; if it had no DNA a lower relative signal for A280 would be expected. A few additional peaks that could correspond to even smaller fragments or higher-ordered oligomers are present in some of the samples, but overall less prevalent.

### Native mass spectrometry (nMS)

Finally, we turned to nMS as a complementary and significantly higher resolution method to more accurately determine the exact oligomeric species of Redβ that are present in each of the three states. As this method ideally requires volatile buffer components, measurement in PBS was not possible. Instead, the protein (or DNA) was buffer exchanged (or dialyzed) into 50 mM ammonium acetate (pH ∼ 7, unadjusted), and after dilution of the protein and DNA individually, the components were mixed at the final concentrations indicated. An exception to this was the 30 μM samples containing Redβ or Redβ + 38-mer DNAs (dT38 and dA38), which were mixed at 30 μM in PBS for complex formation, and then buffer exchanged into 50 mM ammonium acetate. However, this did not appear to affect the outcome as essentially the same oligomer species were observed at lower concentrations.

**Figure 5.**
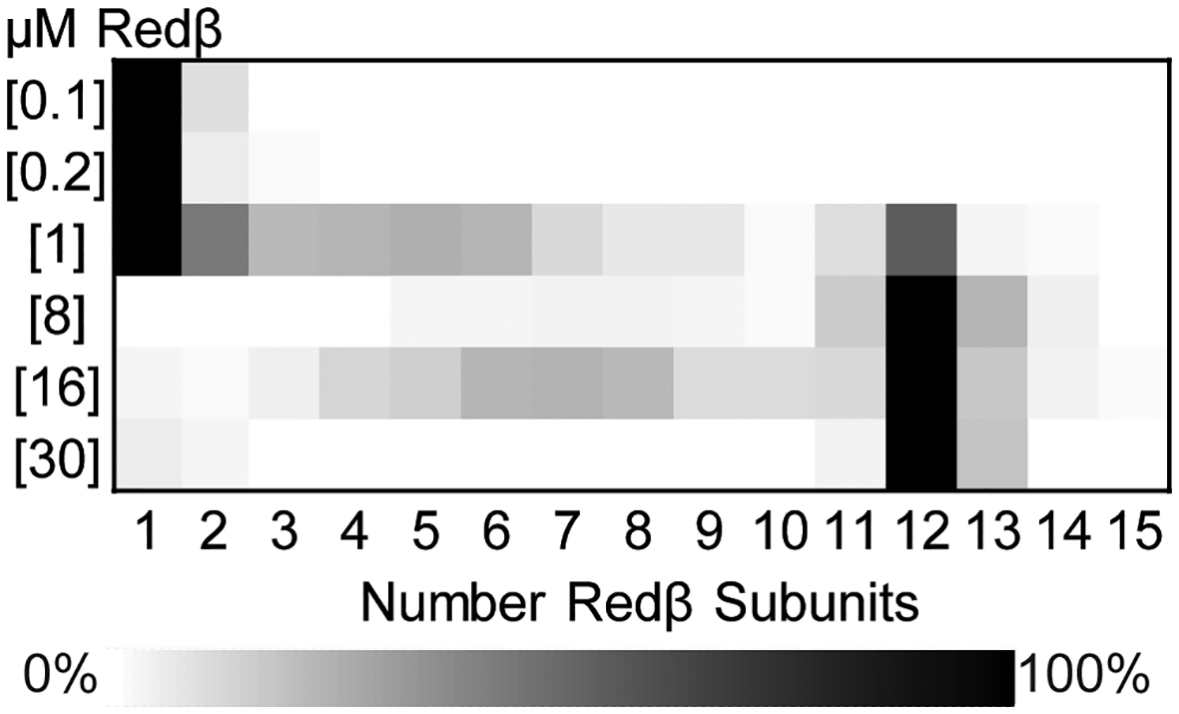
Native MS heat map displaying the relative intensities of the different oligomer species of Redβ as a function of concentration. The relative intensities were determined from the deconvolved spectra shown in Supplementary Figure S13. In all cases a collision voltage of 60 was used.

First, the masses of all individual components were measured, to validate their MWs (Supplementary Tables S3 and S4, Supplementary Figures S12 and S13). The correct masses of all components were confirmed, and no significant impurities were detected. We then collected mass spectra for Redβ alone at concentrations ranging from 0.1 to 30 μM. This range includes not only the *in vivo* concentrations of Redβ determined here (7–27 μM), but also previous measurements indicating a much lower value of 0.15 μM (25). The deconvolved spectra are shown in Supplementary Figure S13, and summarized in Figure 5 as a heat map that plots the relative intensities of each oligomer species present. As seen in Figure 5, for Redβ alone we observed a shift from predominantly monomer at 0.1 μM to a range of oligomers centered on 12 copies of Redβ at concentrations of 1 μM or greater. These measurements were repeated several times and while there was some variability in the range and relative intensities of oligomers observed at different concentrations, the most consistent trend was a shift from monomer to 12-mer as the Redβ concentration was increased.

**Figure 6.**
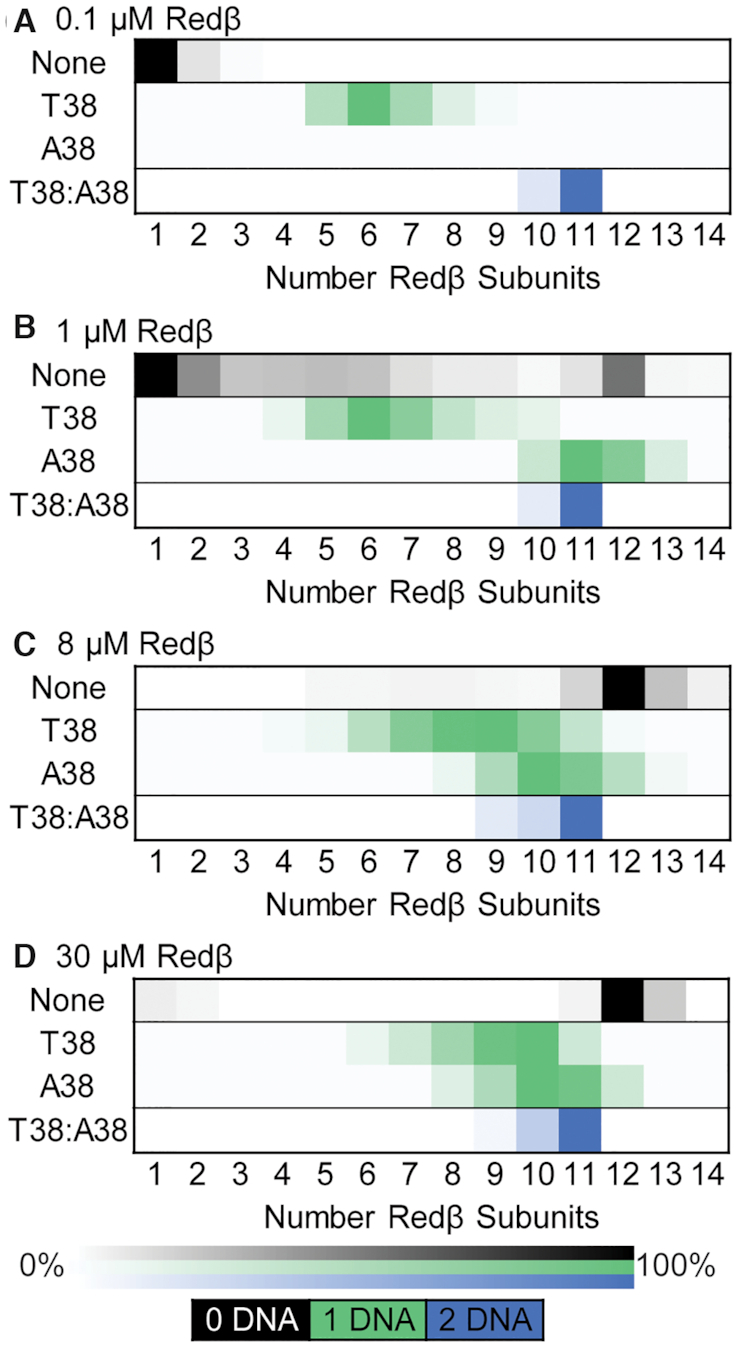
Native MS heat maps showing the distribution of Redβ oligomers formed in the absence and presence of 38-mer DNA. The heat maps indicate the relative intensities of the oligomer species present in the spectra of Supplementary Figure S14. The first row of each chart (labeled ‘None’) shows the Redβ oligomers formed at each concentration with no DNA (from Figure 5). The second (T38) and third (A38) rows show the DNA-containing oligomers formed by mixing either dT38 or dA38 ssDNA with Redβ. The fourth row (T38:A38) shows the DNA-containing oligomers formed by mixing dT38 and dA38 sequentially with Redβ. The coloring indicates the number of DNAs present in each complex: black (0 DNA), green (1 DNA) or blue (2 DNA).

Next, experiments were performed to measure the binding of Redβ to dT38 and dA38 as ssDNA, and as the annealed duplex formed when they were added to Redβ sequentially. These experiments were performed at a wide range of Redβ concentrations including 0.1, 1.0, 8.0 and 30 μM. For all complexes unless otherwise noted, each oligonucleotide was mixed with Redβ at a ratio of 4 nt/monomer, which was presumed to be stoichiometric. For example, 1 μM Redβ was mixed with 4 μM nucleotides of dT38. The resulting deconvolved mass spectra are presented in Supplementary Figure S14, and summarized as heat maps in Figure 6. In the case of dT38, the predominant species contained 5–8 subunits of Redβ at the lowest concentration (0.1 μM), but gradually shifted up to 6–11 subunits as the concentration was increased. In the case of dA38, no binding of Redβ was detected at the lowest concentration (0.1 μM), but the distribution quickly shifted up to 8–13 subunits at 1 μM and higher. The lack of binding to dA38 at 0.1 μM is likely due to the well-known self-folding of dA38, which may compete with nucleation of Redβ at low concentrations. This would not be the case for dT38, which is known for its absence of secondary structure. On the other hand, Redβ may have higher intrinsic affinity for dA38 than dT38, which would explain its earlier transition to larger complexes. In any case, these data clearly show that rather than forming a distinct complex containing a specific number of subunits bound to each 38-mer, such as the 11-mer ring seen for Rad52 (48), the Redβ-ssDNA complexes exist as a much more heterogeneous range of species, even at the highest concentration tested (30 μM).

**Figure 7.**
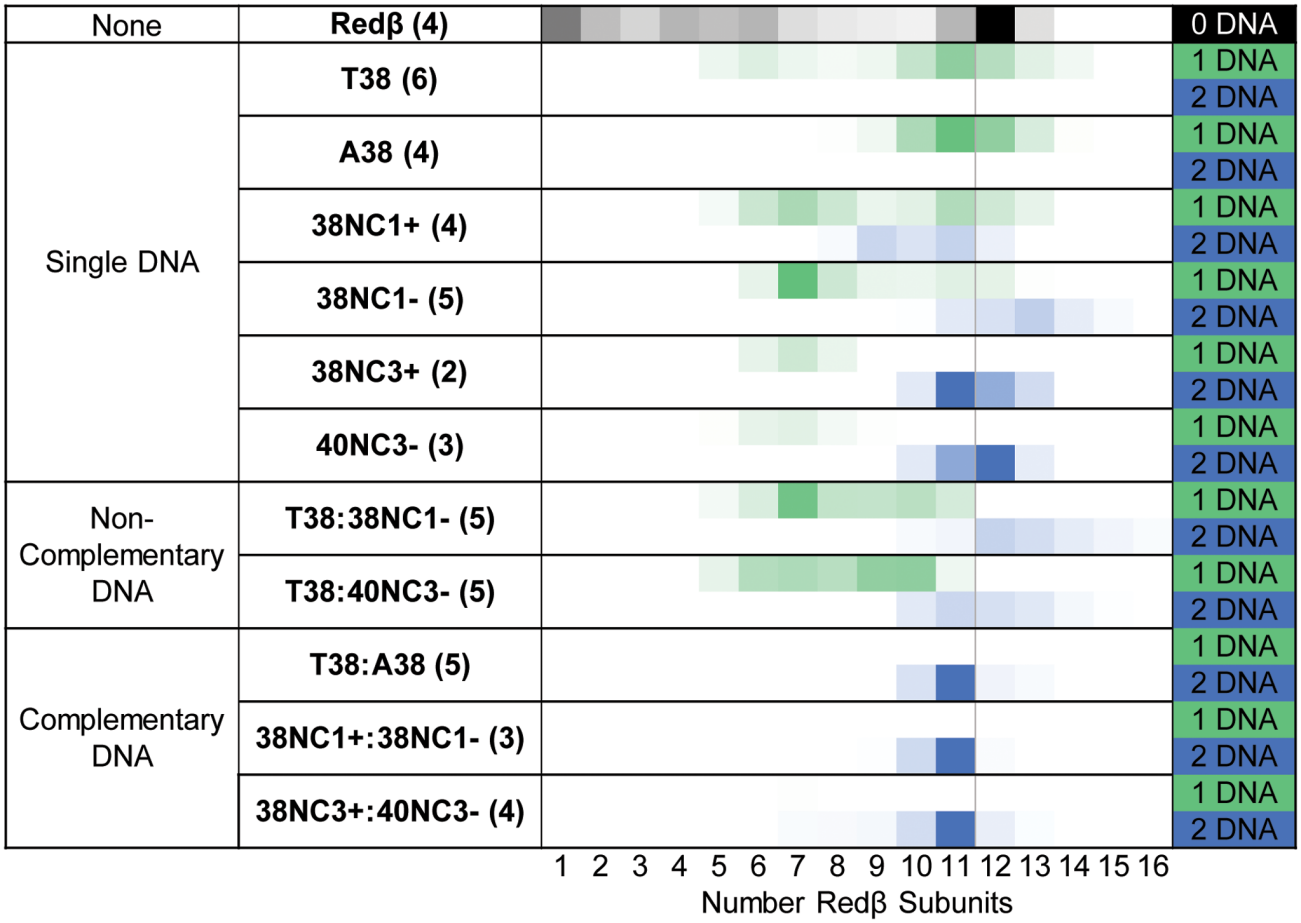
Native MS heat maps showing the distribution of Redβ subunits at 1μM in the absence and presence of 38-mer DNA. The heat maps show the relative intensities of each oligomer species, averaged over the number of spectra shown in parentheses. Representative spectra are shown in Supplementary Figures S14 and S15, and the bar charts of Supplementary Figure S16 show the averaging. The first row (labeled ‘None’) shows the Redβ oligomers formed at 1 μM with no DNA. The second set of rows (‘Single DNA’) shows the distribution of DNA-containing oligomers formed by mixing a single DNA with Redβ. The coloring indicates the oligomer observed with 1 (green) or 2 (blue) copies of DNA. The third set of rows (‘noncomplementary DNA’) shows the distribution of DNA-containing oligomers formed by mixing two noncomplementary DNAs sequentially with Redβ. The fourth set of rows (‘Complementary DNA’) shows the DNA-containing oligomers formed by mixing Redβ with two complementary DNAs (added sequentially). The order of ssDNA addition is indicated by the order in the labeling. The difference in mass between T38 and 40NC3- could be confidently distinguished but that between T38 and 38NC1- or 38NC3- could not, although the heat maps do not reflect this.

In stark contrast to the broad range of species observed with each 38-mer ssDNA, when dT38 and dA38 were added to Redβ sequentially, a dominant species containing 11 Redβ subunits and 2 DNA strands was observed at all four concentrations tested (Figure 6, blue squares). Complexes with 9 and 10 subunits of Redβ were also observed, but only as minor components. The resolution of the measurement was sufficient to clearly define this complex as having one copy of each strand. To further test if the stable complex was dependent on the two sequentially added strands being complementary to one another, four additional oligonucleotides were designed: 38NC1+ and its complement (38NC1-), and 38NC3+ and its complement (40NC3-), which contain essentially random sequences to better mimic naturally occurring DNA (Supplementary Table S4). The two extra nucleotides of 40NC3- were added to create a larger mass difference to ensure that complexes with two strands could be accurately assigned. Experiments with these oligonucleotides were performed at a concentration of 1 μM Redβ only. Due to a significant degree of variability, these experiments were performed multiple times, and presented as representative spectra in Supplementary Figure S15, as bar charts showing the relative amount of each species averaged from multiple spectra in Supplementary Figure S16, and summarized as heat maps in Figure 7.

As seen in the heat maps (Figure 7), when each of these four new DNAs was added to Redβ as ssDNA, a broad range of species containing 5–15 Redβ subunits was observed, similar to the complexes seen with dT38 and dA38. However, and unexpectedly, for all four of these random sequence DNAs, some of the species contained two copies of each ssDNA. The precise nature of these complexes is unclear, but they may contain two copies of the ssDNA paired with one another through microhomologies (Supplementary Figure S22). Similar results were obtained when two different but non-complementary pairs of oligonucleotides (dT38 and 38NC1-, or dT38 and 40NC3-) were added to Redβ sequentially. Both of these attempts at annealing two non-complementary DNAs gave complex mixtures of multiple species containing one or two ssDNAs (Figure 7, middle panel), similar to when each of the oligonucleotides were added to Redβ as single DNAs. By contrast, when two complementary oligonucleotides were added to Redβ sequentially, as dT38:dA38, 38NC1+:38NC1-, or 38NC3+:40NC3-, a dominant and distinct complex with 11 subunits of Redβ and one copy of each strand was observed in all three cases (Figure 7, bottom panel). These results clearly confirm that formation of the distinct and stable complex of Redβ with 11 subunits and two DNAs is only formed when the two sequentially added DNAs are complementary to one another.

**Figure 8.**
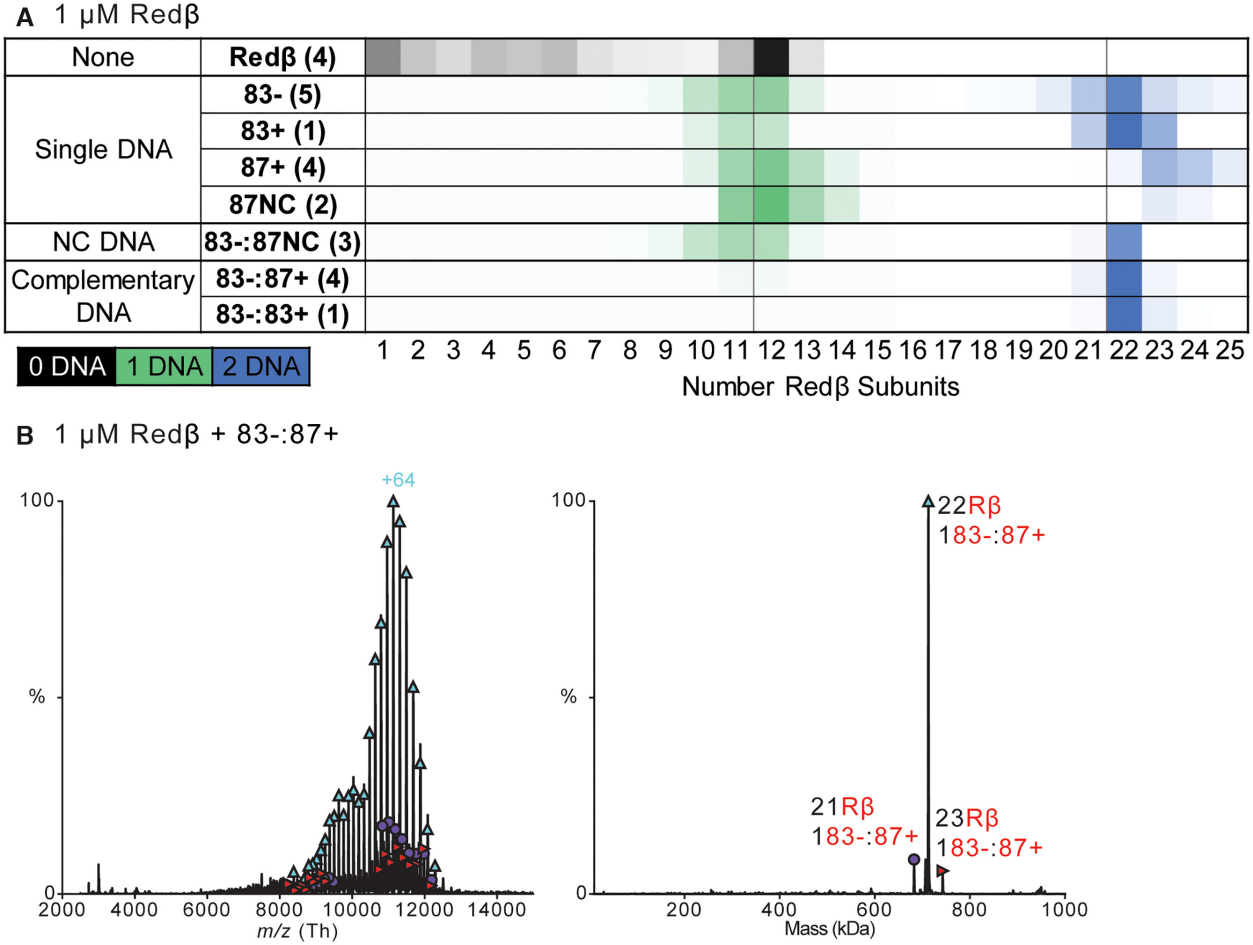
Oligomeric species observed from mixing 83-mer and 87-mer DNA with 1 μM Redβ. (**A**) Native MS heat maps showing the relative intensity of each species averaged over the number of spectra indicated in parentheses. Representative spectra are shown in Supplementary Figures S17 and S18, and the bar charts of Supplementary Figure S19 show the averaging. The first row (labeled ‘None’) shows the oligomers present at 1 μM with no DNA. The second set of rows (‘Single DNA’) shows the DNA-containing oligomers formed by mixing a single DNA with Redβ. The coloring indicates oligomers observed with 1 (green) or 2 (blue) copies of DNA. The third set of rows (‘NC DNA’) shows the distribution of DNA-containing oligomers formed by mixing two noncomplementary DNAs sequentially with Redβ. The fourth set of rows (‘Complementary DNA’) shows the DNA-containing oligomers formed by mixing two complementary DNAs sequentially with Redβ. The mass difference of oligomers containing 83- and 87NC or 87+ can be distinguished but is not reflected in the heat maps. (**B**) Mass spectrum (left) and zero-charge mass spectrum (right) of the complex containing predominantly 22 Redβ and 1 83-:87+ annealed duplex.

Next we tested the effect of DNA length with the naturally occurring 83-mer DNAs that were used for SEC-MALS (83- and 83+). These experiments were performed multiple times at 1 μM Redβ, and again shown as representative spectra in Supplementary Figure S17, as averaged bar charts in Supplementary Figure S19, and as heat maps in Figure 8. Again, when Redβ was combined with a single 83-mer ssDNA, two different types of complexes were observed. The first contained from 9 to 14 copies of Redβ bound to a single DNA, and the second, which was again unexpected, contained from 20 to 25 copies of Redβ bound to two strands of the same ssDNA. Note that in this case the species with two copies of each DNA contained significantly more subunits (20–25) than the species with one DNA (10–14). This contrasts with what was observed with the 38-mers, where the species with one and two copies of DNA contain roughly the same numbers of subunits (10–14).

Strikingly, when the two complementary 83-mer strands were added to Redβ sequentially to form the complex with annealed duplex, a predominant species containing 22 Redβ subunits and two DNA strands was observed. This complex is very similar to that seen with two complementary 38-mers, but exactly twice as large, in accordance with the roughly 2-fold increase in DNA length. However, in the case of the 83-mers, the assumption that the two strands were the complementary strands could not be validated, as the difference in mass between 83- and 83+ is only 288 Da, which results in an *m/z* difference of only 5 Th when 64 charges are present. Therefore, we added 4 nucleotides (TGAC) to the 3′ end of 83+ to make an 87+ oligonucleotide that is 1524 Da >83-, to give an *m/z* difference of 24 Th when 64 charges are present. The complex was then formed by the sequential addition of 83- and 87+ to 1 μM Redβ and subsequently analyzed by nMS. The 87+ was mixed at slightly <4 nt/monomer to match the molar concentration of 83-. Again, the dominant species containing 22 Redβ subunits and 2 DNA strands was observed, but this time the difference in mass allowed us to validate that the two strands are exclusively the complementary strands (Figure 8B).

As a negative control for the 83-mers, we designed an 87-mer of random sequence (87NC) that is noncomplementary to 83- and 83+, and added it to Redβ alone as ssDNA, or to Redβ that had been pre-incubated with 83- (deconvolved spectra shown in Supplementary Figure S18). When added to 1 μM Redβ as a single DNA, 87NC behaved similarly to 83-, 83+, and 87+, giving a broad range of species containing either one or two copies of the ssDNA (Figure 8). When 87NC was added to Redβ sequentially with 83-, the results were very similar: the species present resembled a combination of those found in the individual single DNA mixtures (Figure 8A). The deconvolution was able to pick up some species that contained both DNA strands and 22 Redβ subunits, but the more intense species with 22 Redβ subunits always contained two copies of the same DNA, predominantly 83- which was the first DNA added (Supplementary Figure S18B). These results confirm that formation of the distinct and stable complex with 22 copies of Redβ is dependent on the two strands of DNA being complementary to one another.

Finally, we tested binding of Redβ to pre-formed dsDNA. Based on prior studies with other methods (12,27), no binding was expected. The dT38:dA38 and 83-:87+ complementary pairs of ssDNA were combined in equimolar amounts, heated to 90°C for 5 min, and slowly cooled to anneal. The mass of the annealed DNA was measured to confirm stoichiometric formation of only dsDNA (Supplementary Figure S20A). Redβ was then separately added to pre-formed dT38:dA38 or 83-:87+ dsDNA to a final concentration of 1 μM and allowed to incubate at RT for 30 min prior to nMS analysis. Redβ plus pre-formed dT38:dA38 dsDNA resulted in predominantly free Redβ oligomers and unbound dsDNA. However, there was a very small amount of complex containing 11 Redβ and 1 dT38:dA38 (Supplementary Figure S20B). For Redβ plus pre-formed 83-:87+ dsDNA there was only free Redβ oligomers and unbound dsDNA, and no observable amount of a complex (Supplementary Figure S20C). These data support the conclusion that Redβ does not bind appreciably to pre-formed dsDNA.

A mass list including the determined masses, the theoretical masses based on their most likely assignments, and the corresponding difference in mass for all data are presented in a supplementary text file. Mass spectra with comparisons of collisional activation used for de-adducting and improved transmission of ions at higher *m/z* are presented in Supplementary Figure S21.

In summary, for the complexes with 38-mer and 83-mer annealed duplex, we observed dominant species with 11 and 22 Redβ subunits, respectively. This was in stark contrast to the broad range of species observed when Redβ was mixed with each ssDNA individually. In addition, the unexpected observation that two copies of the same ssDNA can form complexes with Redβ that are similar in size to complexes with two complementary ssDNA suggests that Redβ could be attempting to anneal the ssDNA to another copy of itself, at sites of partial self-complementarity. These data warrant further studies to investigate the ability of Redβ to anneal ssDNAs with varying degrees of partial complementarity.

## DISCUSSION

Crystal structures of the 11-mer ring formed by the Rad52 DNA-binding domain, both with and without bound ssDNA (19–20,48), give the strong impression of a distinct and stable oligomer of the protein that performs the annealing reaction. This impression is strengthened by EM images of several related SSA proteins such as Mgm101 (24), SAK (23) and Erf (21), which also revealed 11-mer rings as the single dominant species. EM images of Redβ revealed a similar oligomeric ring structure, but with a variable number of subunits that ranged from 11 to 12 in the absence of DNA to 15 to 18 with ssDNA (13). Moreover, when two complementary ssDNAs were added to the protein as long heat-denatured dsDNA, a dramatically different oligomeric complex was observed, a left-handed helical filament that presumably contained the protein bound along the duplex product (or intermediate) of annealing (13). AFM images also observed the helical filament structure for the annealed duplex state, but differed with EM for the other two states (16). For the protein alone, AFM revealed predominantly a split lock washer (also called a gapped ellipse) with 11–12 subunits instead of the closed ring that was apparent by EM (although some closed rings were also seen by AFM). For the complex with a 140-nt ssDNA, AFM revealed disperse monomers of Redβ bound along the ssDNA, instead of the oligomeric rings of 15–18 subunits seen by EM. This latter result suggested a different mechanism in which annealing is initiated by monomers of Redβ bound more weakly along the ssDNA, and then pushed to completion by stably clamped dimers of the protein bound to the annealed duplex intermediate (16,25). Collectively, these studies appear to suggest a dynamic oligomerization process involving multiple oligomeric states, but leave questions as to the precise nature of the oligomeric structures that exist in solution, and what their roles are during the reaction.

Here, as summarized in Table 3, we have used three complementary biophysical methods to probe the oligomerization of Redβ in its different DNA-bound states in solution. Overall, our data support the notion of a weak and dynamic oligomerization process, particularly for the ssDNA-bound state, and suggest that the annealing reaction is mediated not by a distinct structure of the protein such as a closed oligomeric ring, but rather by a range of different species with more varying oligomeric assemblies. In this discussion, we will review the data for each of the different DNA-bound states, and synthesize the relevant implications for the mechanism.

**Table 3.** Summary of oligomer data

	SEC-MALS			AUC (SV)			nMS		
Sample	*C* (μM)	(*n*)	nt or bp/mon	*C* (μM)	(*n*)	nt or bp/mon	*C* (μM)	(*n*)	bp/mon
Redβ alone	6.0	7.7	—	8.3	9.2	—	8.0	12	—
Redβ + dT38	10.5	14.0	2.7	8.3	12.5	3.0	1.0	9	—
Redβ + 83-	10.5	21.1	3.9	8.3			1.0	11	—
Redβ + dT38:dA38	5.1	11.3	3.4	8.3	11.5	3.3	1.0	11	3.5
Redβ + 83-:83+	3.2	18.2	4.6	8.3			1.0	22	3.8

The column labeled ‘*C*’ gives the concentration of Redβ in micromolar of monomer measured by A280 at elution (for SEC-MALS) or loaded in the experiment (AUC and nMS). The column labeled ‘(*n*)’ gives the number of subunits of Redβ in each complex based on the observed mass and assuming one copy of the ssDNA or annealed duplex. The column labeled ‘nt or bp/mon’ gives the stoichiometry of each complex in nucleotides (or base pairs) per monomer of Redβ. For AUC, the data for only the lowest concentration are presented, but the values at higher concentrations of Redβ are similar (Table 2). For nMS, only the bp/mon is given (for the complexes with annealed duplex), as the species with ssDNA were so diverse.

### Without DNA

The three methods give similar results for the size of the oligomer in the absence of DNA: 8–9 subunits are seen by SEC-MALS, 9–13 subunits by SV, and a distribution with a dominant species of 12 subunits by nMS. Compared to nMS and SV, SEC-MALS appears to underestimate the size by 3–4 subunits. This is likely due to the rapid and transient dissociation of oligomer into monomers that was previously detected by DLS (26), which would manifest as a lower weight-average measurement by SEC-MALS (47). The dominant 12-mer observed by nMS at >1 μM matches the size of the oligomer detected by DLS (26) and the number of subunits seen in the rings (or split lock washers) by EM (13) and AFM (16). Although our data do not report on shape, the fact that the nMS data at higher concentrations (8–30 μM) converge on the 12-mer, as opposed to giving increasingly larger species, is consistent with an oligomeric structure that closes up on itself to reach a maximum number of subunits. The nMS data, which were collected over a wide range of protein concentrations (0.1–30 μM), indicate that the midpoint of the transition from monomer to 12-mer occurs at approximately 1 μM. This is in close agreement with previous measurements by fluorescence correlation spectroscopy (25). However, even at the second highest concentration examined (16 μM), species ranging from 1 to 14 subunits were detected, indicating a relatively weak and dynamic oligomerization process for free Redβ protein.

### With ssDNA

Considering first the complexes with 38-mer ssDNA, the three methods give similar results: a complex with 14 subunits is apparent by SEC-MALS (for dT38), 12–16 subunits by SV (for dT38), and 5–14 subunits by nMS (on dT38, dA38, and four random sequence 38 mers). The nMS data are particularly illuminating in that they reveal a much broader range of species than expected, including complexes with one DNA that are only partially occupied, as well as complexes with two copies of the same DNA. If these species are present during the SEC-MALS and SV measurements they are either not resolved by these lower-resolution methods, or exist too transiently to be resolved. Along these lines, it is likely that nMS, being a gas phase measurement that can kinetically trap species released from solution (49–52) is better able to capture transient subunit dissociation, whereas for SEC-MALS and SV, both dissociation and re-association can occur during the measurement.

Regarding the partially occupied complexes detected by nMS, we envision that they could be formed in two fundamentally different ways. First, they could have Redβ subunits bound along the ssDNA discontinuously in a noncooperative manner, leaving un-occupied gaps. Such a binding mode has in fact been observed by AFM on a 140-mer ssDNA (16). On the other hand, Redβ has been shown to bind preferentially to DNA ends (12), specifically to the 3′-end, and fluorescence polarization titrations indicate that Redβ binds to ssDNA cooperatively (27). Thus, it is conceivable that Redβ could nucleate at the 3′-end to form an oligomeric complex that could transiently dissociate from the 5′-end. The nMS data cannot distinguish between these two possibilities. Nonetheless, the data clearly show that Redβ-ssDNA complexes do not exist as a distinct and uniform oligomer like the 11-mer ring seen for Rad52, but rather as a much more broad and heterogeneous range of species.

nMS also detected complexes that contain two copies of the same ssDNA. These were only observed for the random-sequence 38-mers and not for dT38 and dA38, suggesting that they likely arise from partial self-complementarity of the ssDNA. These complexes contain roughly the same numbers of subunits (eight to fourteen) as the complexes with one copy of ssDNA (five to thirteen), and thus do not appear to be formed by dimerization of two ‘unit’ Redβ-ssDNA oligomers. Conceivably, these complexes could be formed by binding of a free ssDNA to an existing Redβ-ssDNA complex through regions of partial complementarity. If so, these complexes could provide a window into the transient intermediates that exist during the homology search step of the annealing process. Along these lines, it is conceivable that these complexes could help to explain the weaker ∼12-pN interactions that were observed in the single-molecule unzipping experiments reported by Ander *et al.* (25). Although the authors interpreted these events as arising from a Redβ monomer interacting with two segments of ssDNA during unzipping, their data did not establish the number of subunits directly.

For the complexes on 83-mer ssDNA, SEC-MALS gave a single peak corresponding to a complex with 21 subunits of Redβ, while nMS again indicated a much broader range of species, including complexes with 9–14 subunits of Redβ bound to a single copy of the ssDNA, and with 20–25 subunits bound to two copies of the ssDNA. Importantly, there was a key difference between 38-mer and 83-mer ssDNA. On the 83-mers the complexes with two copies of ssDNA had roughly twice as many subunits (20–25) as the complexes with one copy (9–14). This was not the case for the 38-mers, where the complexes with one and two copies of ssDNA had roughly the same number of subunits (9–14). It is informative that for the 83-mers, the complexes with one copy of ssDNA do not get larger than a 14-mer, while the complexes with two copies of ssDNA do not get much smaller than a 20-mer. In this sense, it is possible that the complexes with two copies of DNA could be a dimer of a ‘unit’ Redβ-ssDNA oligomer, such as two ring-ssDNA complexes interacting through the ssDNA. Such a complex has in fact been observed for Rad52 (48). However, it is also conceivable that the two types of complexes are fundamentally different from one another. For example, the complexes with 10 to 14 subunits on one ssDNA could be a ring form of the protein bound one end of the 83-mer ssDNA, while the complexes with 20–25 subunits and two copies of ssDNA could be a filament form of the protein bound along two copies of ssDNA in an attempt at annealing. Consistent with this latter possibility is the fact that the larger complexes contain roughly the same number of subunits, 20–25, as the stable complex that is formed when the two complementary 83-mers are added to Redβ sequentially (22 subunits).

### With two complementary ssDNAs

For the complex with two complementary 38-mer ssDNAs added sequentially (dT38 and dA38), the three methods are consistent: an oligomer with 11 subunits is seen by SEC-MALS, 12–15 subunits by SV, and 11 subunits by nMS. The nMS data are particularly striking for this complex in that the distribution narrows to a distinct species with 11 subunits; species with 9 and 10 subunits are also seen, but only as minor components. This is in stark contrast to the nMS data for the ssDNA complexes, which gave much broader distributions with no single species being prevalent. In this sense, the nMS data aptly highlight the dramatically increased stability and specificity of the complex with annealed duplex that has been seen by gel shift (12,25–28), and also observed as the remarkably stable >60 pN complex by single-molecule force measurements (25).

The data for the complex with two complementary 83-mers is similar: 18 subunits is seen by SEC-MALS, and 22 subunits by nMS. Again, the nMS data are striking in that the distribution narrows to a distinct species of 22 subunits, with 21- and 23-subunit complexes present only as minor components. Interestingly, from the nMS data, the complex with two complimentary 83-mers is exactly twice as large as the complex with two 38-mers (22 versus 11 subunits), consistent with the continuous oligomerization process that would be expected for a helical filament. The same trend, a roughly linear relation between the length of the DNA and the contour length of the filament was seen by EM (13) and AFM (16).

In summary, the nMS data reveal a striking difference between the complexes formed when one ssDNA was mixed with Redβ, and those formed when two complementary ssDNAs were added to Redβ sequentially. With one ssDNA, a remarkably broad range of species was observed, including partially dissociated complexes and complexes with two copies of the ssDNA. By contrast, when two complementary oligonucleotides were added to Redβ sequentially, a much more distinct and apparently stable complex was formed.

### With two noncomplementary ssDNAs

Mixtures of Redβ with two noncomplementary oligonucleotides added sequentially were examined as negative controls, to test if the formation of the remarkably distinct and stable complexes described above was dependent on homology. In the gel-based annealing assay with 50-mer oligonucleotides, complexes with both strands were only observed if the two 50-mers were complementary to one another. If the second strand added was noncomplementary to the first, Redβ remained bound to the first strand, and no interaction with the second strand was detected.

In the nMS experiments with 38- or 83-mers, the complexes formed with two noncomplementary oligonucleotides essentially matched what was observed when only one ssDNA was added: a broad range of oligomers with no distinct species being prevalent. There were small amounts of complexes containing one copy of each strand, but these were not as prevalent as when the two sequentially added ssDNAs were complementary to one another. All together, the negative control experiments clearly show that formation of the distinct and stable complex is dependent on the two strands being complementary to one another.

### With pre-formed dsDNA

One of the most intriguing properties of Redβ is that it binds most stably to a duplex intermediate of annealing formed when two complementary strands of ssDNA are added to the protein sequentially, yet shows no interaction when the same oligonucleotides are pre-annealed to form dsDNA. This lack of binding to pre-formed dsDNA was previously shown definitively by gel-shift (12) and by fluorescence polarization (27). Binding of Redβ to long dsDNA was observed by TEM, but only when the ends of the DNA were resected by nucleases (13). Here, binding to pre-formed dsDNA was tested by nMS. Although a very slight amount of binding to pre-formed 38-mer duplex was detected, no binding was observed for the 83-mer duplex. The nMS data thus reinforce the lack of binding to dsDNA that has been seen by the other methods.

### Stoichiometry of the protein relative to the DNA

Our data provide new insights into the stoichiometry of the Redβ complexes with ssDNA substrate and annealed duplex intermediate. For the ssDNA complex, we expected a stoichiometry of 4 nt/monomer, based on the homology with Rad52, for which the stoichiometry has been clearly established by crystal structures and DMS footprinting (20,48). The data from SEC-MALS and SV, which gave stoichiometries ranging from 2.7 to 3.9 nt/monomer, are generally consistent with this assumption. Although a wider range of species was observed by nMS, the most fully occupied complex of Redβ on ssDNA (13 Redβ on dA38) has a stoichiometry of 2.9 nt/monomer.

The stoichiometry of the Redβ complex with the annealed duplex intermediate, for which there is no known Rad52 complex for comparison, has been less clear from prior studies. Analysis of the contour lengths observed in AFM images with three different lengths of complementary oligonucleotides led to an estimate of 11 bp/monomer (16). Similar analysis of TEM images led to a value of 5.8 bp/monomer (13). Both of these methods relied on estimates for the volume and shape of the Redβ monomer based on its mass of 29.7 kDa. The data from the three methods reported here indicate stoichiometries ranging from 3.1 to 4.6 bp/monomer for this complex. The distinct complexes seen in the nMS data are particularly informative in this regard, giving stoichiometries of 3.5 and 3.8 bp/monomer for the 38-mers and the 83-mers, respectively. These data suggest that the stoichiometries of the complexes on ssDNA substrate and annealed duplex product are not dramatically different, as might have been expected from previous studies (16).

Importantly, our value of ∼4 bp/monomer for the stoichiometry of the complex with annealed duplex has significant implications for Redβ’s mechanisms of annealing. First, the observation that the two complexes have similar stoichiometries suggests that the degree of reorganization (or reassembly) that takes place during the transition from ssDNA substrate to annealed duplex intermediate may not be so extensive after all. Second, previous studies have determined that formation of the stable complex with annealed duplex requires oligonucleotides of at least 20 bp in length (16,25), and our experiments in PBS give a very similar result (Supplementary Figure S6). Based on their estimate of ∼10 bp/monomer for the stoichiometry, the authors of the previous study concluded that the remarkably stable complex on 20 bp of annealed duplex contained only two monomers of Redβ, and thus concluded that a dimer of Redβ is the minimal unit needed to promote annealing. By contrast, our measurement of the stoichiometry of ∼4 bp/monomer for this complex indicates that it would contain at least five subunits of Redβ. Complexes formed on the 40- to 100-base DNA homologies typically used for *in vivo* recombineering could contain up to 10–25 subunits of Redβ.

### The concentration of Redβ expressed in cells active for recombination

We also measured the concentration of Redβ expressed *in vivo* using two common expression systems. Our measurement of 7 ± 2 μM for the arabinose-inducible pSC101 plasmid is 35-fold higher than a previous measurement of 0.2 μM that used the same (or very similar) plasmid and cell line (25). Our experimental design was also based on this earlier report, and our calculation used the same values for the volume of an *E. coli* cell and cell density per OD_600_. The two studies may have differed in the preparation of SDS-PAGE gel samples. In our study gel samples were prepared from the soluble fraction of centrifuged lysates produced by sonication. Thus, if anything, our measurement could have been an underestimate if some of the protein was in the pellet fraction. The two studies also used different culture conditions, different antibodies, and different versions of purified recombinant Redβ as protein standard. Our study used Redβ with an extra Gly-Ser-His at the N-terminus, whereas the previous study used Redβ with extra C-terminal residues from a strep-affinity tag. Which if any of these factors could account for the 35-fold difference in measured *in vivo* concentration is unclear. It is worth noting however that a third measurement of Redβ expression from pSC101 gave 2.3 ± 0.3 μM (53), considerably closer to our value.

For reference, endogenous expression levels of two other recombination proteins, RecA and SSB, have been reported to be 1–10 μM and 0.5–1 μM, respectively (54,55). Based on the more generalized functions of these proteins, one might think it surprising that the concentration of Redβ would be higher. However, given that in the pSC101 system Redβ is being over-expressed from a multi-copy plasmid with a strong inducible P_BAD_ promoter (56), in our mind it is to be fully expected that the expression level would be higher. This level of expression is still however far lower than what is typical from the T7-based pET vectors that are commonly used to express proteins for purification, as the band for Redβ expressed from pSC101 on an SDS-PAGE gel is still not prominent relative to host proteins (data not shown).

We also measured the concentration of Redβ expressed from the native lambda P_L_ operon under control of a temperature sensitive λ repressor, in this case from the pSIM5 plasmid (29). We find that the expression level from this system is 27 ± 12 μM, which is approximately 4-fold higher than for the arabinose-inducible system. This observation is consistent with prior conclusions that higher levels of recombination with the lambda P_L_ operon-based system are due to its higher levels of Redβ expression than from the arabinose-inducible P_BAD_ systems (29). The concentration of 27 μM observed for pSIM5 is also more likely to reflect the *in vivo* context for which red function has evolved, as this plasmid uses the natural lambda P_L_ promoter.

If we assume that the minimum concentration of Redβ required for recombination *in vivo* is 0.15 μM as reported previously (25), the nMS data indicate that Redβ would exist predominantly as monomers in the absence of ssDNA, as multiple monomers bound to short ssDNA, and as a continuous and stable complex on annealed duplex, with one monomer of Redβ for every 4 bp of duplex. If the cellular concentration of Redβ is considerably higher, such as at the 7–27 μM concentrations determined here, the nMS data indicate that the implications for the complexes with ssDNA and annealed duplex would be similar, but the 12-mer oligomers of the free protein would be more prevalent, and may have to dissociate before binding to ssDNA. This would apparently not hinder DNA binding significantly, however, as the SEC-MALS and prior DLS (26) data collectively indicate that monomers of Redβ can rapidly and transiently dissociate from the protein-only oligomers. Consistent with this conclusion, Redβ is active for *in vitro* annealing at a concentration of 10 μM (reference 26 and Supplementary Figure S5), suggesting that the window of Redβ concentration for annealing activity does not need to match the window of the monomer to oligomer transition, as was suggested by Ander *et al.* (25). Finally, at least two studies have demonstrated that higher levels of Redβ expression *in vivo* correlate with higher levels of recombination (29,30).

In conclusion, this study provides new insights into the range of oligomers formed by Redβ in the three different states that are relevant to the reaction. A relatively uniform 12-mer is seen in the absence of DNA, a broader range of partially occupied complexes and complexes with two copies of ssDNA is seen on ssDNA substrate, and a much more uniform and apparently stable complex is seen on annealed duplex intermediate, with a stoichiometry of 4 bp/monomer. Measurements of the concentration of Redβ expressed in cells active for recombineering range from 7 to 27 μM, within the range of our *in vitro* measurements. Importantly, the stoichiometry of 4 bp/monomer measured by nMS precisely matches the stoichiometry observed for the ssDNA complex of Rad52, suggesting the possibility of a common mechanism of annealing for this family of distantly related SSA proteins. However, the stable helical filaments observed for Redβ on annealed duplex have so far not been seen for Rad52, and whether or not the oligomeric Rings of Rad52 readily dissociate into monomers during annealing reactions remains to be determined. Our study also provides a comparison of three methods used for sizing analysis of protein–DNA complexes. nMS is found to be particularly powerful for dissecting out the precise protein-DNA species present in complex mixtures. The need to dialyze (or buffer exchange) samples into ammonium acetate did not appear to have a significant effect on the complexes observed, and, as is typical for nMS, the complexes remain associated (kinetically trapped) despite being transported to the gas phase. Future studies of nMS for this system will examine the size of complexes formed on additional lengths of ssDNA and with varying degrees of complementary to gain further insights.

## Supplementary Material

gkab125_Supplemental_Files

## Notes

Present address: Christopher E. Smith, Laboratory of Protein Dynamics and Signaling, Center for Cancer Research, National Cancer Institute, Frederick, MD, 21702-1201, USA.
