# Supplementary material for: Oligomeric complexes formed by Redβ single strand annealing protein in its different DNA bound states
Source: Nucleic Acids Res. 2021 Mar 8;49(6):3441–60. doi: 10.1093/nar/gkab125 (PMC8034648; doi:10.1093/nar/gkab125)
Supplement: gkab125_Supplemental_Files [file gkab125_supplemental_files.zip › Caldwell_etal_SI_rev2.pdf]

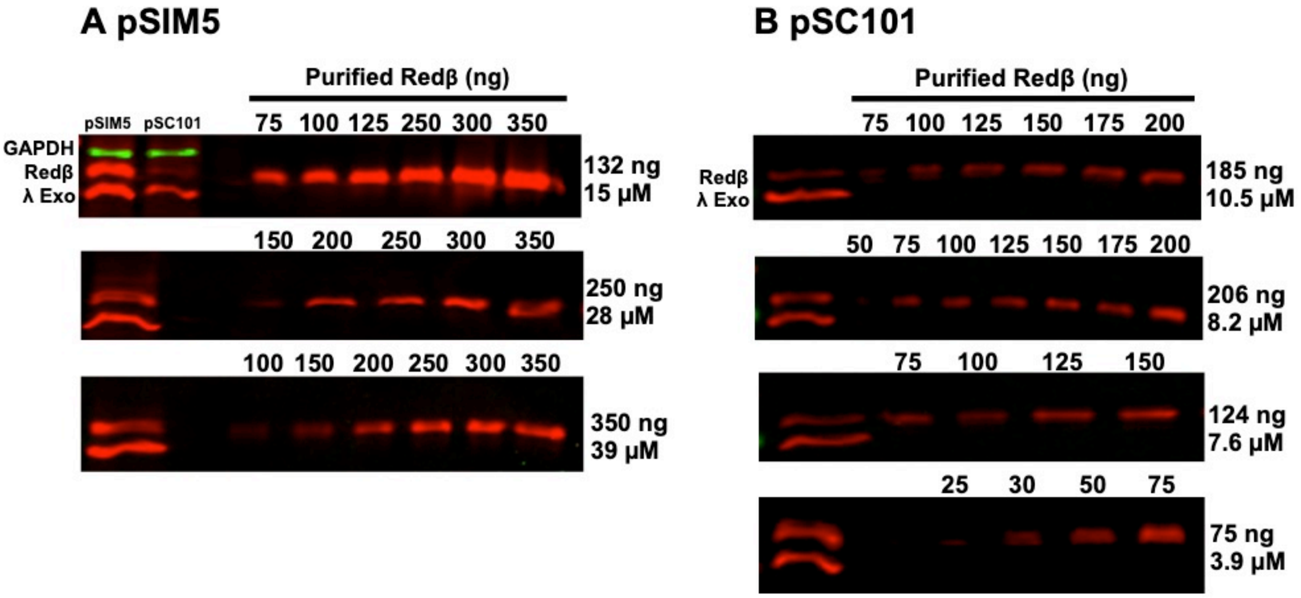

Figure S1. Western blots to determine the concentration of Redβ expressed from pSIM5 and pSC101 plasmids. From quantitative comparisons to standard curves generated from known amounts of purified Redβ, multiple experiments for **(A)** pSIM5 and **(B)** pSC101 result in average *in vivo* Redβ concentrations of 27 ± 12 and 7 ± 2 μM, respectively (0.81 and 0.20 mg/mL). The numbers to the right of each gel give the measured amount of Redβ loaded onto the gel from 10-16 μL of lysate, along with the resulting value calculated for the concentration of Redβ *in vivo*. Details of the calculations are provided below in Table S1.

Table S1. Full calculations for determining the *in vivo* concentration of Redβ. The final concentration (μM) of Redβ in the far right column was determined by dividing the value for μg/cell by the volume of an *E. coli* cell (3.8×10^{-9} μl) and by the MW of native Redβ (29,689 g/mol).

Sample	Amt. of protein on gel (ng)	Lysate loaded (μL)	Total Lysate (mL)	Total protein in lysate (μg)	OD_{600} of culture post-induction	Culture volume (mL)	Total no. of cells ($\times 10^{10}$)	μg/cell ($\times 10^{-9}$)	μM Redβ
pSIM5-1	132	10	2.5	33	0.71	35	1.99	1.7	15
pSIM5-2	250	16	2.5	39	0.44	35	1.23	3.2	28
pSIM5-3	350	16	2.0	44	0.35	35	0.98	4.5	40
pSC101-1	185	10	2.0	37	0.78	50	3.12	1.2	11
pSC101-3	206	15	2.0	28	0.74	50	2.96	0.95	8.4
pSC101-2	124	10	2.0	25	0.72	50	2.88	0.87	7.7
pSC101-4	75	15	2.2	11	0.63	50	2.52	0.44	3.9

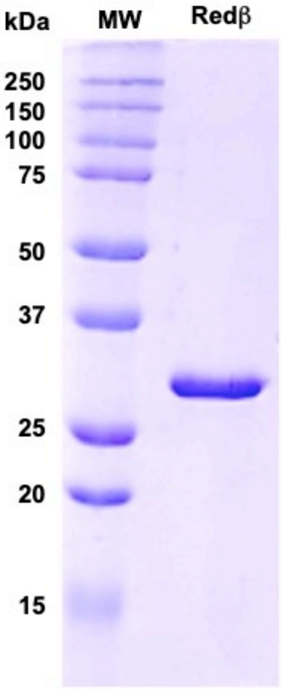

Figure S2. SDS-PAGE of purified Redβ protein used in this study. 1.5 μg of purified Redβ protein (diluted from 48 mg/ml stock) was loaded onto a 12.5% SDS-PAGE gel for analysis of purity and comparison to MW Standards. The calculated FW of Redβ with N-terminal Gly-Ser-His is 29.97 kDa.

	Oligonucleotides	Sequence
DNA Annealing Assay	Cy5 50mer	5′-CCATCCGCAAAAATCGAGCTATGCAGGGCGATTCTGCTCTAAGCCATCCG-3′
	Cy3 50mer	5′-GCGGATGGCTTAGAGCAGAATCGCCCTGCATAGCTCGATTTTTGCGGATG-3′
	Cy3 NC 50mer	5′-GAGTGATAGGCATATGTGCTACGAGTATCGAAGGAACGGTCACTACGCAA-3′
	Cy5 28mer	5′-AAAATCGAGCTATGCAGGGCGATTCTGC-3′
	Cy3 28mer	5′-GCAGAATCGCCCTGCATAGCTCGATTTT-3′
	Cy5 24mer	5′-AAAATCGAGCTATGCAGGGCGATT-3′
	Cy3 24mer	5′-AATCGCCCTGCATAGCTCGATTTT-3′
	Cy5 20mer	5′-AAAATCGAGCTATGCAGGGC-3′
	Cy3 20mer	5′-GCCCTGCATAGCTCGATTTT-3′
	Cy5 16mer	5′-AAAATCGAGCTATGCA-3′
	Cy3 16mer	5′-TGCATAGCTCGATTTT-3′
SEC-MALS, AUC, nMS	dT38	5′-TTTTTTTTTTTTTTTTTTTTTTTTTTTTTTTTTTTTTT-3′
	dA38	5′-AAAAAAAAAAAAAAAAAAAAAAAAAAAAAAAAAAAAAA-3′
SEC-MALS, nMS	83+	5′-TTGATAAGAGGTCATTTTTGCGGATGGCTTAGAGCTTAATTGCTGAATCTGGTGCTGTAGCT CAACATGTTTTAAATATGCAA-3′
	83-	5′-TTGCATATTTAAAACATGTTGAGCTACAGCACCAGATTCAGCAATTAAGCTCTAAGCCATCC GCAAAAATGACCTCTTATCAA-3′
nMS	87+	5′-TTGATAAGAGGTCATTTTTGCGGATGGCTTAGAGCTTAATTGCTGAATCTGGTGCTGTAGC TCAACATGTTTTAAATATGCAATGAC-3′
	87+ NC	5′-GGTTATGTTTCAAGCGCACCTAATGCTAGAGTTATAGGTGGCAACTGCGACTCACGCCGCT AATCAGGCCGCACTTCCATAGCTCGA-3′
	38NC1+	5′-AGACACAGTAAACGGACGCTAGATGCAACGAGATCCGA-3′
	38NC1-	5′-TCGGATCTCGTTGCATCTAGCGTCCGTTTACTGTGTCT-3′
	38NC3+	5′-GACGCACAATGACAGATAACACAATGGCGAGATAACGC-3′
	48NC3-	5′-GCGTTATCTCGCCATTGTGTTATCTGTCATTGTGCGTCGC-3′

Figure S3. Sequences of all oligonucleotides used in this study.

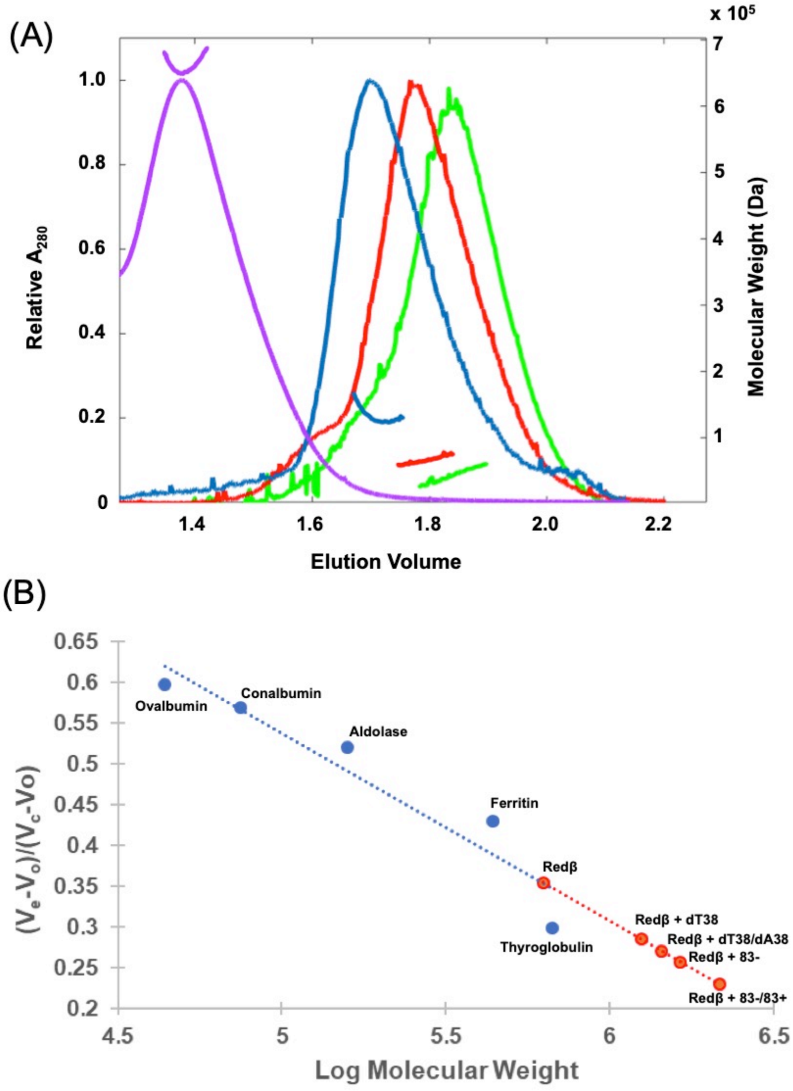

Figure S4. SEC-MALS analysis of protein standards. **(A)** MALS masses of ovalbumin (Green), conalbumin (Red), aldolase (Blue), and thyroglobulin (purple) were measured to be 41, 66, 132 and 662 kDa, respectively. The calculated masses are 44, 75, 158, and 669 kDa, respectively. The horizontal line under (or over) each peak gives the measured MALS mass, as indicated on the right-hand scale. **(B)** Standard curve used to determine SEC masses reported in Table 1. The total proportion of pores available to a molecule is plotted vs. log MW. Blue points correspond to the MW standards used to calibrate the column. Red points correspond to complexes of Redβ. The MALS masses of the Redβ complexes are smaller than thyroglobulin, yet elute later, presumably due to their non-spherical shapes.

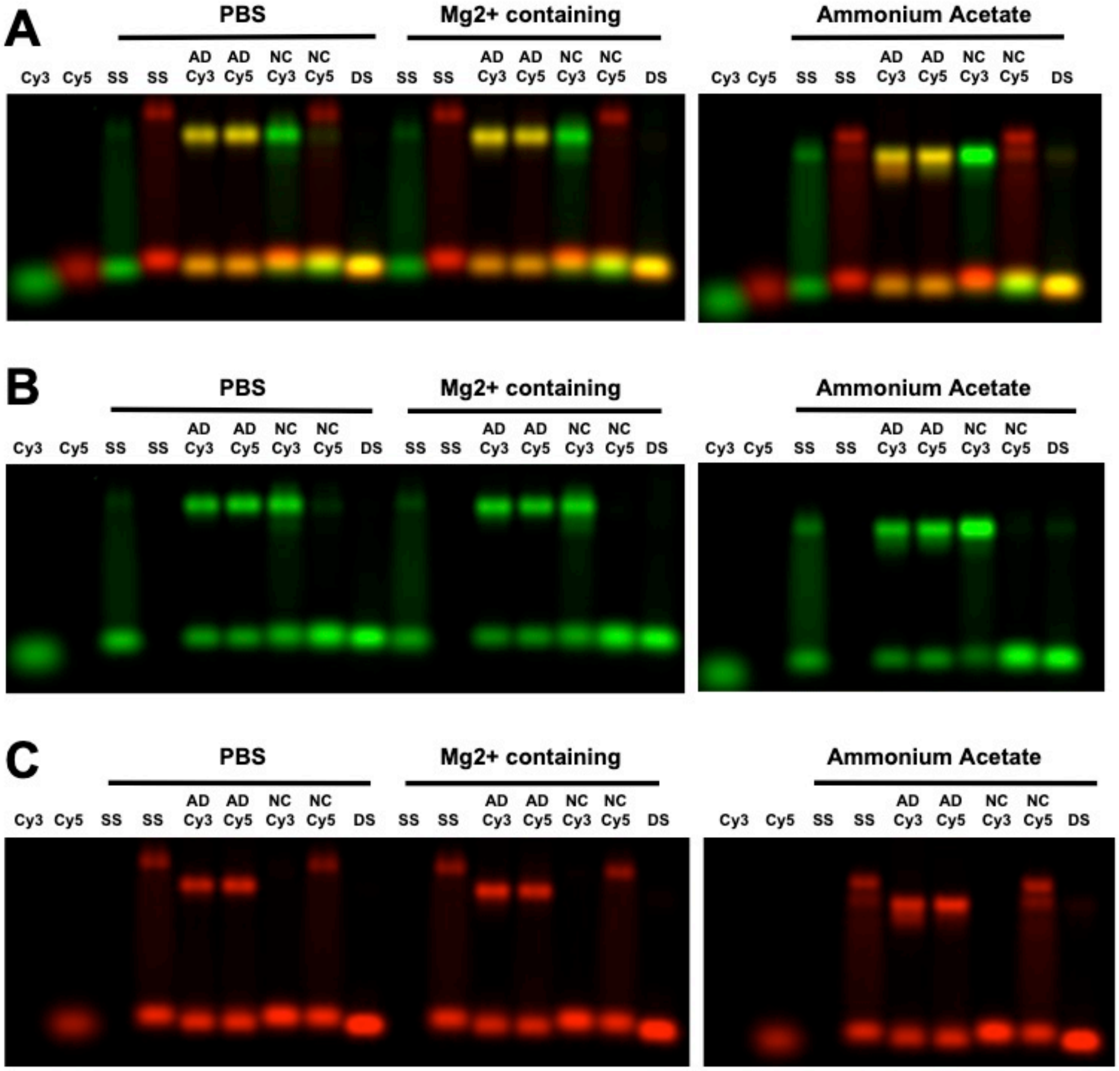

Figure S5. Gel-based DNA annealing assays in three different buffers: PBS, Mg^{2+}-containing, and 50 mM ammonium acetate. The 50-mer oligonucleotides were 5'-labeled with either Cy3 (green) or Cy5 (red). Full sequences are given in Figure S3. The first two lanes of each gel contain each oligonucleotide without protein added. The other lanes contain 10 μM Redβ incubated with 50 μM nucleotides of either one (lanes labeled "SS"), or two complementary 50-mer oligonucleotides (lanes labeled "AD" or "DS"). For the AD lanes, the two oligonucleotides were added to Redβ sequentially to form the complex with annealed duplex, with the indicated strand (Cy3 or Cy5) added first. For the DS lanes, the two oligonucleotides were annealed together prior to adding Redβ. Lanes labeled "NC" contain Redβ incubated sequentially with two non-complementary oligonucleotides (Cy5 50mer and Cy3 NC 50mer), with the indicated strand (Cy3 or Cy5) added first. Notice that Redβ interacts weakly with each individual 50-mer oligo as indicated by the faint streaking bands seen in the SS lanes. Redβ forms a much more stable complex when two complementary oligos are added sequentially ("AD"), as seen by the prominent band of yellow color, indicating that it contains both strands. (**A**) Dual channel exposure showing both Cy3 (green) and Cy5 (red) oligonucleotides. (**B,C**) Single channel exposures showing only Cy3 or Cy5, respectively.

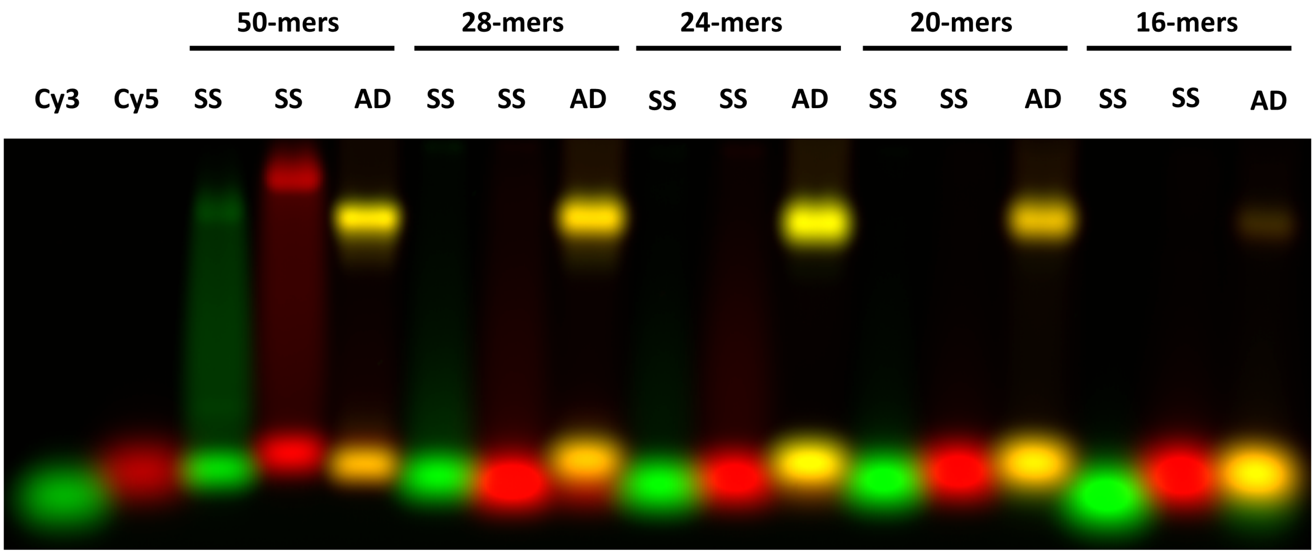

Figure S6. Gel-based DNA annealing assay for Redβ with different lengths of complementary oligonucleotides. The 50-mer oligonucleotides were 5'-labeled with either Cy3 (green) or Cy5 (red). Full sequences are given in Figure S3. The first two lanes contain each oligonucleotide without protein added. The other lanes contain 10 µM Redβ incubated with 50 µM nucleotides of either one (SS lanes) or two complementary 50-mer oligonucleotides added sequentially (AD lanes). Redβ interacts weakly with each individual oligonucleotide (SS lanes), as seen by the faint shifted bands with streaking for the 50-mers, and by the lack of shifted bands for the shorter oligos. Redβ forms a much more stable complex when the two complementary oligos are added sequentially (AD lanes), as seen by the prominent band of yellow color, indicating that it contains both strands. Notice that the AD complex is not formed prominently in the sample with 16-mer oligonucleotides, indicating that the minimum length (of those tested) for stable complex formation is 20.

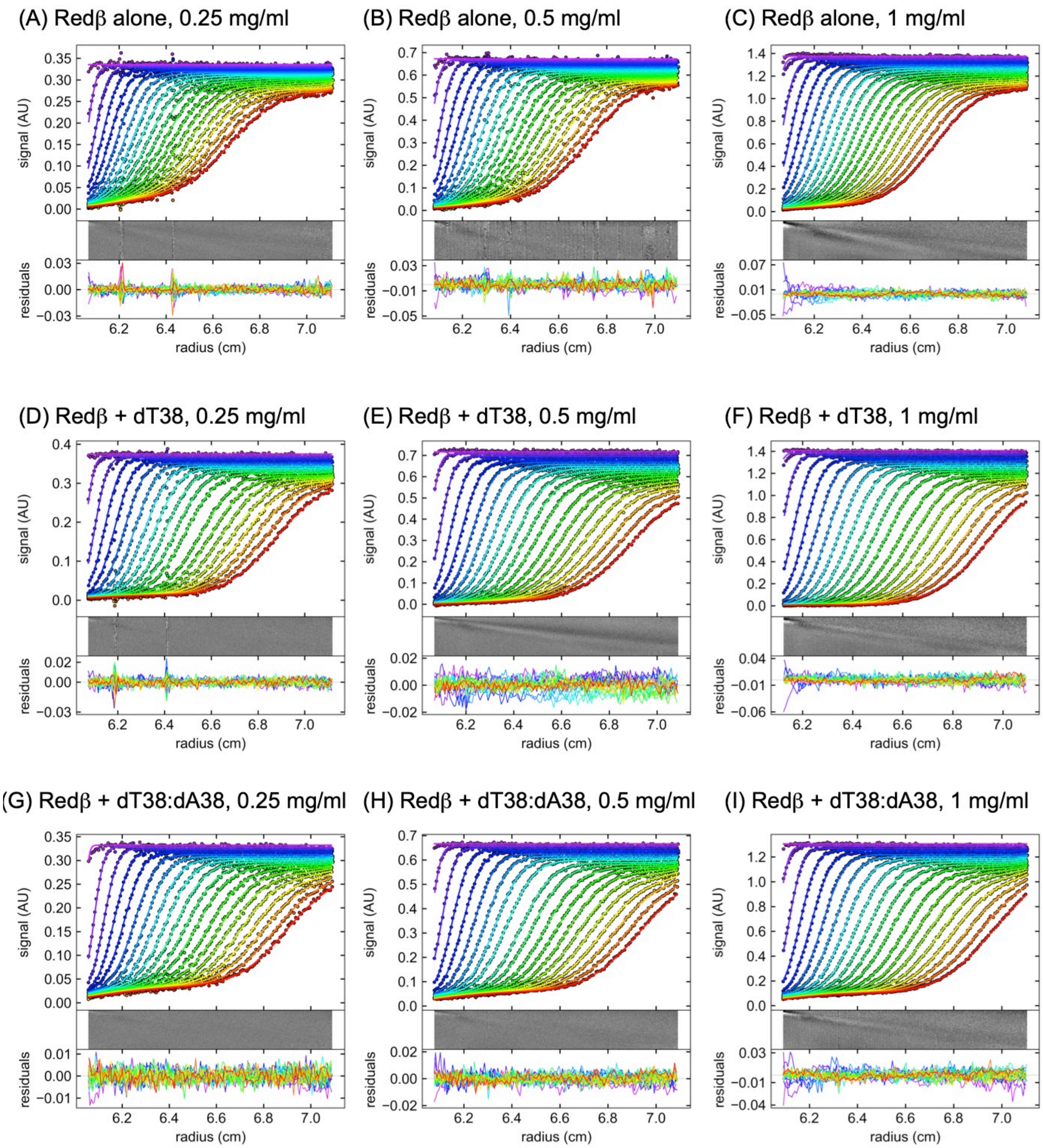

Figure S7. Quality of fits for SV A280 data. Fits of the SV A280 data for each sample were performed using the *c(s)* analysis in SEDFIT (36) and plotted with GUSSSI (37). Only every 3rd trace (out of 60 total) is shown, with raw data points as circles. The fit from SEDFIT is shown as the smooth line through each trace, with the 2D bitmap (in greyscale) and the residuals shown below each plot.

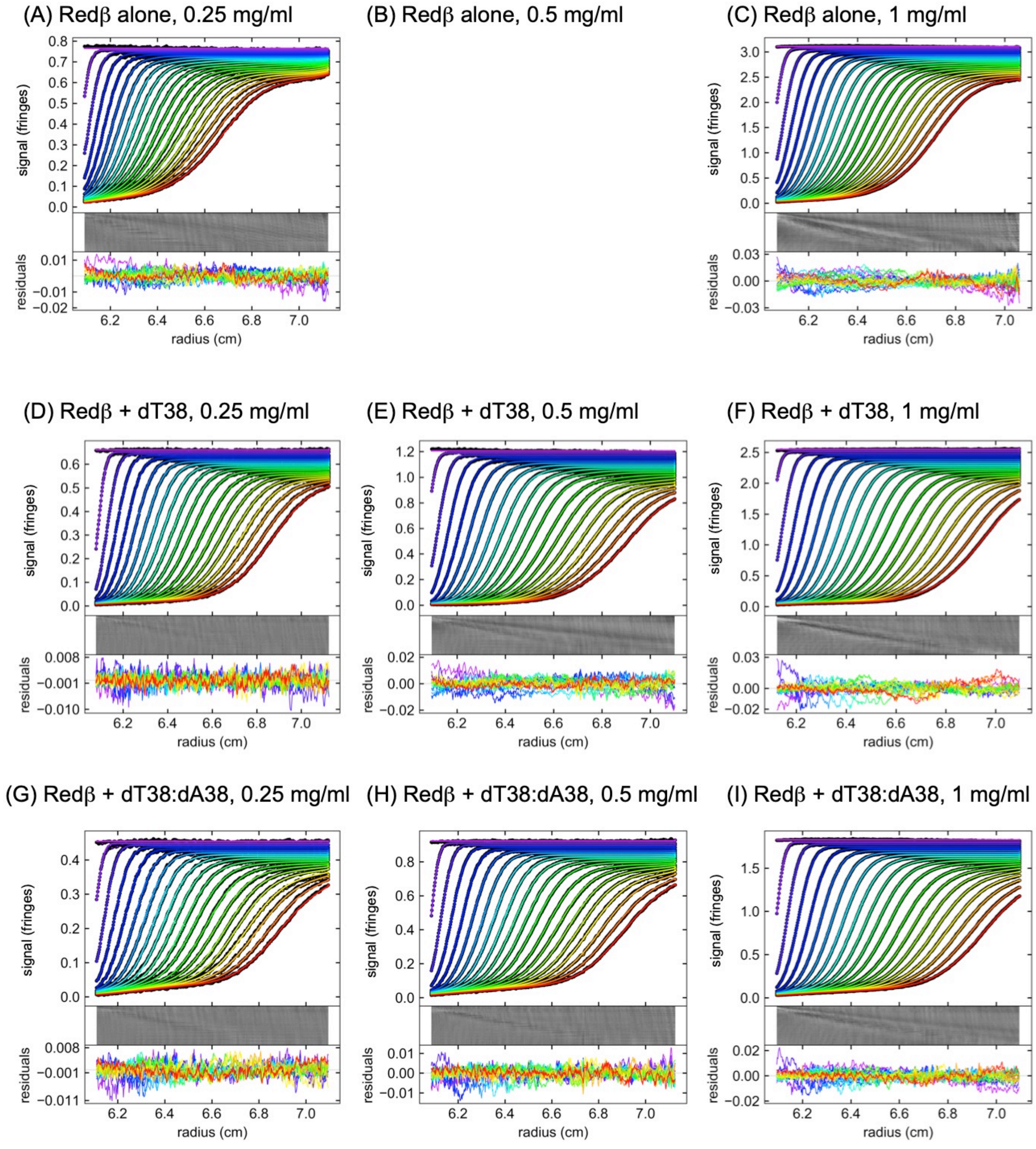

Figure S8. Quality of fits for SV FI data. Fits of the SV FI data for each sample were performed using the *c(s)* analysis in SEDFIT (36) and plotted with GUSSSI (37). Only every 3rd trace (out of 60 total) is shown, with raw data points as circles. The fit from SEDFIT is shown as the smooth line though each trace, as the 2D bitmap in greyscale, and as the residuals at the bottom of each plot. FI data for the sample in panel B were not interpretable.

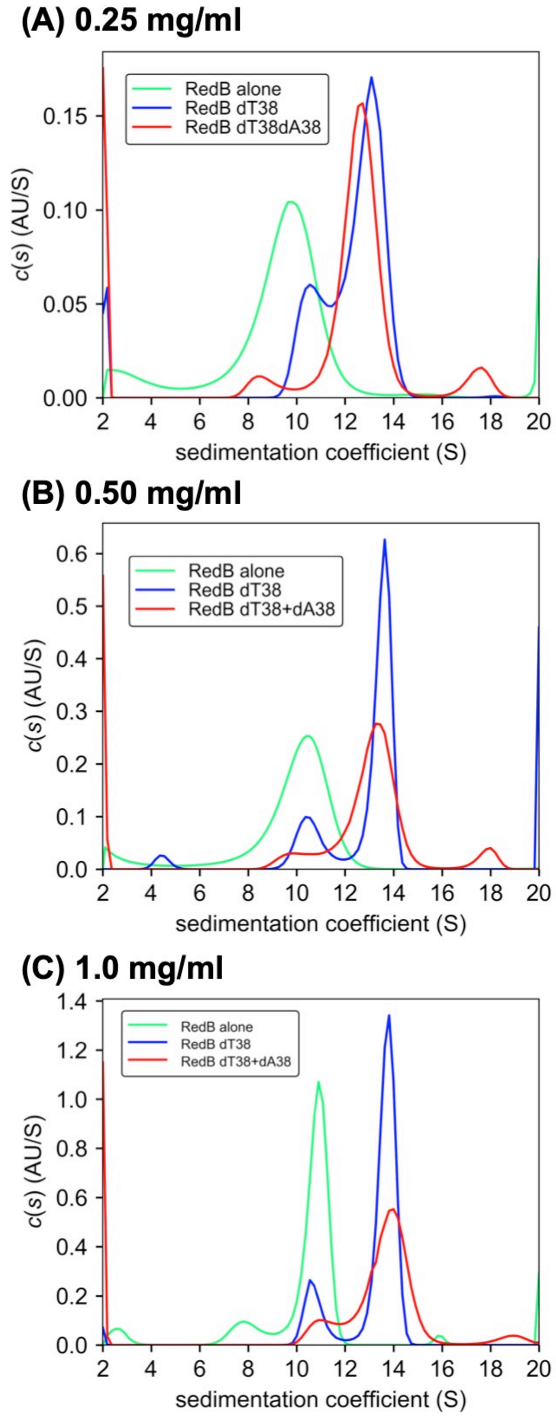

Figure S9. $c(s)$ distributions overlayed for Redβ in three different states (alone and bound to dT38 or dT38:dA38). The distributions were generated from the A280 data using the $c(s)$ model (P = 0.683) in SEDFIT (36), and plotted (non-normalized) with GUSSI (37).

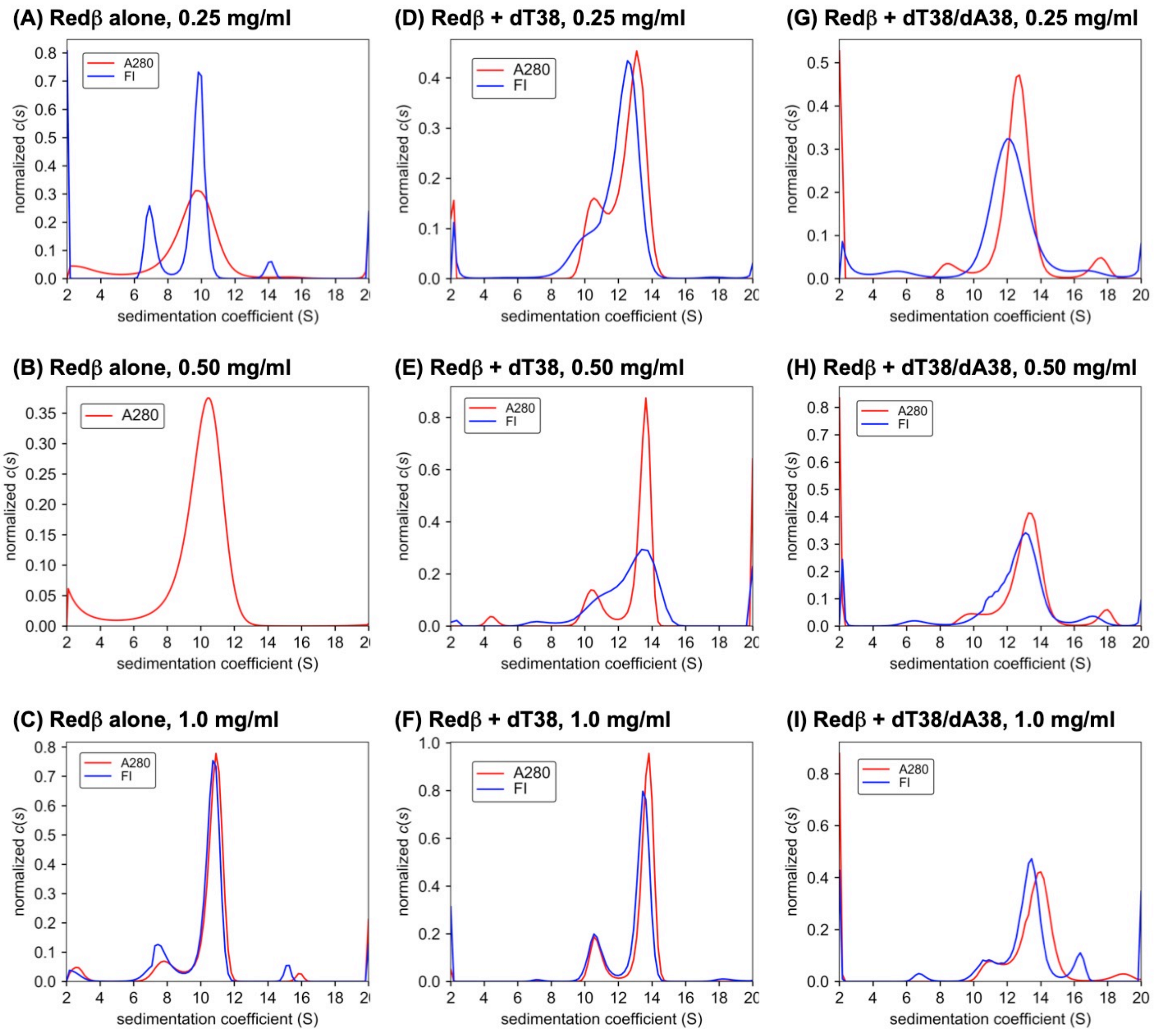

Figure S10. $c(s)$ distributions from each sample overlaid for the A280 and FI data. The $c(s)$ distributions (P = 0.683) were calculated for each of the nine samples in SEDFIT (36) and plotted with GUSSI (37). The overlays show the distributions from the A280 data (red) and the FI data (blue). Samples of Redβ alone (A-C) were from one run, and samples for Redβ + dT38 (D-F) and Redβ + dT38:dA38 (G-I) were from a separate run. The FI data for sample shown in (B) was not interpretable (presumably due to a sample loading error).

(A) Redβ alone

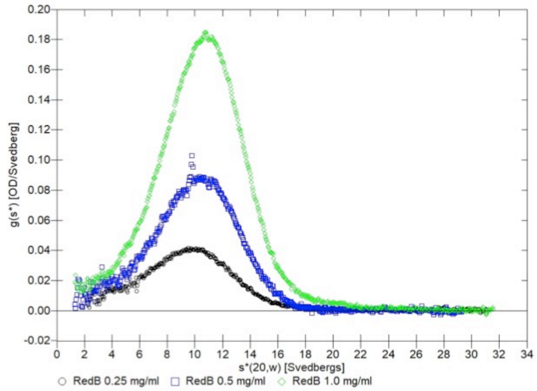

Conc.	***S (w,20)***	***M***	***(n)***
0.25	10.1	232	7.7
0.50	10.6	255	8.5
1.0	10.9	285	9.5

(B) Redβ + dT38

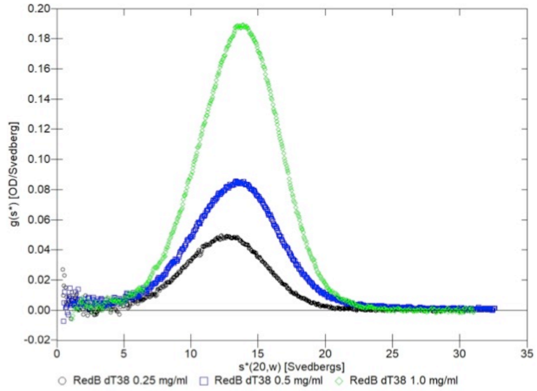

Conc.	***S (w,20)***	***M***	***(n)***
0.25	12.8	295	9.5
0.50	13.6	281	9.0
1.0	13.9	327	10.5

(C) Redβ + dT38:dA38

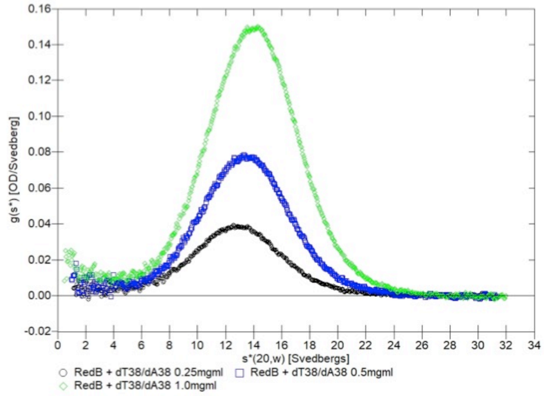

Conc	***S (w,20)***	***M***	**(*n*)**
0.25	13.1	276	8.4
0.50	13.6	275	8.4
1.0	14.2	282	8.6

Figure S11. *g*(*s**) analysis of SV data in DCDT+ (38,39). The apparent sedimentation coefficient distributions were generated from the A280 data for (**A**) Redβ protein alone, (**B**) Redβ + dT38, and (**C**) Redβ + dT38:dA38, at three different concentrations (0.25 mg/mL, black, 0.50 mg/mL blue, and 1.0 mg/mL, green). The tables to the right of each plot give the fitted S(w,20) values, mass (*M* in kDa), and corresponding number of subunits of Redβ (*n*), assuming one copy of the dT38 ssDNA or dT38:dA38 annealed duplex.

Table S2. *g*(*s**) analysis of SV data with DCDT+ (38,39).

	A280			FI		
**Sample (mg/ml)**	***S(w,20)***	***M*(kDa)**	**(*n*)**	***S(w,20)***	***M*(kDa)**	**(*n*)**
Redβ (0.25)	10.1	232	7.7	10.5	275	9.2
Redβ (0.50)	10.6	255	8.5	---	---	---
Redβ (1.0)	10.9	285	9.5	10.3	255	8.5
Redβ + dT38 (0.25)	12.8	295	9.5	13.0	313	10.1
Redβ + dT38 (0.50)	13.6	281	9.0	13.8	303	9.7
Redβ + dT38 (1.0)	13.9	327	10.5	13.1	333	10.7
Redβ + dT38:dA38 (0.25)	13.1	276	8.4	13.2	299	9.2
Redβ + dT38:dA38 (0.50)	13.6	275	8.4	12.7	271	8.3
Redβ + dT38:dA38 (1.0)	14.2	282	8.6	14.4	314	9.7

The data show the parameters from the *g*(*s**) analysis of nine samples, from both A280 and FI data, including the fitted *S(w,20)*, mass (*M*) in kDa, and number of subunits of Redβ (*n*), assuming each oligomer contains one copy of dT38 ssDNA or dT38:dA38 annealed duplex.

Table S3. Expected mass and mass determined by nMS, for each component. All masses were determined by UniDec (45), with sampling every 1 Da.

Component	Expected (Da)	Determined (Da)
Redβ	29,969	29,970
T38	11,498	11,498
A38	11,840	11,840
38NC1+	11,748	11,747
38NC1-	11,609	11,608
38NC3+	11,732	11,731
40NC3-	12,242	12,241
83-	25,432	25,432
83+	25,720	25,720
87+	26,956	26,954
87NC	26,757	26,756

Table S4. Sequences of each component used in the nMS analyses.

Component	Sequence
Redβ	GSHMSTALATLAGKLAERVGMDSVDPQELITTLRQTAFKGDASDAQFIALLIVANQYGLNPWTKE<br>IYAFPDKQNGIVPVVGVDGWSRIINENQQFDGMDFEQDNESCTCRIYRKDRNHPICVTEWMDECR<br>REPFKTREGREITGPWQSHPKRMLRHKAMIQCARLAFGFAGIYDKDEAERIVENTAYTAERQPER<br>DITPVNDETMQEINTLLIALDKTWDDDLLPLCSQIFRRDIRASSELTQAEAVKALGFLKQKAAEQ<br>KVAA
T38	5′-TTTTTTTTTTTTTTTTTTTTTTTTTTTTTTTTTTTTTT-3′
A38	5′-AAAAAAAAAAAAAAAAAAAAAAAAAAAAAAAAAAAAAA-3′
38NC1+	5′-AGACACAGTAAACGGACGCTAGATGCAACGAGATCCGA-3′
38NC1-	5′-TCGGATCTCGTTGCATCTAGCGTCCGTTTACTGTGTCT-3′
38NC3+	5′-GACGCACAATGACAGATAACACAATGGCGAGATAACGC-3′
40NC3-	5′-GCGTTATCTCGCCATTGTGTTATCTGTCATTGTGCGTCGC-3′
83-	5′TTGCATATTTAAAACATGTTGAGCTACAGCACCAGATTCAGCAATTAAGCTCTAAGCCATCCG<br>CAAAAATGACCTCTTATCAA-3′
83+	5′TTGATAAGAGGTCATTTTTGCGGATGGCTTAGAGCTTAATTGCTGAATCTGGTGCTGTAGCTC<br>AACATGTTTTAAATATGCAA-3′
87+	5′TTGATAAGAGGTCATTTTTGCGGATGGCTTAGAGCTTAATTGCTGAATCTGGTGCTGTAGCTC<br>AACATGTTTTAAATATGCAATGAC-3′
87NC	5′GGTTATGTTTCAAGCGCACCTAATGCTAGAGTTATAGGTGGCAACTGCGACTCACGCCGCTAA<br>TCAGGCCGCACTTCCATAGCTCGA-3′

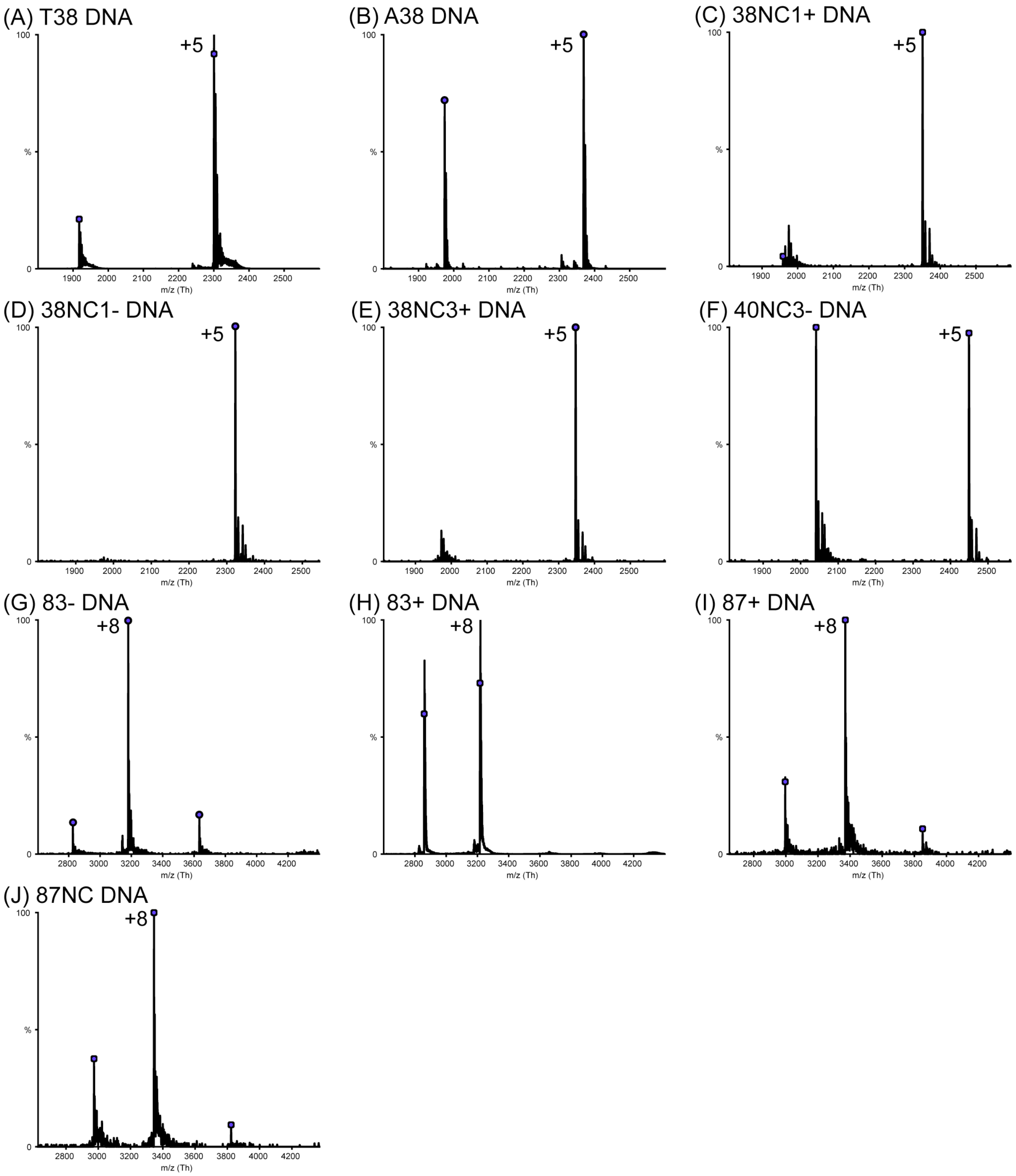

Figure S12. Mass spectra of each DNA stock. (**A**) T38, (**B**) A38, and (**H**) 83+ were collected at 30 μM nt using nano ESI in 50 mM ammonium acetate. (**C**) 38NC1+, (**D**) 38NC1-, (**E**) 38NC3+, (**F**) 40NC3-, (**G**) 83-, (**I**) 87+, and (**J**) 87NC were collected using a rapid online buffer exchange method (44). 25 picomoles of each component were injected and eluted with a 200 mM ammonium acetate solution. No large amount of impurities was detected, and the major species matched the expected molecular weights (Table S3). The DNA were routinely checked.

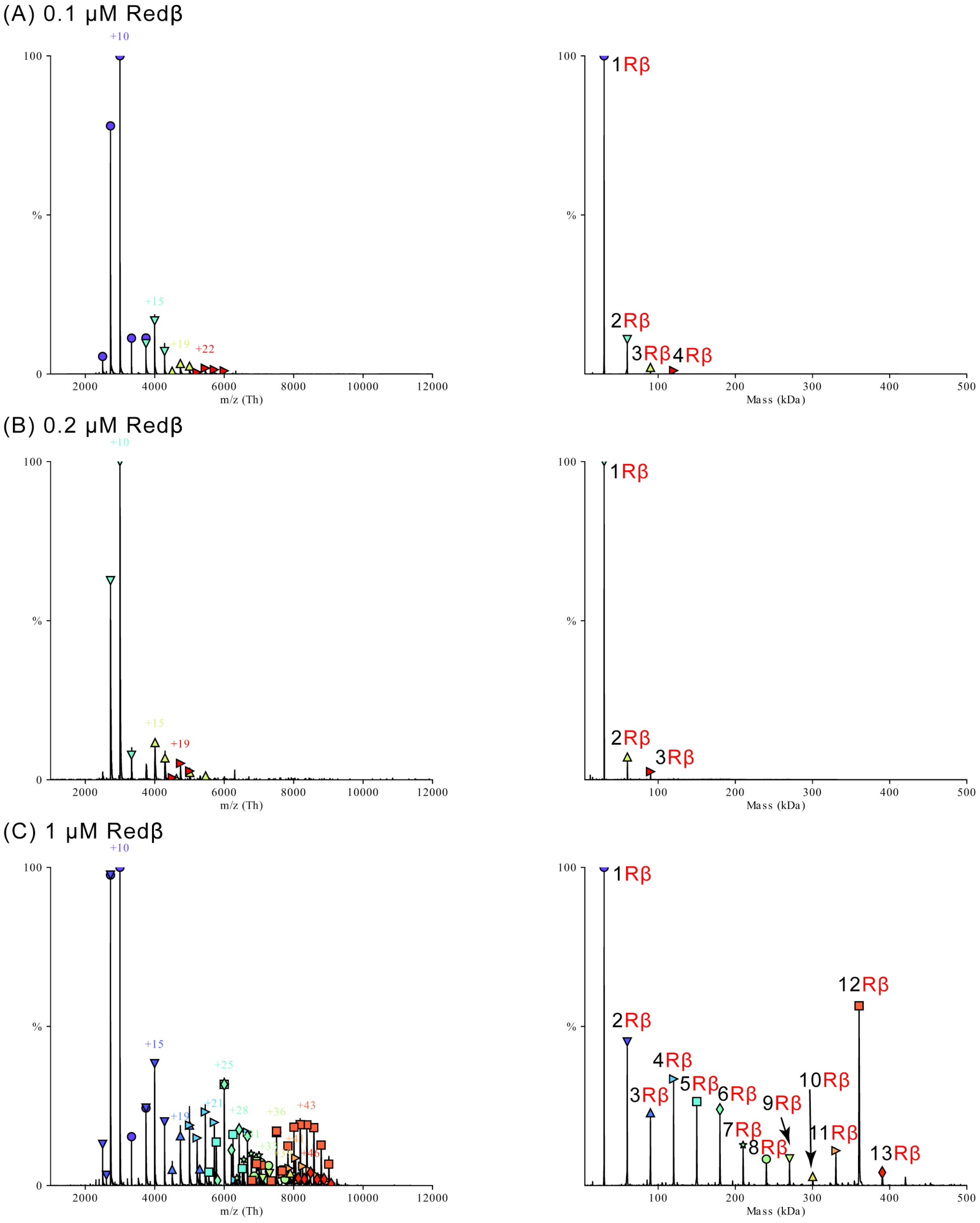

(A) 0.1 µM Redβ
+10
+15
+19
+22
1Rβ
2Rβ
3Rβ
4Rβ
m/z (Th)
Mass (kDa)
(B) 0.2 µM Redβ
+10
+15
+19
1Rβ
2Rβ
3Rβ
m/z (Th)
Mass (kDa)
(C) 1 µM Redβ
+10
+15
+19
+21
+25
+28
+36
+43
1Rβ
2Rβ
3Rβ
4Rβ
5Rβ
6Rβ
7Rβ
8Rβ
9Rβ
10Rβ
11Rβ
12Rβ
13Rβ
m/z (Th)
Mass (kDa)

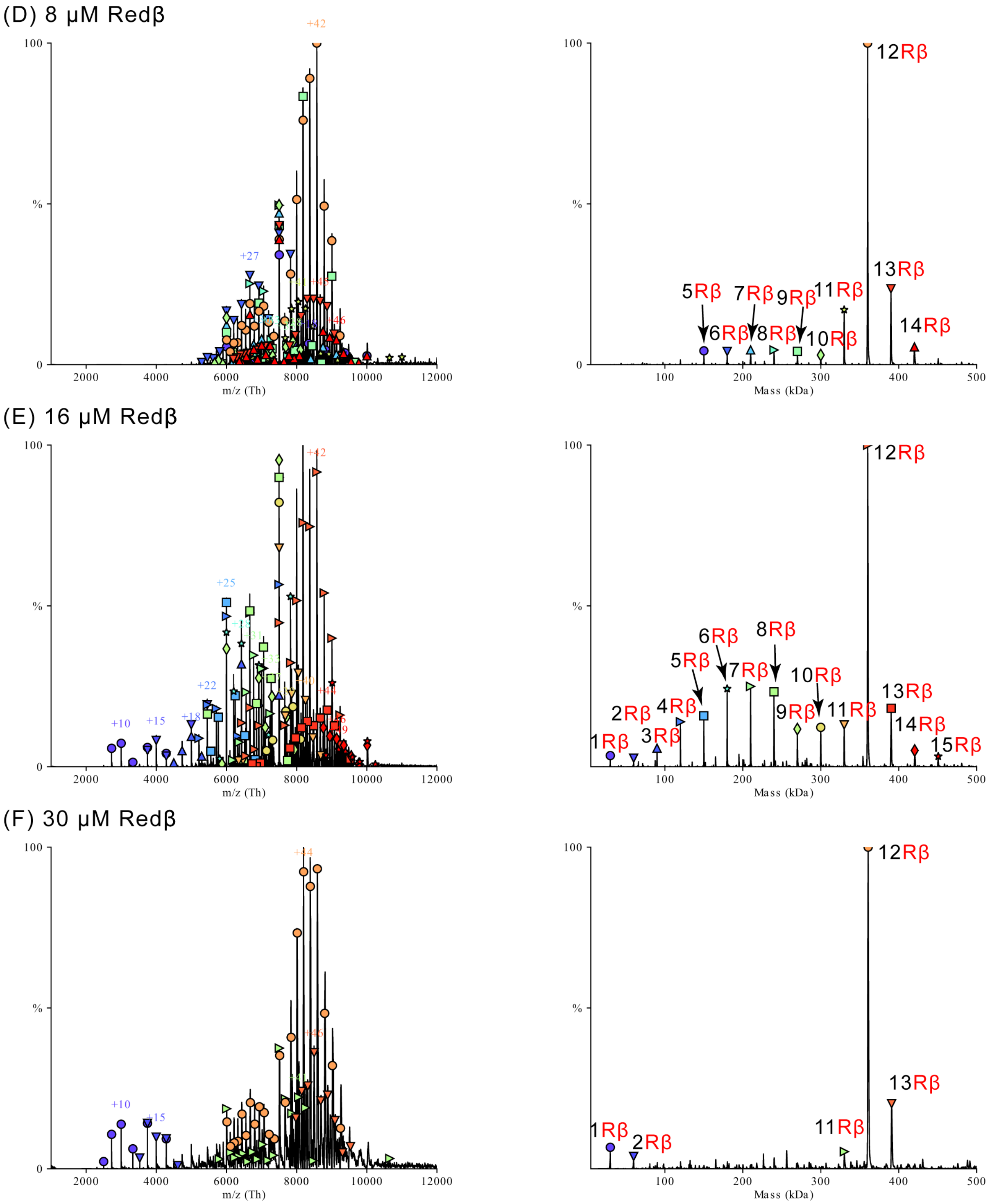

Figure S13. Mass spectra (left) and zero-charge mass spectra (right) of Redβ alone at (**A**) 0.1 μM, (**B**) 0.2 μM, (**C**) 1 μM, (**D**) 8 μM, (**E**) 16 μM, and (**F**) 30 μM. All spectra were collected at 60 V of HCD.

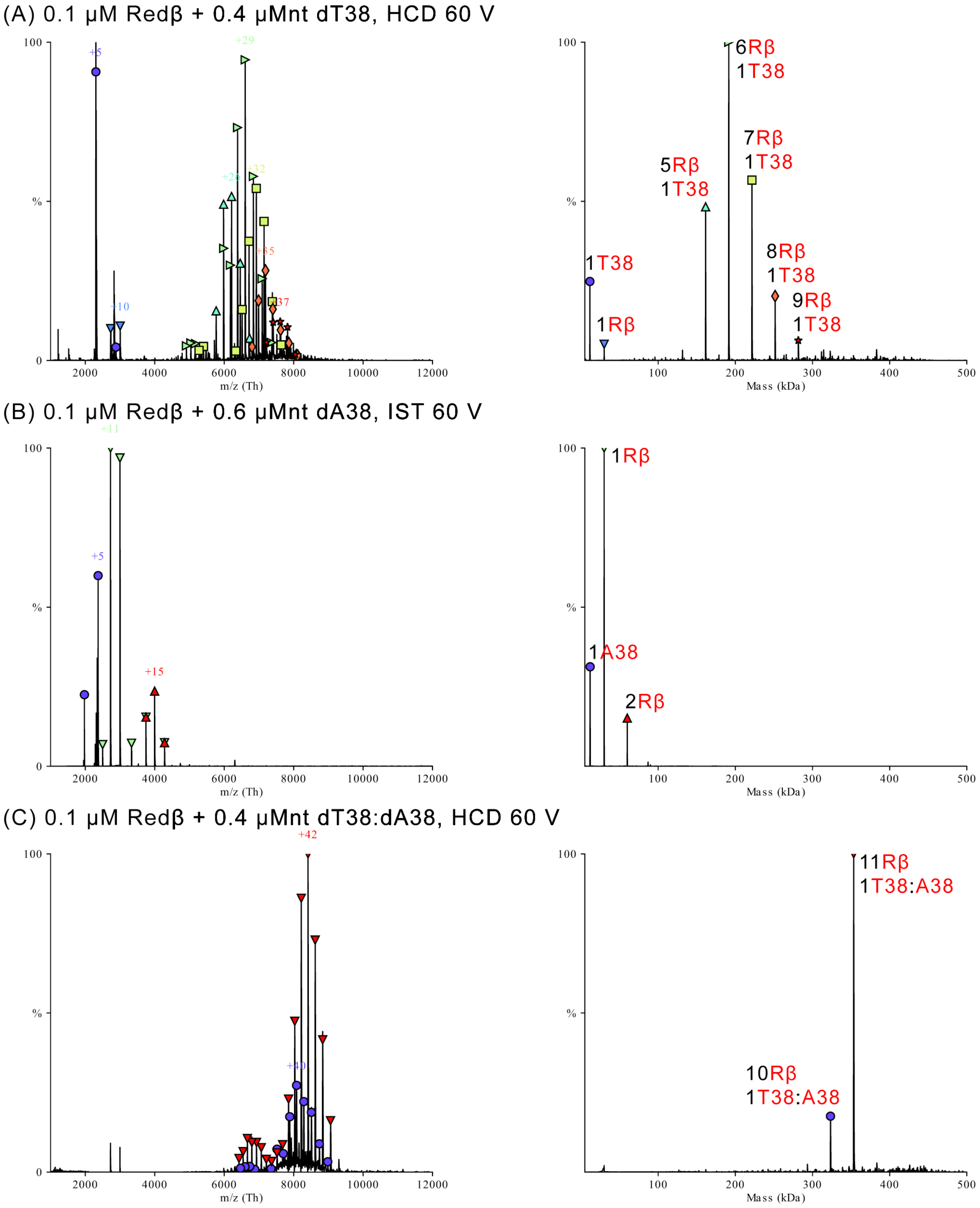
(A) 0.1 µM Redβ + 0.4 µMnt dT38, HCD 60 V
+5
+10
+29
+26
+32
+35
+37
m/z (Th)
1T38
1Rβ
5Rβ 1T38
6Rβ 1T38
7Rβ 1T38
8Rβ 1T38
9Rβ 1T38
Mass (kDa)
(B) 0.1 µM Redβ + 0.6 µMnt dA38, IST 60 V
+11
+5
+15
m/z (Th)
1Rβ
1A38
2Rβ
Mass (kDa)
(C) 0.1 µM Redβ + 0.4 µMnt dT38:dA38, HCD 60 V
+42
+40
m/z (Th)
11Rβ 1T38:A38
10Rβ 1T38:A38
Mass (kDa)

(D) 1 µM Redβ + 4 µMnt dT38, HCD 60 V

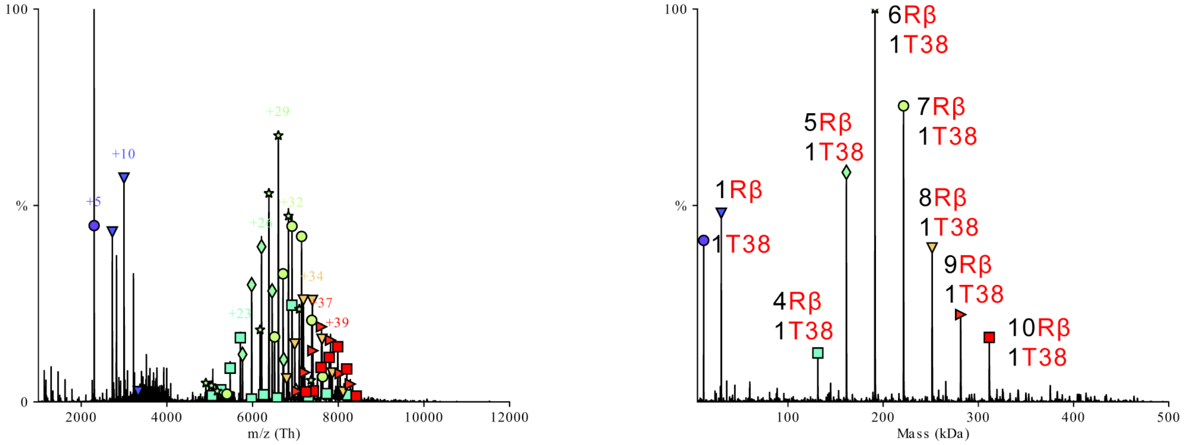

(E) 1 µM Redβ + 4 µMnt dA38, IST 60 V

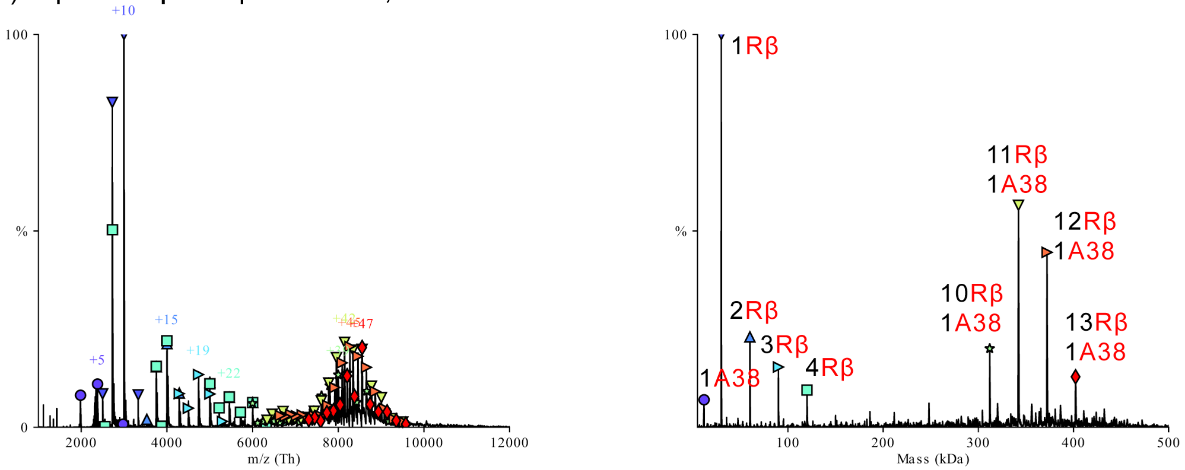

(F) 1 µM Redβ + 4 µMnt dT38:dA38, HCD 60 V

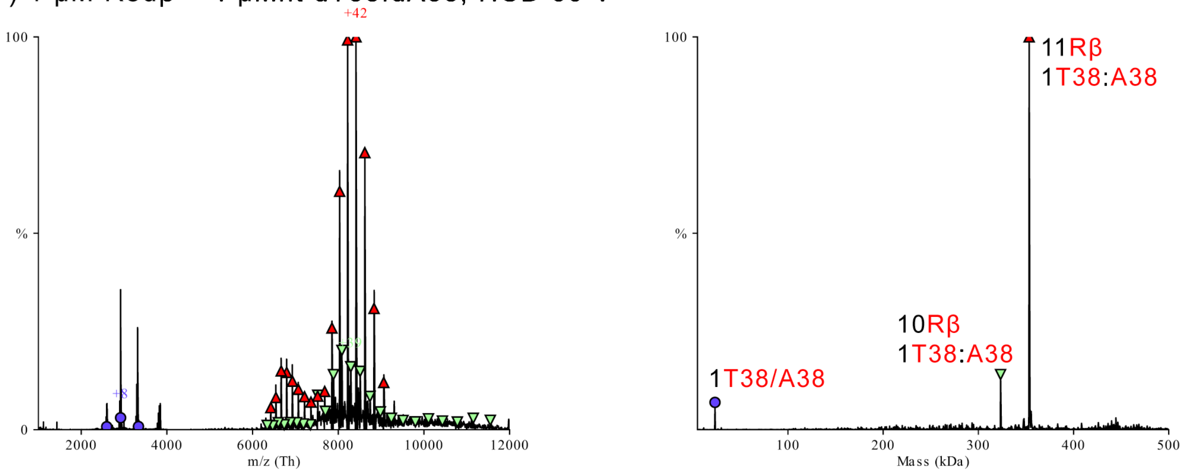

(G) 8 μM Redβ + 32 μMnt dT38, HCD 60 V

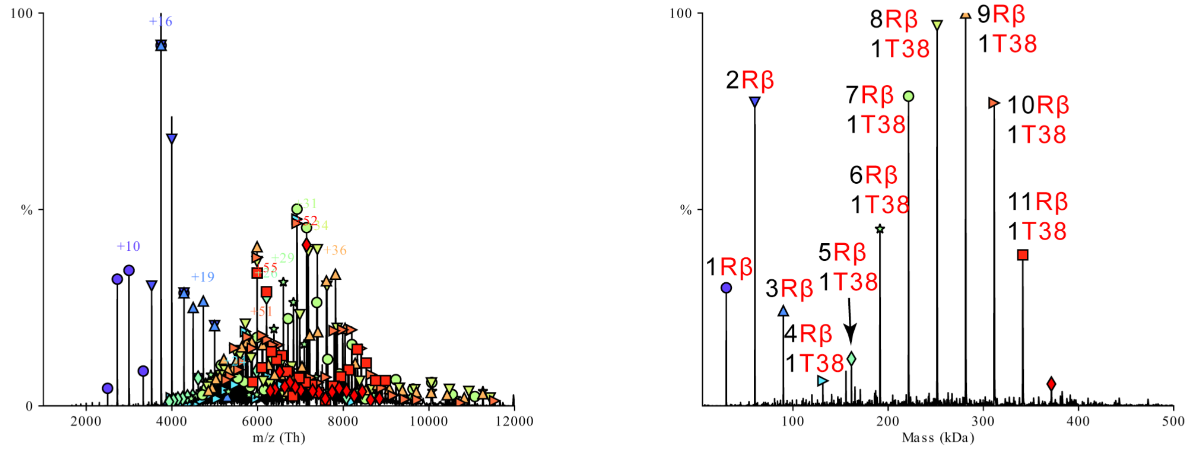

(H) 8 μM Redβ + 32 μMnt dA38, HCD 60 V

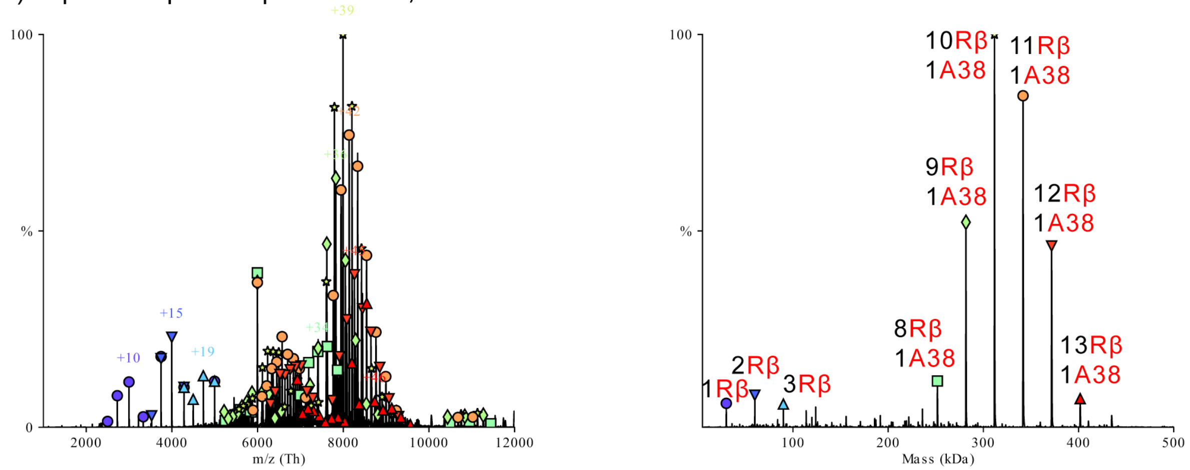

(I) 8 μM Redβ + 32 μMnt dT38:dA38, HCD 60 V

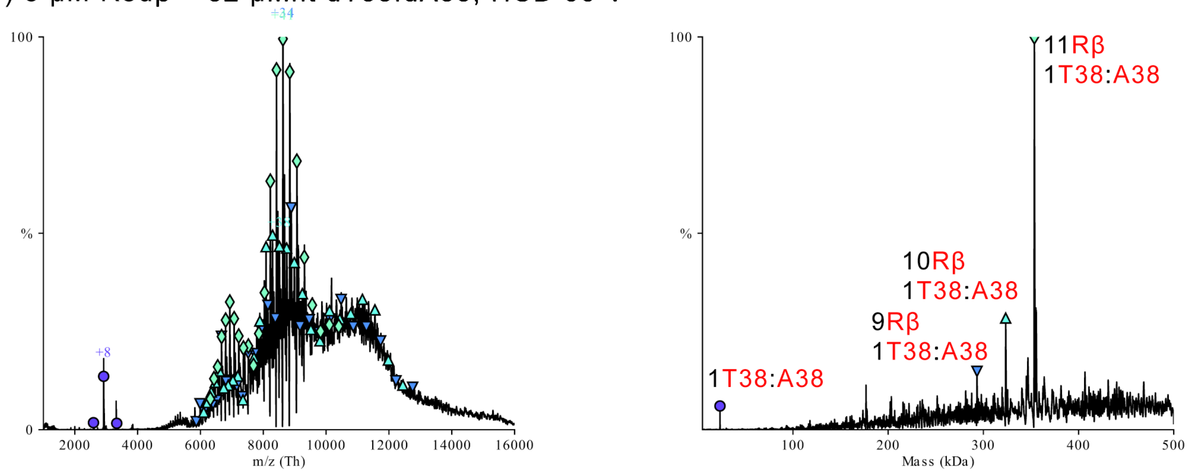

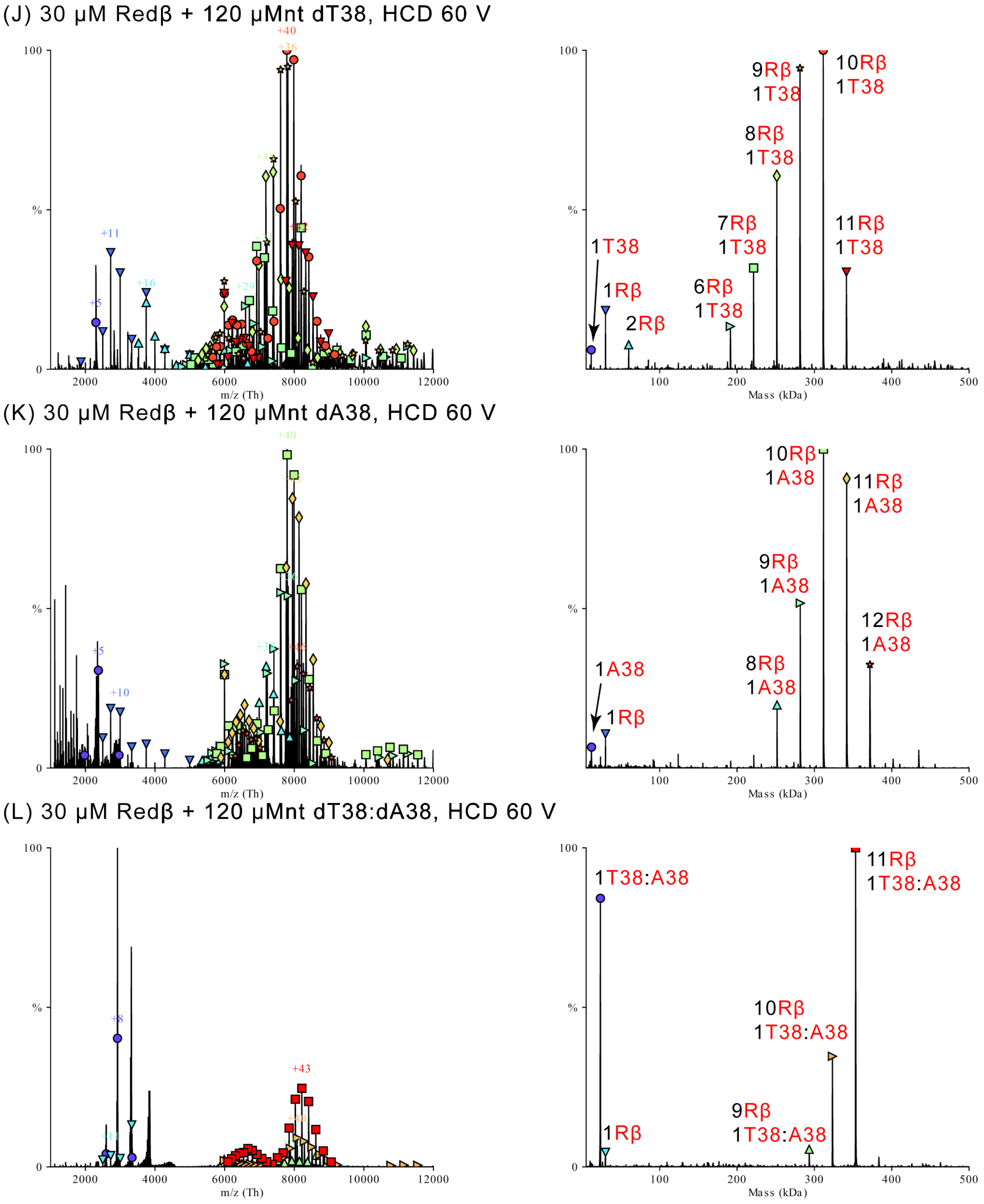

Figure S14. Mass spectra (left) and zero-charge mass spectra (right) of Redβ plus 38-mer DNA under the following conditions: (**A**) 0.1 μM Redβ + 0.4 μM nucleotides (μM nt) dT38 at HCD 60 V, (**B**) 0.1 μM Redβ + 0.6 μM nt dA38 at IST 60 V, (**C**) 0.1 μM Redβ + 0.4 μM nt dT38:dA38 at

HCD 60 V, (**D**) 1 μM Redβ + 4 μM nt dT38 at HCD 60 V, (**E**) 1 μM Redβ + 4 μM nt dA38 at IST 60 V, (**F**) 1 μM Redβ + 4 μM nt dT38:dA38 at HCD 60 V, (**G**) 8 μM Redβ + 32 μM nt dT38 at HCD 60 V, (**H**) 8 μM Redβ + 32 μM nt dA38 at HCD 60 V, (**I**) 8 μM Redβ + 32 μM nt dT38:dA38 at HCD 60 V, (**J**) 30 μM Redβ + 120 μM nt dT38 at HCD 60 V, (**K**) 30 μM Redβ + 120 μM nt dA38 at HCD 60 V, (**L**) 30 μM Redβ + 120 μM nt dT38:dA38 at HCD 60 V.

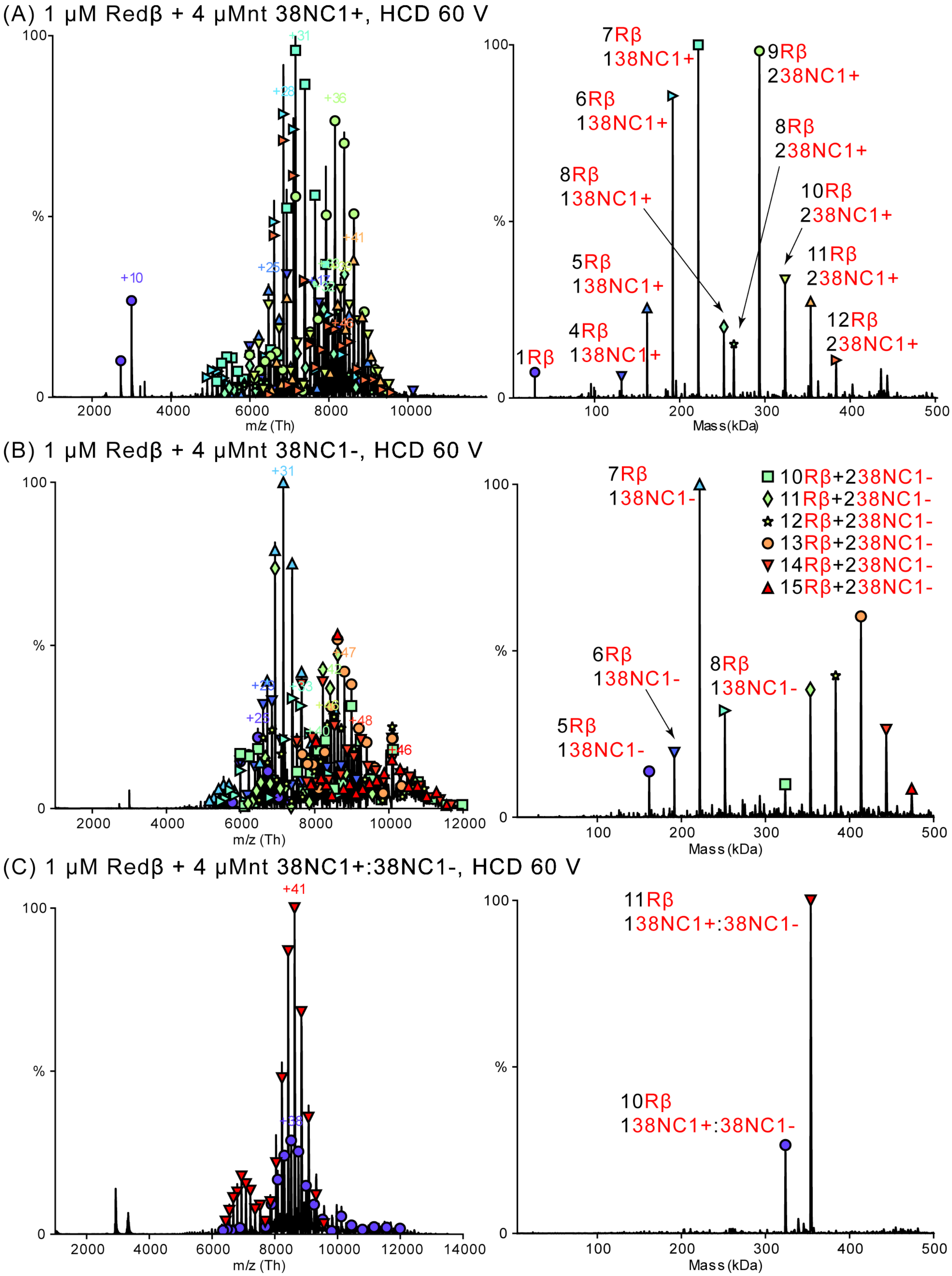

(A) 1 μM Redβ + 4 μMnt 38NC1+, HCD 60 V
+10
+28
+31
+36
+41
m/z (Th)
Mass (kDa)
1Rβ
4Rβ 138NC1+
5Rβ 138NC1+
6Rβ 138NC1+
7Rβ 138NC1+
8Rβ 138NC1+
9Rβ 238NC1+
8Rβ 238NC1+
10Rβ 238NC1+
11Rβ 238NC1+
12Rβ 238NC1+
(B) 1 μM Redβ + 4 μMnt 38NC1-, HCD 60 V
+31
+47
+48
+46
m/z (Th)
Mass (kDa)
7Rβ 138NC1-
10Rβ+238NC1-
11Rβ+238NC1-
12Rβ+238NC1-
13Rβ+238NC1-
14Rβ+238NC1-
15Rβ+238NC1-
6Rβ 138NC1-
8Rβ 138NC1-
5Rβ 138NC1-
(C) 1 μM Redβ + 4 μMnt 38NC1+:38NC1-, HCD 60 V
+41
m/z (Th)
Mass (kDa)
11Rβ 138NC1+:38NC1-
10Rβ 138NC1+:38NC1-

(D) 1 μM Redβ + 4 μMnt 38NC3+, HCD 60 V

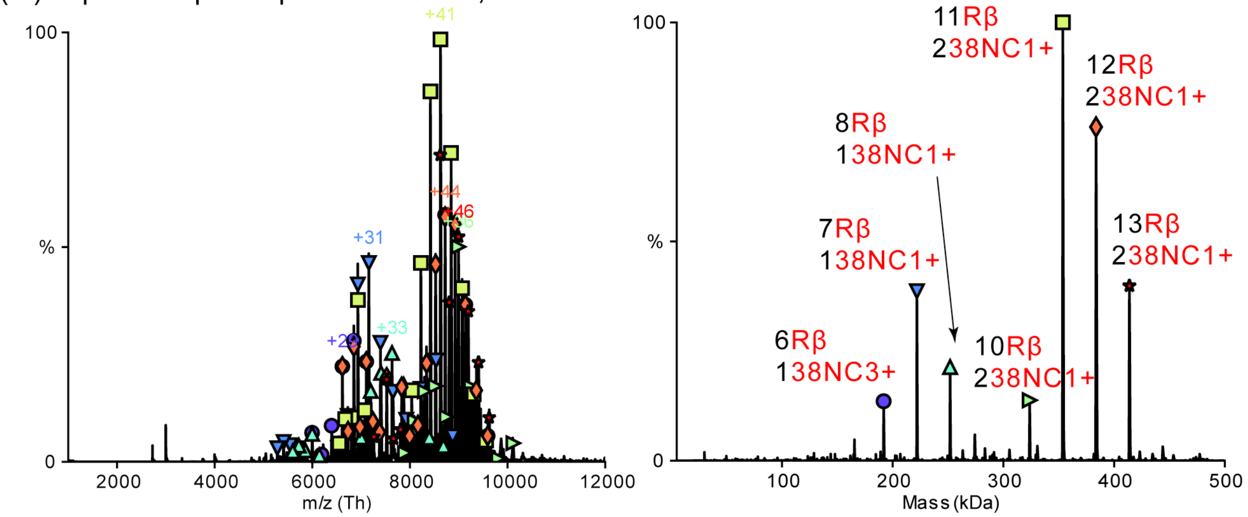

(E) 1 μM Redβ + 4 μMnt 40NC3-, HCD 60 V

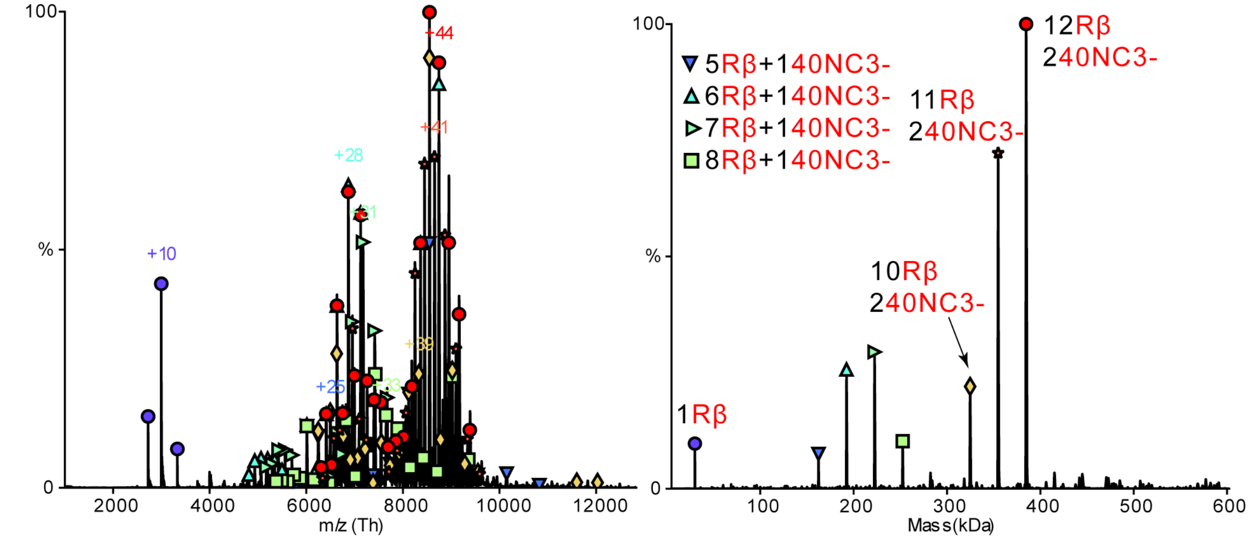

(F) 1 μM Redβ + 4 μMnt 38NC3+:40NC3-, HCD 60 V

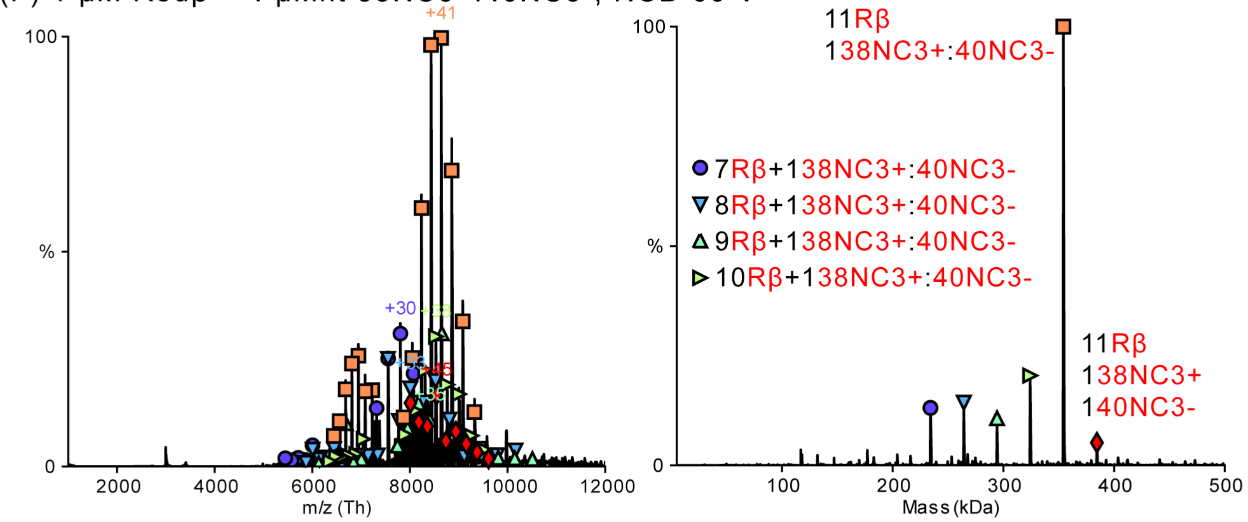

(G) 1 μM Redβ + 4 μMnt T38:38NC1-, HCD 60 V

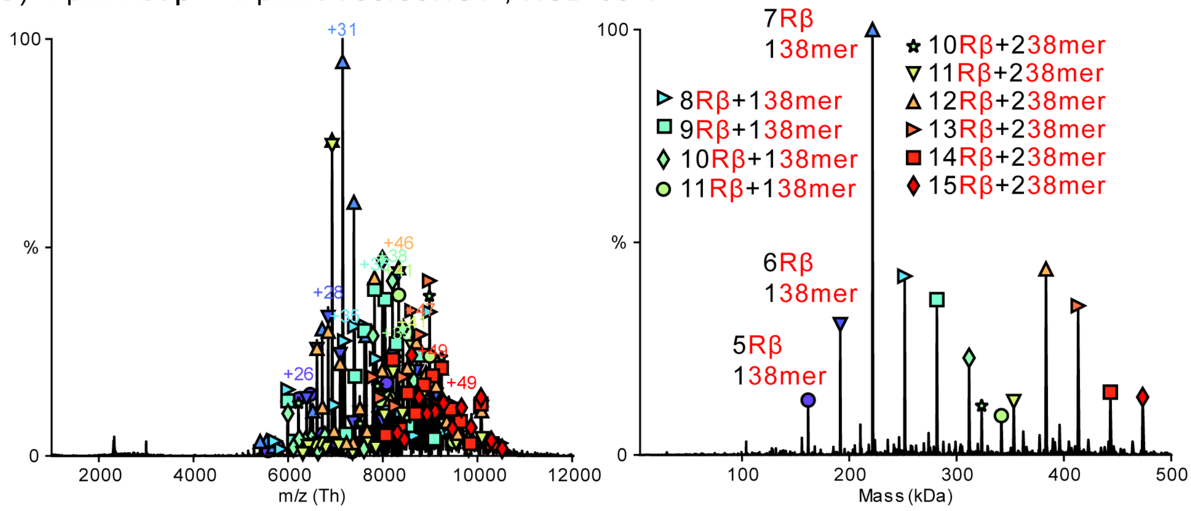

(H) 1 μM Redβ + 4 μMnt T38:40NC3-, HCD 60 V

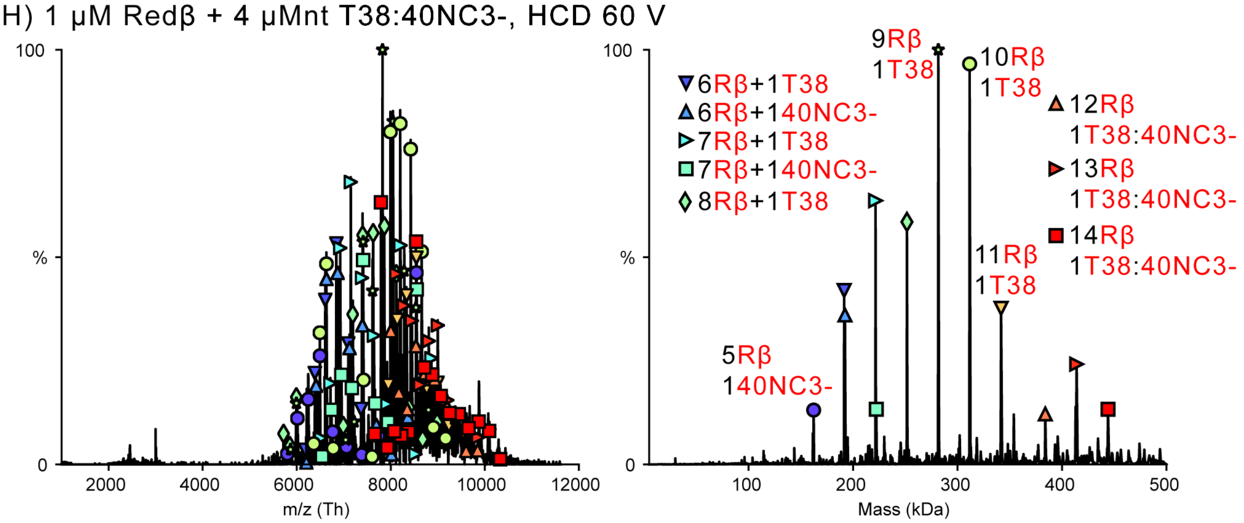

Figure S15. Mass spectra (left) and zero-charge mass spectra (right) of Redβ plus 38-mer DNA under the following conditions: (**A**) 1 μM Redβ + 4 μM nt 38NC1+, (**B**) 1 μM Redβ + 4 μM nt 38NC1-, (**C**) 1 μM Redβ + 4 μM nt 38NC1+:38NC1-, (**D**) 1 μM Redβ + 4 μM nt 38NC3+, (**E**) 1 μM Redβ + 4 μM nt (adjusted) 40NC3-, (**F**) 1 μM Redβ + 4 μM nt (adjusted) 38NC3+:40NC3-, (**G**) 1 μM Redβ + 4 μM nt T38:40NC1-, (**H**) 1 μM Redβ + 4 μM nt (adjusted) T38:40NC3- at HCD 60 V. All spectra were collected at 60 V of HCD. Spectra shown are representative of multiple spectra collected to obtain the average relative intensities shown in the bar charts of Figure S16.

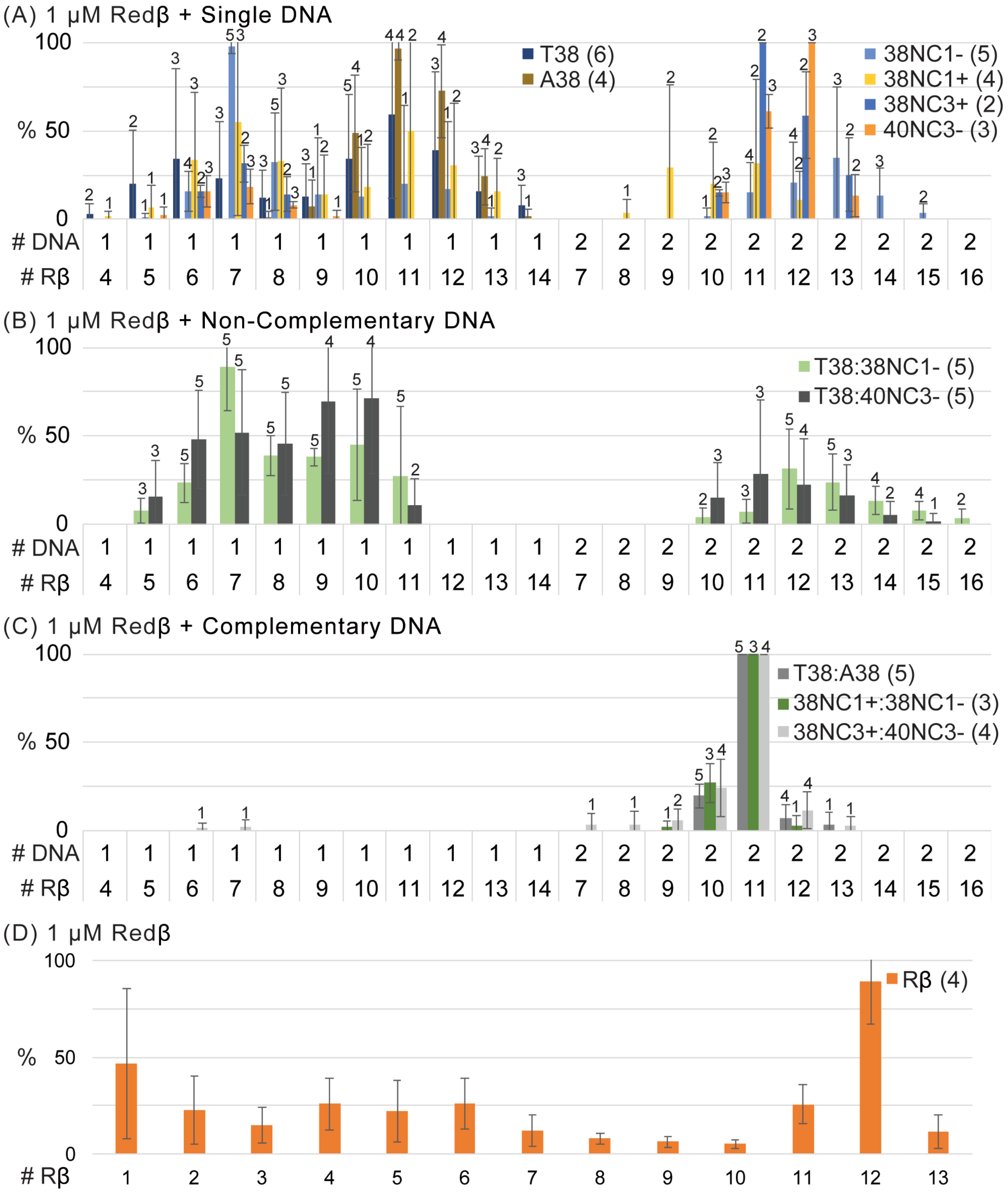

Figure S16. Averaged bar charts of the distribution of oligomers observed in the absence or presence of different combinations of 38-mer DNA. The plots give the average relative intensity of each oligomeric species observed when Redβ was mixed with (**A**) a single DNA, (**B**) two non-complementary DNAs added sequentially, (**C**) two complementary DNAs added sequentially, or

(**D**) no DNA. The error bars indicate the standard deviations, and the number in parentheses for each sample gives the number of deconvoluted spectra that was used for averaging. The number above each bar indicates the number of spectra in which the specified oligomer was observed. If a mass spectrum did not contain a given oligomer, then its relative intensity was recorded as 0 in the calculations for the average and standard deviation. Notice that the variability in the oligomers observed when Redβ was mixed with a single DNA (A) or with two non-complementary DNAs (B) is large, whereas the variability is much smaller for the complexes formed when Redβ was mixed with two complementary DNAs (C). For the data in the figure, all mass spectra were collected with a collision voltage of 60, which allowed for the peaks in the spectra to be resolvable, but occasionally resulted in complex dissociation that when calculable attributed to less than 5% of the species intensity.

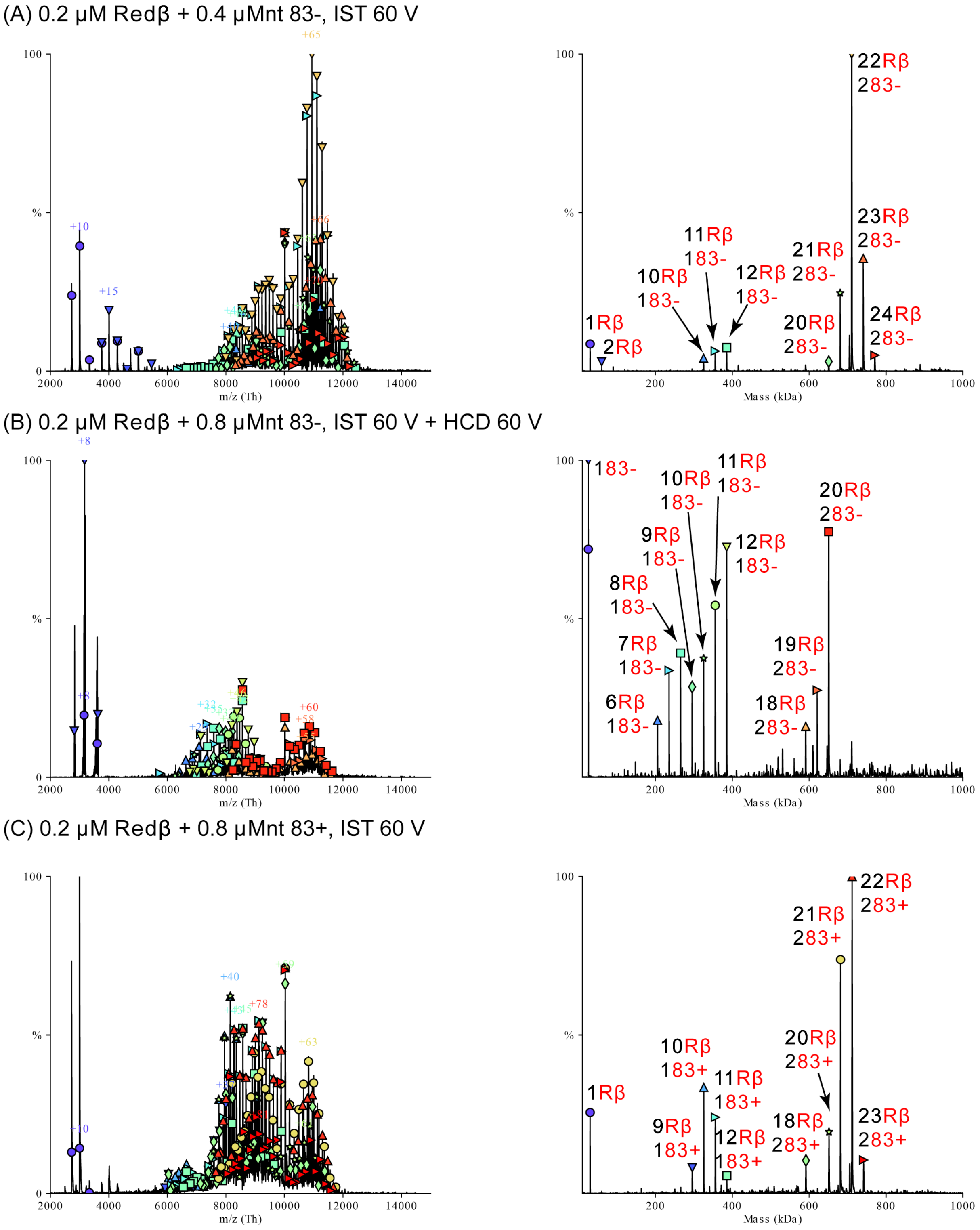
(A) 0.2 μM Redβ + 0.4 μMnt 83-, IST 60 V
m/z (Th)
Mass (kDa)
1Rβ
2Rβ
10Rβ 183-
11Rβ 183-
12Rβ 183-
20Rβ 283-
21Rβ 283-
22Rβ 283-
23Rβ 283-
24Rβ 283-
(B) 0.2 μM Redβ + 0.8 μMnt 83-, IST 60 V + HCD 60 V
m/z (Th)
Mass (kDa)
183-
6Rβ 183-
7Rβ 183-
8Rβ 183-
9Rβ 183-
10Rβ 183-
11Rβ 183-
12Rβ 183-
18Rβ 283-
19Rβ 283-
20Rβ 283-
(C) 0.2 μM Redβ + 0.8 μMnt 83+, IST 60 V
m/z (Th)
Mass (kDa)
1Rβ
9Rβ 183+
10Rβ 183+
11Rβ 183+
12Rβ 183+
18Rβ 283+
20Rβ 283+
21Rβ 283+
22Rβ 283+
23Rβ 283+

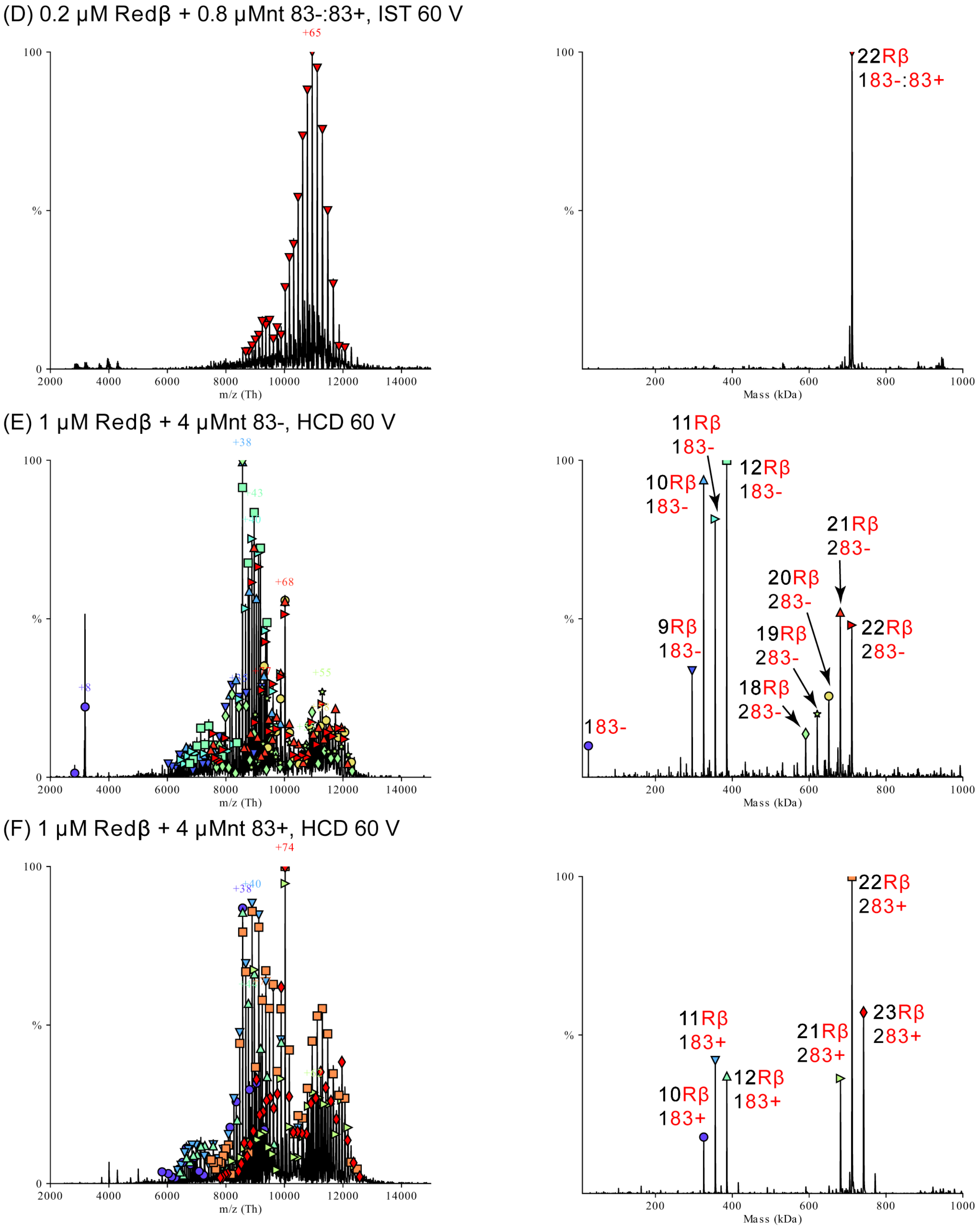
(D) 0.2 µM Redβ + 0.8 µMnt 83-:83+, IST 60 V
+65
100
%
0
2000
4000
6000
8000
10000
12000
14000
m/z (Th)
22Rβ
183-:83+
200
400
600
800
1000
Mass (kDa)
(E) 1 µM Redβ + 4 µMnt 83-, HCD 60 V
+38
+43
+68
+55
+8
11Rβ
183-
10Rβ
183-
12Rβ
183-
21Rβ
283-
20Rβ
283-
9Rβ
183-
19Rβ
283-
22Rβ
283-
18Rβ
283-
183-
(F) 1 µM Redβ + 4 µMnt 83+, HCD 60 V
+74
+38
+40
22Rβ
283+
11Rβ
183+
21Rβ
283+
23Rβ
283+
12Rβ
183+
10Rβ
183+

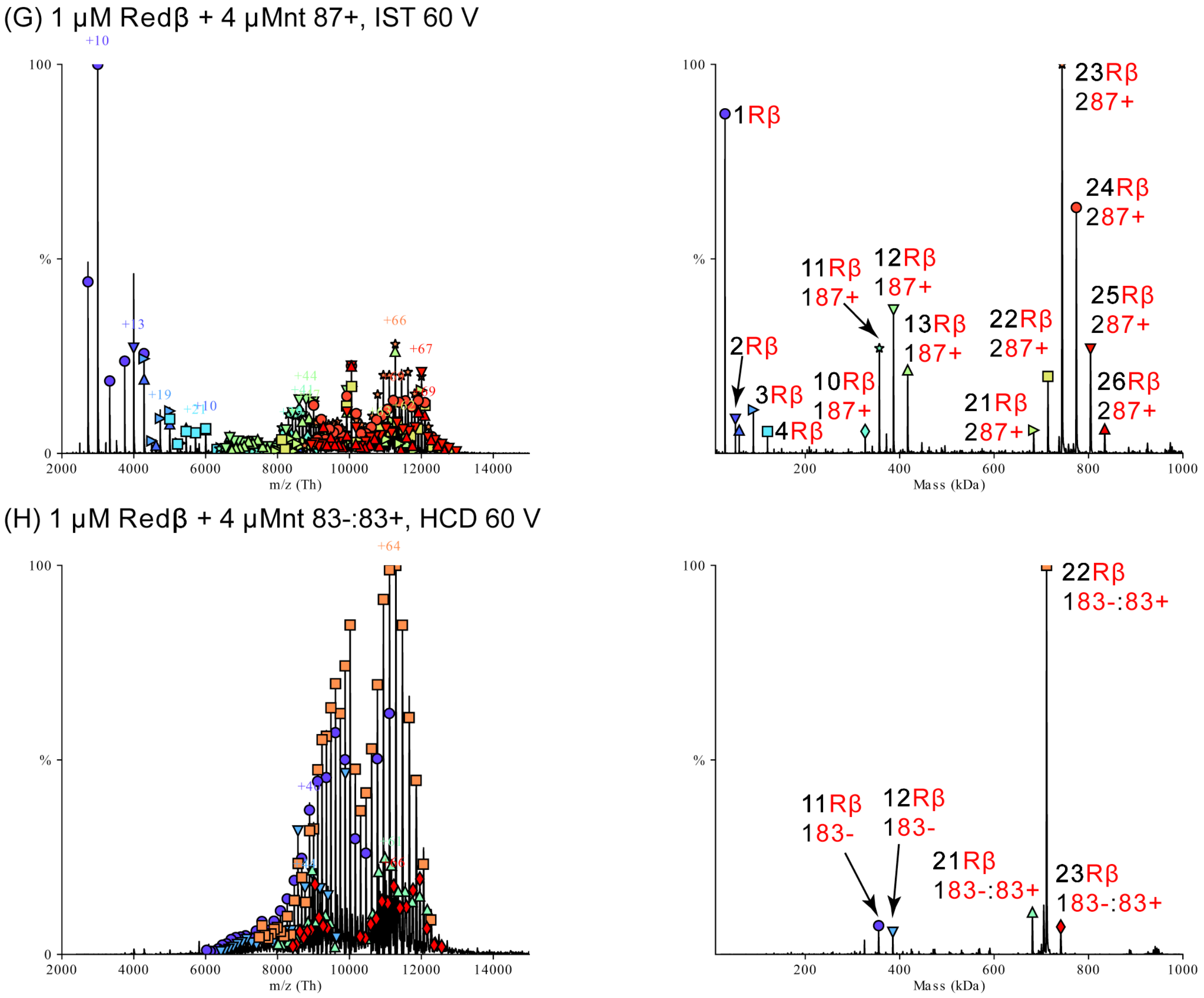

Figure S17. Mass spectra (left) and zero-charge mass spectra (right) of Redβ plus 83-mer and/ or 87-mer DNA under the following conditions: (**A**) 0.2 μM Redβ + 0.4 μM nt 83- at IST 60 V, (**B**) 0.2 μM Redβ + 0.8 μM nt 83- at IST 60 V and HCD 60 V, (**C**) 0.2 μM Redβ + 0.8 μM nt 83+ at IST 60 V, (**D**) 0.2 μM Redβ + 0.8 μM nt 83-:83+ at IST 60 V, (**E**) 1 μM Redβ + 4 μM nt 83- at HCD 60 V, (**F**) 1 μM Redβ + 4 μM nt 83+ at HCD 60 V, (**G**) 1 μM Redβ + 4 μM nt (adjusted) 87+ at IST 60 V, (**H**) 1 μM Redβ + 4 μM nt 83-:83+ at HCD 60 V.

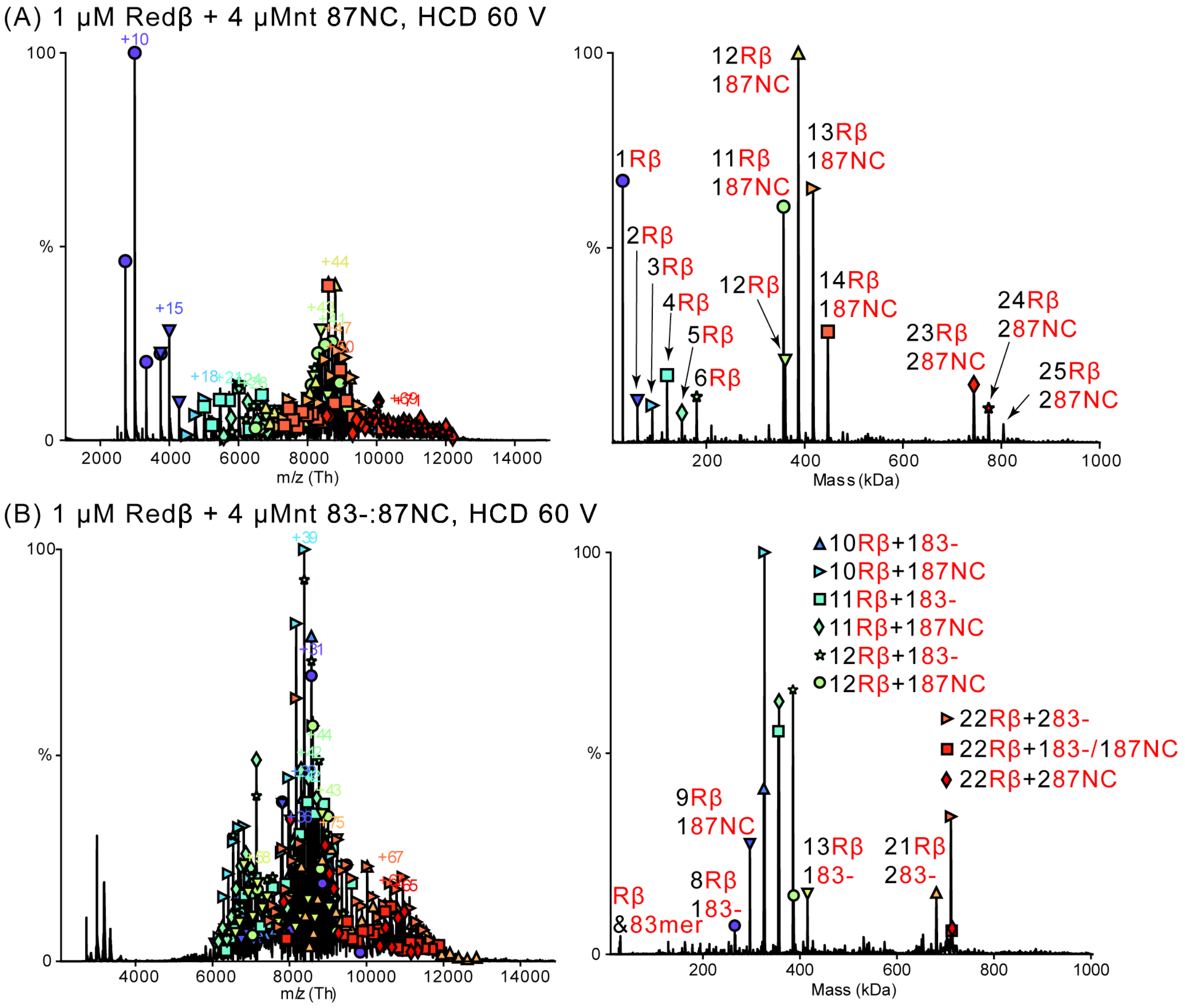

Figure S18. Mass spectra (left) and zero-charge mass spectra (right) of Redβ plus 83-mer and/or 87-mer DNA under the following conditions: (**A**) 1 µM Redβ + 4 µM nt 87NC at HCD 60 V, (**B**) 1 µM Redβ + 4 µM nt 83-:87NC at HCD 60 V.

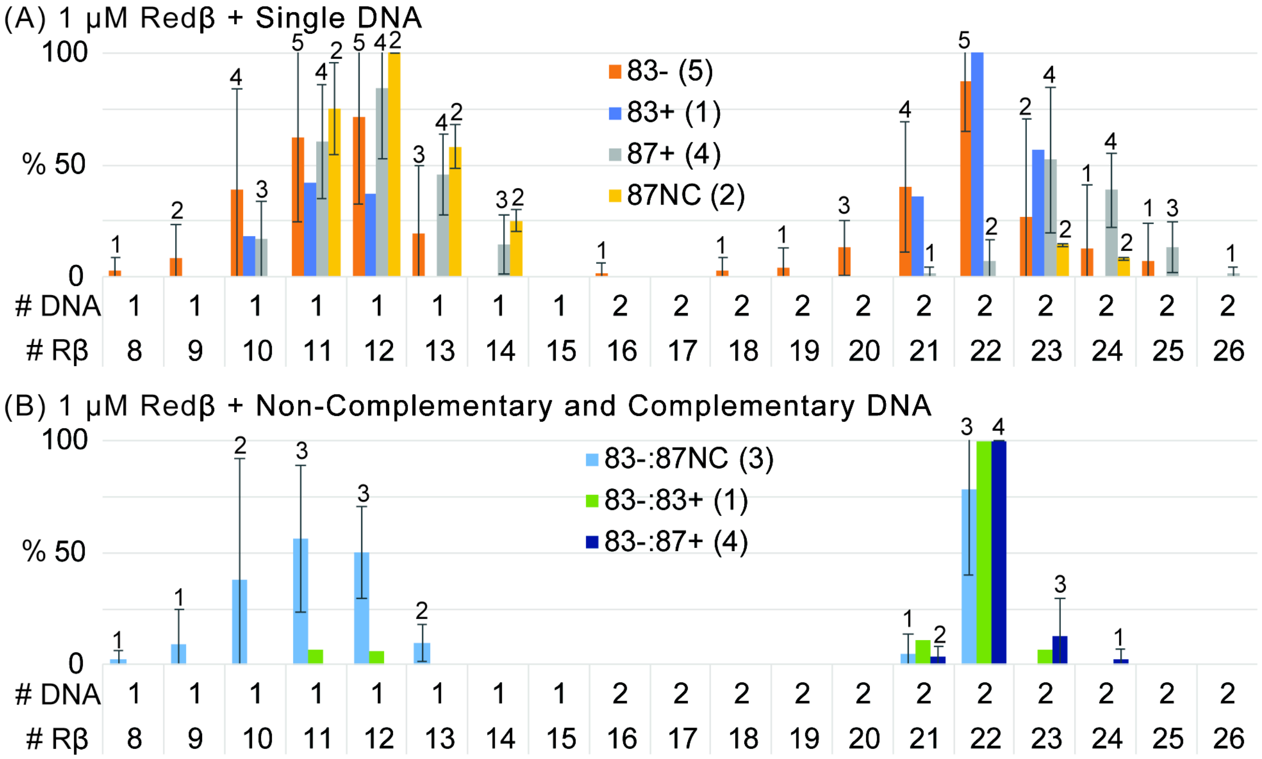

Figure S19. Averaged bar charts of the distribution of oligomers observed in the absence or presence of DNA. The plots give the average relative intensity of each oligomeric species observed when Redβ was mixed with (**A**) a single DNA, or (**B**) two DNAs added sequentially. The error bars represent the standard deviations and the number in parentheses gives the number of spectra for each sample that were averaged. The number above each bar indicates the number of deconvoluted spectra in which the corresponding oligomer was observed. If a mass spectrum did not contain an oligomer, then its intensity was recorded as 0 in calculations of the average and standard deviation. Notice that the variability in the intensities of oligomers observed for Redβ mixed with a single DNA (A) or non-complementary DNAs (B, light blue) is large, while the variability in oligomers observed for Redβ mixed with two complementary DNAs (B, dark blue or green) is much lower. All mass spectra for the data reported in the figure were collected with a collision voltage of 60, which allowed for the peaks in the spectra to be resolvable.

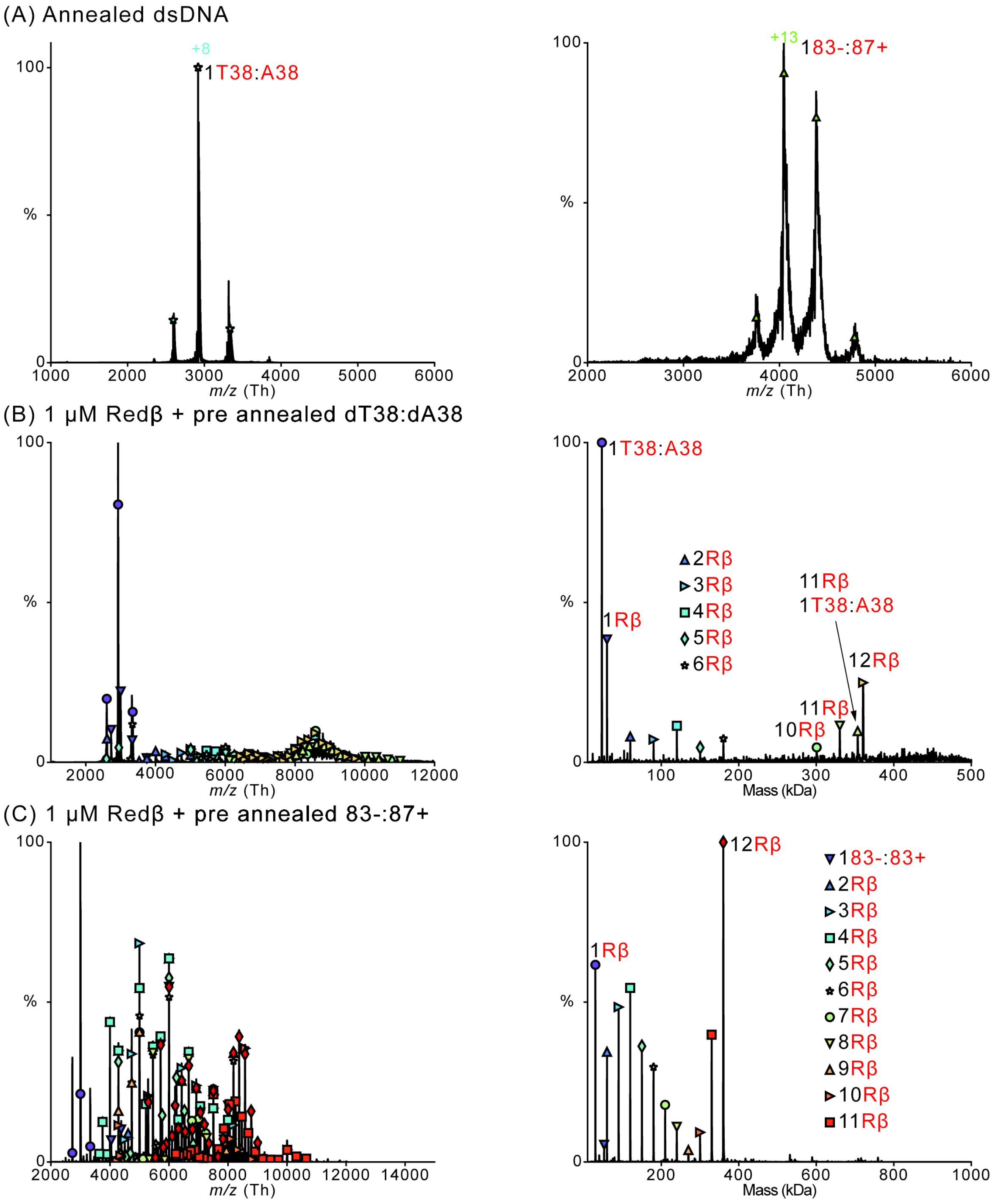

Figure S20. Binding to pre-formed dsDNA. (**A**) Mass spectra of annealed dT38:dA38 DNA (left) and annealed 83-:87+ DNA (right). (**B**) Mass spectrum (left) and zero-charge mass spectrum (right) of Redβ mixed with pre-formed dT38:dA38 resulted in predominantly free protein and DNA. (**C**) Mass spectrum (left) and zero-charge mass spectrum (right) of Redβ mixed with pre-formed 83-:87+ resulted in the appearance of free protein and DNA.

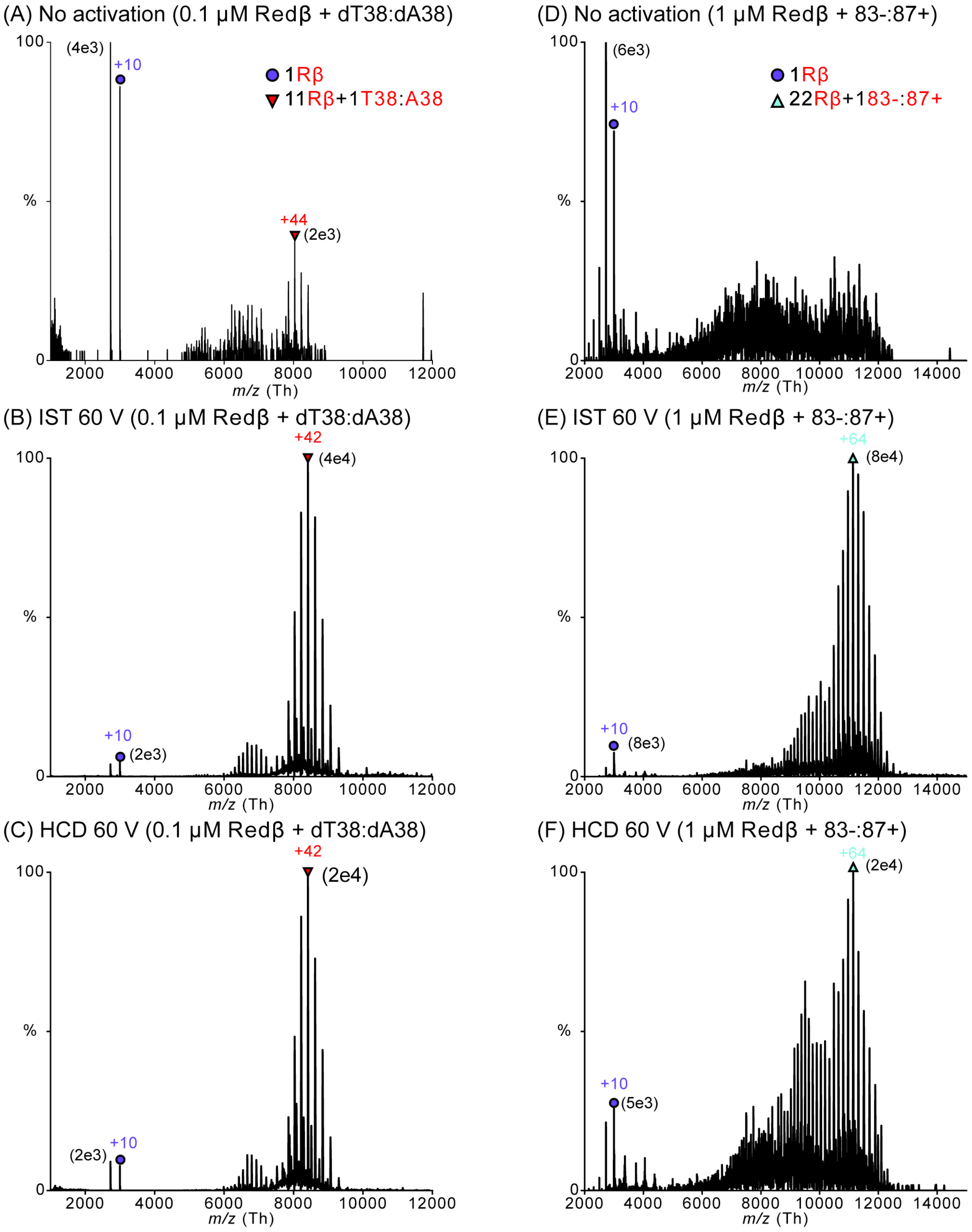

(A) No activation (0.1 μM Redβ + dT38:dA38)
1Rβ
11Rβ+1T38:A38
(4e3)
+10
+44
(2e3)
(B) IST 60 V (0.1 μM Redβ + dT38:dA38)
+42
(4e4)
+10
(2e3)
(C) HCD 60 V (0.1 μM Redβ + dT38:dA38)
+42
(2e4)
(2e3)
+10
(D) No activation (1 μM Redβ + 83-:87+)
1Rβ
22Rβ+183-:87+
(6e3)
+10
(E) IST 60 V (1 μM Redβ + 83-:87+)
+64
(8e4)
+10
(8e3)
(F) HCD 60 V (1 μM Redβ + 83-:87+)
+64
(2e4)
+10
(5e3)
%
m/z (Th)

(G) No activation (1 μM Redβ)

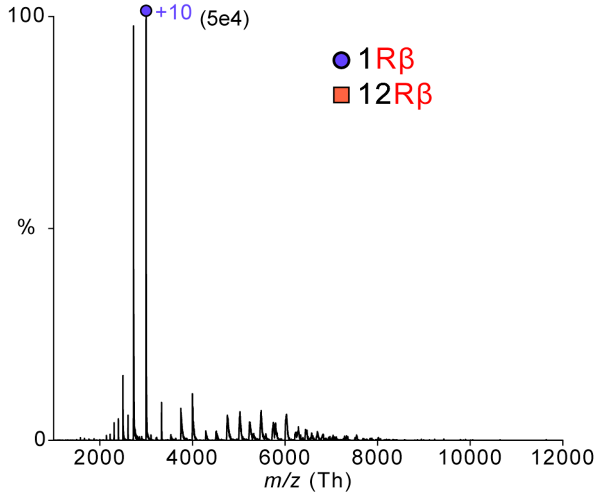

(H) IST 60 V (1 μM Redβ)

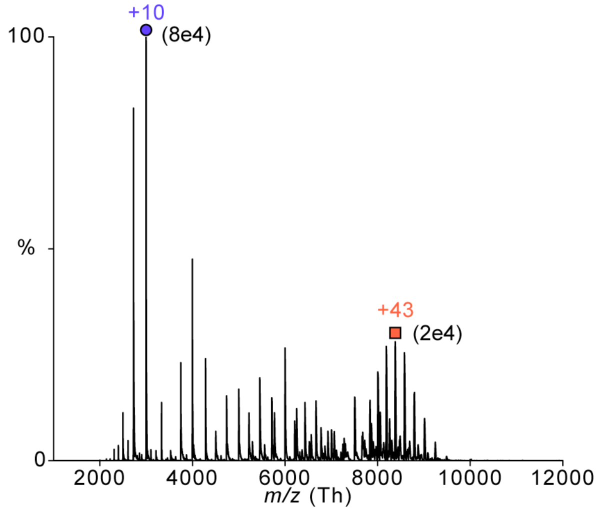

(I) HCD 60 V (1 μM Redβ)

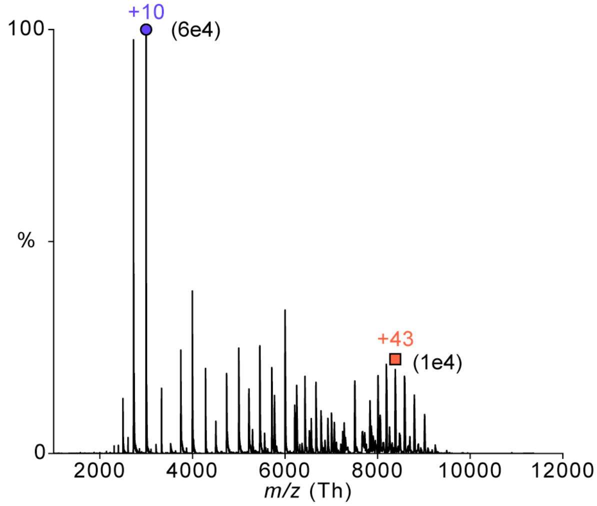

Figure S21. Mass spectra showing the effects of two methods for activation. Mass spectra of 0.1 μM Redβ + 0.4 μM nt dT38:dA38 at (**A**) no activation, (**B**) IST 60 V, (**C**) HCD 60 V. Mass spectra of 1 μM Redβ + 4 μM nt 83-:87+ at (**D**) no activation, (**E**) IST 60 V, (**F**) HCD 60 V. Mass spectra of 1 μM Redβ at (**G**) no activation, (**H**) IST 60 V, (**I**) HCD 60 V. The +10 charge state for Redβ monomer and a single charge state for the dominant complex in each spectrum are displayed for reference. The intensity value at the low and high m/z species is also labeled.

All potential self-annealing sites are marked in red (allowing 1 mis-match):

```
5' TCGGATCTCGTTGCATCTAGCGTCCGTTTACTGTGTCT                              3'
3'                          TCTGTGTCATTTGCCTGCGATCTACGTTGCTCTAGGCT     5'

5'                             TCGGATCTCGTTGCATCTAGCGTCCGTTTACTGTGTCT 3'
3' TCTGTGTCATTTGCCTGCGATCTACGTTGCTCTAGGCT                             5'

5'            TCGGATCTCGTTGCATCTAGCGTCCGTTTACTGTGTCT                  3'
3' TCTGTGTCATTTGCCTGCGATCTACGTTGCTCTAGGCT                             5'

5'   TCGGATCTCGTTGCATCTAGCGTCCGTTTACTGTGTCT                           3'
3' TCTGTGTCATTTGCCTGCGATCTACGTTGCTCTAGGCT                             5'
```

All potential self-annealing sites are marked in red (allowing 1 mis-match):

```
5'            GCGTTATCTCGCCATTGTGTTATCTGTCATTGTGCGTCGC          3'
3' CGCTGCGTGTTACTGTCTATTGTGTTACCGCTCTATTGCG                     5'

5' GCGTTATCTCGCCATTGTGTTATCTGTCATTGTGCGTCGC                     3'
3'     CGCTGCGTGTTACTGTCTATTGTGTTACCGCTCTATTGCG                 5'

5' GCGTTATCTCGCCATTGTGTTATCTGTCATTGTGCGTCGC                     3'
3'     CGCTGCGTGTTACTGTCTATTGTGTTACCGCTCTATTGCG                 5'

5' GCGTTATCTCGCCATTGTGTTATCTGTCATTGTGCGTCGC                     3'
3'              CGCTGCGTGTTACTGTCTATTGTGTTACCGCTCTATTGCG        5'

5' GCGTTATCTCGCCATTGTGTTATCTGTCATTGTGCGTCGC                     3'
3'                    CGCTGCGTGTTACTGTCTATTGTGTTACCGCTCTATTGCG 5'
```

All potential self-annealing sites are marked in red (allowing 1 mis-match):

```
5'                                                    TTGCATATTTAAAACATGTTGAGCTACAGCACCAGATTCAGCAATTAAGCTCTAAGCCATCCGCAAAAATGACCTCTTATCAA      3'
3' AACTATTCTCCAGTAAAAACGCCTACCGAATCTCGAATTAACGACTTAGACCACGACATCGAGTTGTACAAAATTTATACGTT                                                     5'

5'                                                               TTGCATATTTAAAACATGTTGAGCTACAGCACCAGATTCAGCAATTAAGCTCTAAGCCATCCGCAAAAATGACCTCTTATCAA 3'
3' AACTATTCTCCAGTAAAAACGCCTACCGAATCTCGAATTAACGACTTAGACCACGACATCGAGTTGTACAAAATTTATACGTT                                                     5'

5'            TTGCATATTTAAAACATGTTGAGCTACAGCACCAGATTCAGCAATTAAGCTCTAAGCCATCCGCAAAAATGACCTCTTATCAA                                          3'
3' AACTATTCTCCAGTAAAAACGCCTACCGAATCTCGAATTAACGACTTAGACCACGACATCGAGTTGTACAAAATTTATACGTT                                                     5'

5'          TTGCATATTTAAAACATGTTGAGCTACAGCACCAGATTCAGCAATTAAGCTCTAAGCCATCCGCAAAAATGACCTCTTATCAA                                            3'
3' AACTATTCTCCAGTAAAAACGCCTACCGAATCTCGAATTAACGACTTAGACCACGACATCGAGTTGTACAAAATTTATACGTT                                                     5'

5'          TTGCATATTTAAAACATGTTGAGCTACAGCACCAGATTCAGCAATTAAGCTCTAAGCCATCCGCAAAAATGACCTCTTATCAA                                            3'
3' AACTATTCTCCAGTAAAAACGCCTACCGAATCTCGAATTAACGACTTAGACCACGACATCGAGTTGTACAAAATTTATACGTT                                                     5'

5'                                                                TTGCATATTTAAAACATGTTGAGCTACAGCACCAGATTCAGCAATTAAGCTCTAAGCCATCCGCAAAAATGACCTCTTATCAA 3'
3' AACTATTCTCCAGTAAAAACGCCTACCGAATCTCGAATTAACGACTTAGACCACGACATCGAGTTGTACAAAATTTATACGTT                                                     5'

5'                          TTGCATATTTAAAACATGTTGAGCTACAGCACCAGATTCAGCAATTAAGCTCTAAGCCATCCGCAAAAATGACCTCTTATCAA                            3'
3' AACTATTCTCCAGTAAAAACGCCTACCGAATCTCGAATTAACGACTTAGACCACGACATCGAGTTGTACAAAATTTATACGTT                                                     5'

5'                          TTGCATATTTAAAACATGTTGAGCTACAGCACCAGATTCAGCAATTAAGCTCTAAGCCATCCGCAAAAATGACCTCTTATCAA                            3'
3' AACTATTCTCCAGTAAAAACGCCTACCGAATCTCGAATTAACGACTTAGACCACGACATCGAGTTGTACAAAATTTATACGTT                                                     5'

5'      TTGCATATTTAAAACATGTTGAGCTACAGCACCAGATTCAGCAATTAAGCTCTAAGCCATCCGCAAAAATGACCTCTTATCAA                                                3'
3' AACTATTCTCCAGTAAAAACGCCTACCGAATCTCGAATTAACGACTTAGACCACGACATCGAGTTGTACAAAATTTATACGTT                                                     5'

5'      TTGCATATTTAAAACATGTTGAGCTACAGCACCAGATTCAGCAATTAAGCTCTAAGCCATCCGCAAAAATGACCTCTTATCAA                                                3'
3' AACTATTCTCCAGTAAAAACGCCTACCGAATCTCGAATTAACGACTTAGACCACGACATCGAGTTGTACAAAATTTATACGTT                                                     5'

5' TTGCATATTTAAAACATGTTGAGCTACAGCACCAGATTCAGCAATTAAGCTCTAAGCCATCCGCAAAAATGACCTCTTATCAA                                                     3'
3'   AACTATTCTCCAGTAAAAACGCCTACCGAATCTCGAATTAACGACTTAGACCACGACATCGAGTTGTACAAAATTTATACGTT                                                   5'

5' TTGCATATTTAAAACATGTTGAGCTACAGCACCAGATTCAGCAATTAAGCTCTAAGCCATCCGCAAAAATGACCTCTTATCAA                                                     3'
3'   AACTATTCTCCAGTAAAAACGCCTACCGAATCTCGAATTAACGACTTAGACCACGACATCGAGTTGTACAAAATTTATACGTT                                                   5'

5'            TTGCATATTTAAAACATGTTGAGCTACAGCACCAGATTCAGCAATTAAGCTCTAAGCCATCCGCAAAAATGACCTCTTATCAA                                          3'
3' AACTATTCTCCAGTAAAAACGCCTACCGAATCTCGAATTAACGACTTAGACCACGACATCGAGTTGTACAAAATTTATACGTT                                                     5'
```

Figure S22. Self-complementarity analysis of 38NC1- (top left), 40NC3- (top right) and 83- (bottom) ssDNA using Oligo Calc: Oligonucleotide Properties Calculator (http://biotools.nubic.northwestern.edu/OligoCalc.html). Sequences indicated in red indicate stretches of perfect complementarity.
